# MSOAR 2.0: Incorporating tandem duplications into ortholog assignment based on genome rearrangement

**DOI:** 10.1186/1471-2105-11-10

**Published:** 2010-01-06

**Authors:** Guanqun Shi, Liqing Zhang, Tao Jiang

**Affiliations:** 1 Department of Computer Science, University of California, Riverside, CA 92521, USA; 2 Department of Computer Science, Virginia Tech, Blacksburg, VA 24060, USA

## Abstract

**Background:**

Ortholog assignment is a critical and fundamental problem in comparative genomics, since orthologs are considered to be functional counterparts in different species and can be used to infer molecular functions of one species from those of other species. MSOAR is a recently developed high-throughput system for assigning one-to-one orthologs between closely related species on a genome scale. It attempts to reconstruct the evolutionary history of input genomes in terms of genome rearrangement and gene duplication events. It assumes that a gene duplication event inserts a duplicated gene into the genome of interest at a random location (*i.e.*, the random duplication model). However, in practice, biologists believe that genes are often duplicated by tandem duplications, where a duplicated gene is located next to the original copy (*i.e.*, the tandem duplication model).

**Results:**

In this paper, we develop MSOAR 2.0, an improved system for one-to-one ortholog assignment. For a pair of input genomes, the system first focuses on the tandemly duplicated genes of each genome and tries to identify among them those that were duplicated after the speciation (*i.e.*, the so-called inparalogs), using a simple phylogenetic tree reconciliation method. For each such set of tandemly duplicated inparalogs, all but one gene will be deleted from the concerned genome (because they cannot possibly appear in any one-to-one ortholog pairs), and MSOAR is invoked. Using both simulated and real data experiments, we show that MSOAR 2.0 is able to achieve a better sensitivity and specificity than MSOAR. In comparison with the well-known genome-scale ortholog assignment tool InParanoid, Ensembl ortholog database, and the orthology information extracted from the well-known whole-genome multiple alignment program MultiZ, MSOAR 2.0 shows the highest sensitivity. Although the specificity of MSOAR 2.0 is slightly worse than that of InParanoid in the real data experiments, it is actually better than that of InParanoid in the simulation tests.

**Conclusions:**

Our preliminary experimental results demonstrate that MSOAR 2.0 is a highly accurate tool for one-to-one ortholog assignment between closely related genomes. The software is available to the public for free and included as online supplementary material.

## Background

*Orthologs *and *paralogs *are two different types of homologous genes that differ in the way that they evolved. Orthologs are genes in different species that evolved from a common ancestral gene due to speciation events while paralogs are duplicated genes in the same genome [1]. To better understand the evolutionary process, paralogs are further divided into two subtypes: *outparalogs *and *inparalogs *[2]. With respect to a given speciation event, outparalogs are genes duplicated before the speciation while inparalogs are genes duplicated after the speciation. These concepts as well as the relationship among orthologs, outparalogs and inparalogs are illustrated in Additional file 1, Figure S1, which depicts the evolution of globin genes in human, mouse and rat.

Note that the orthology between two species is in general a many-to-many relationship. In other words, for a pair of genomes, an ortholog group consists of a pair of sets of inparalogs, one from each genome. The inparalogs in one set are co-orthologous to all the inparalogs in the other. For each set of inparalogs on a genome, there usually exists a gene that is the direct descendant of the ancestral gene of such a set, which is referred to as the "true examplar" by Sankoff [3], while the other inparalogs in the set are duplicated from the true examplar gene. Therefore, for each ortholog group, we may select a representative from each set of inparalogs (*e.g.*, the exemplar gene) and define a one-to-one ortholog pair consisting of the two representatives. Such an ortholog pair may contain the two genes, one from each set, that correspond the best in terms of their positions on the genomes [4] or sequence similarity [2]. This allows us to think of orthology as a one-to-one relationship, which could help simplify the discussion in many cases and makes it possible to evaluate an ortholog assignment result against the orthology benchmark defined by gene symbols (which is a one-to-one relationship).

Moreover, the one-to-one orthology relationship is critically used in many comparative genomics studies, such as the reconstruction of accurate gene trees [5], alignment of protein-protein interaction (PPI) networks across multiple species [6], identification of functional orthologs [7], evolutionary, comparative and systematic studies in plants [8], and mapping of biological pathways [9]. (One-to-one orthologs are called "true orthologs" in [7] and "single copy orthologous genes" in [8].) Note that once a one-to-one ortholog pair is specified for an ortholog group, all other pairs of genes from the group will be regarded as false positives (with respect to the one-to-one orthology relationship). In this paper, we are interested in assigning orthologs as a one-to-one relationship. To avoid ambiguity, we will add the prefix "one-to-one" in front of such orthologs.

Clearly, it is easy to identify the one-to-one ortholog pairs between two species if the duplication history of the genes on the two genomes is given (relative to their speciation event). Unfortunately, this evolutionary process is unknown. What we know is all the genes in the contemporary genomes. In order to find the most probable one-to-one ortholog assignment between two genomes, we need to reconstruct the true evolutionary history.

### Existing Work on Ortholog Assignment

There exist many algorithms and tools for ortholog assignment, including the well-known COG system [10], InParanoid [2,11], OrthoMCL [12], HomoloGene [13], TreeFam [14], PhyOP [15], and Ensembl Compara [16], just to name a few. A recent comprehensive review on ortholog assignment tools in the public domain can be found in [17]. The first four tools, *i.e.*, COG, InParanoid, OrthoMCL and HomoloGene, are basically sequence similarity based methods that calculate pairwise similarity scores and employ some simple clustering algorithms to identify ortholog groups. For example, InParanoid assigns *main ortholog pairs *as the pairs of protein sequences with the highest bidirectional BLASTp scores (*i.e.*, *bidirectional best hits*, or *BBHs*), and uses them as "seeds" to identify inparalogs from both species by applying a heuristic clustering algorithm [2]. TreeFam, PhyOP and Ensembl Compara, on the other hand, explicitly reconstruct phylogenetic trees to infer the orthology relationship. Ensembl Compara, in particular, is a computational pipeline that combines some clustering method with phylogenetic tree reconciliation. It provides one-to-one, one-to-many, and many-to-many orthology relationships for more than 30 eukaryotic species [16]. However, none of these methods take gene order and genome rearrangement into account when they assign orthologs. It has been shown that genome rearrangement is very common between two closely related genomes [18-21], and thus the gene order information may help improve the accuracy of ortholog assignment.

By combining both sequence similarity and gene order information, a high-throughput one-to-one or-tholog assignment system called MSOAR [4,22] has recently been developed. The system attempts to reconstruct the evolutionary history of the genes in the input genomes in terms of genome rearrangement and gene duplication events, and tries to minimize the *RD *(rearrangement and duplication) distance under the parsimony principle. MSOAR considers four genome rearrangement events including reversal (*i.e.*, inversion), translocation, fusion, and fission, and assumes that a gene duplication event inserts a duplicated gene into the concerned genome at a random location (*i.e.*, the random duplication model).

**Figure 1:**
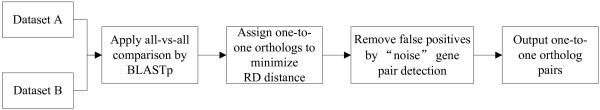
**An outline of MSOAR**.

For the convenience of the reader, an outline of the major algorithmic steps in MSOAR is sketched in Figure 1. In particular, MSOAR attempts to remove false one-to-one ortholog pairs that involve genes randomly duplicated after the speciation in the "noise" gene pair detection step. Such a (false) ortholog pair usually incurs a great cost in the rearrangement distance between the genomes, and thus we would be able to reduce the RD distance by "uncoupling" (*i.e.*, removing) the pair. However, in reality, randomly duplicated genes only account for a part of all duplicated genes. Recent studies have shown that at least 30% of duplicated genes are found next to their original copies (*i.e.*, in tandem positions) [23,24].

### Gene Duplication Models

The importance of gene duplication in molecular evolution is well established [25,26]. However, the biological mechanism behind gene duplication has been unknown for quite many years. Recently, biologists proposed three different mechanisms for gene duplication based on the size of the duplication and whether they involve an RNA intermediate [27,28]: retrotransposition, tandem duplication, and genome duplication.

Retrotransposition describes the integration of a reverse transcribed mRNA into the genome in a random manner (see Additional file 1, Figure S2), and is the cause of random duplications. Tandem duplication is one of the possible outcomes of "unequal crossover", which results from the homologous recombination between paralogous sequences (see Additional file 1, Figure S3). As a result, genes are duplicated next to their original copies in tandem arrays on the genome, which are known as *TAGs *(*i.e.*, *tandemly arrayed genes*) [23]. Genome duplication is probably due to the lack of disjunction between daughter chromosomes after DNA replication, and occurs more in plants than in animals. Recent studies show that there is another type of large-scale duplications, segmental duplication, which involves 1 kb~400 kb nucleotides, though the molecular mechanism of segmental duplication is still unclear [27].

### An Improved Ortholog Assignment System

**Figure 2:**
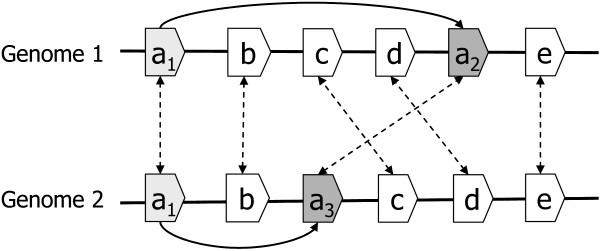
**Genes *a*_2 _and *a*_3 _are randomly duplicated from gene *a*_1_**.

**Figure 3:**
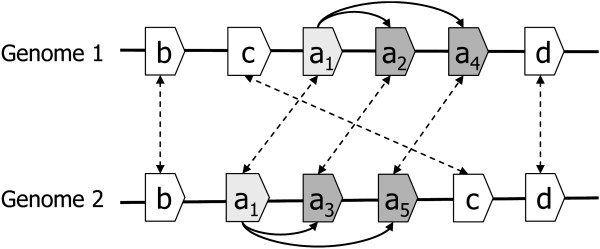
**Genes *a*_2_, *a*_3_, *a*_4 _and *a*_5 _are tandemly duplicated from gene *a*_1_**.

Although MSOAR is able to identify most randomly duplicated inparalogs in the "noise" gene pair detection step, it is incapable of catching inparalogs that are produced by tandem duplications, which prevents MSOAR from identifying false one-to-one ortholog pairs that involve two duplicated inparalogs in TAGs from both genomes. See the examples in Figures 2 and 3.

In Figures 2 and 3, we assume that the genes with the same letter from the two genomes represent true one-to-one orthologs, and all duplications happened after the speciation in both genomes. For example, in Figure 3, (*a*_1, _*a*_1_) is a true one-to-one ortholog pair while (*a*_2_, *a*_3_) and (*a*_4_, *a*_5_) are not. The genes *a*_2 _and *a*_3 _in Figure 2 and genes *a*_2_, *a*_3_, *a*_4 _and *a*_5 _in Figure 3 are all duplicated from gene *a*_1 _after the speciation, and thus are inparalogs of *a*_1_. In both cases, MSOAR first tries to assign one-to-one orthology between all pairs of genes and calculates the RD distance between the two genomes. However, in the "noise" gene pair detection step, MSOAR is able to identify the false one-to-one ortholog pair (*a*_2_, *a*_3_) in Figure 2 since the RD distance between the two genomes will decrease by 1 (*i.e.*, 3 fewer reversals and 2 more duplications) if this pair is removed. However, if the duplicated genes are in TAGs, as shown in Figure 3, removing any of the pairs (*a*_2_, *a*_3_) and (*α*_4_, *a*_5_) will not affect the number of reversals but will increase the number of duplications by 2, thus increasing the RD distance between the two genomes. Since MSOAR tries to find an assignment to minimize the RD distance between the two genomes, it will correctly identify the false one-to-one ortholog pair (*a*_2_, *a*_3_) in Figure 2 while incorrectly keep both false one-to-one ortholog pairs (*a*_2_, *a*_3_) and (*a*_4_, *a*_5_) in Figure 3 in the assignment.

In this paper, we incorporate the tandem duplication model into MSOAR, and develop an improved system, simply called MSOAR 2.0, to assign one-to-one ortholog pairs between two genomes. The idea is to consider tandemly duplicated genes first and try to identify the inparalogy relationship among them using a simple phylogenetic tree reconciliation method. For each set of inparalogs (on the same genome), all but one gene will be deleted from the concerned genome before MSOAR is invoked. Our experimental results demonstrate that this pre-processing step could indeed remove many false positives correctly and thus greatly improve the specificity of MSOAR.

## Results and Discussion

**Figure 4:**
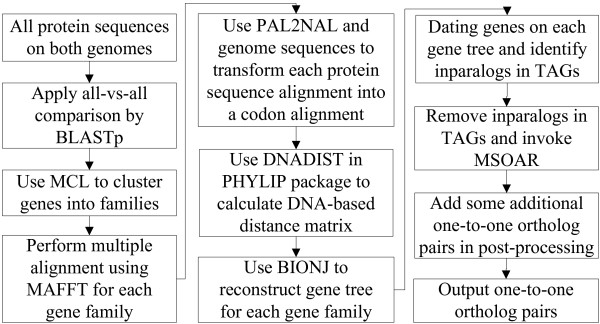
**An outline of MSOAR 2.0**.

The system MSOAR 2.0 has been implemented as a C++ application on a standard Linux system. Its main steps, as outlined in Figure 4, include: (i) the construction of gene families using a clustering approach, (ii) the identification of inparalogs in TAGs using a simple phylogenetic analysis, (iii) the invocation of MSOAR after removing inparalogs in TAGs, and (iv) the identification of additional one-to-one ortholog pairs in a post-processing step. The detailed description of each of the main steps is given in the Methods section. The software is available to the public for free and included as an online supplementary material in Additional file 2.

In order to test the performance of MSOAR 2.0, we apply it to both simulated and real data, and compare our results with MSOAR [4], the popular ortholog assignment tool InParanoid [11], Ensembl ortholog database [16] and the orthologs extracted from the whole-genome multiple alignment program MultiZ [29].

### Simulation Results

To assess the accuracy of one-to-one ortholog assignment, we simulate two input (single-chromosomal) genomes by using duplications, reversals, and point mutations. The simulation is controlled by a set of 4 parameters (*k*, *p*, *α*, *β*), where *k *denotes the number of duplications in the ancestral genome before the speciation, *p *is the total number of genome-level evolutionary events (*i.e.*, duplications and reversals) on each genome after the speciation, *α *is the percentage of duplications among the *p *events, and *β *is the percentage of tandem duplications among all duplications. A detailed description of the actual simulation process is given in the Methods section.

After generating two input genomes, we run MSOAR 2.0, MSOAR, and InParanoid separately. From the outputs of the three programs, we can easily compare their prediction accuracies in terms of sensitivity (*i.e.*, the number of true positive pairs assigned divided by the total number of assignable true positive pairs) and specificity (*i.e.*, the number of true positive pairs assigned divided by the total number of assigned pairs). Note that InParanoid actually outputs ortholog groups. For each ortholog group, we take the first pair of genes in the group as the one-to-one ortholog pair (which is referred to as the *main ortholog pair *in [2]).

**Figure 5:**
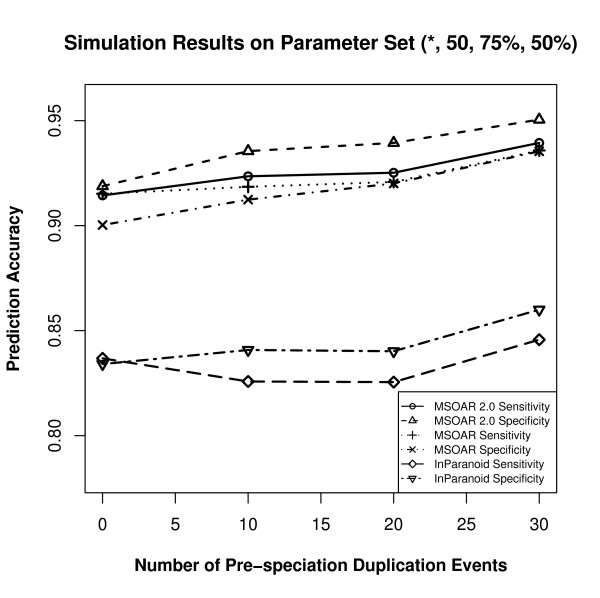
Simulation results on the parameter set (*, 50, 75%, 50%) where the parameter *k *is varied.

**Figure 6:**
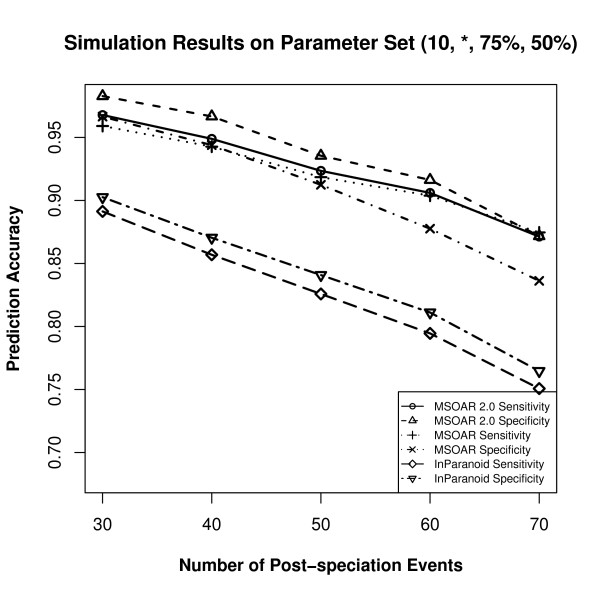
Simulation results on the parameter set (10, *, 75%, 50%) where the parameter *p *is varied.

**Figure 7:**
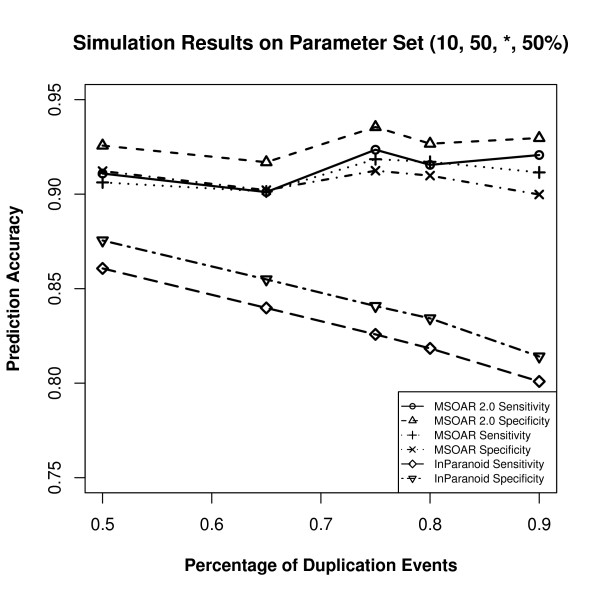
Simulation results on the parameter set (10, 50, *, 50%) where the parameter *α *is varied.

**Figure 8:**
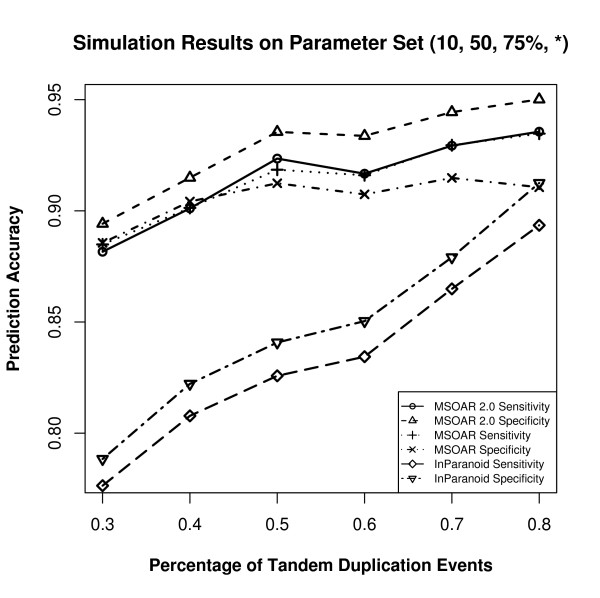
Simulation results on the parameter set (10, 50, 75%, *) where the parameter *β *is varied.

Since different parameters produce different input genomes, which may affect the prediction accuracies of the three programs, the parameters are varied as follows. We use a default parameter set and change the value of one parameter at one time. Based on recent studies on the relative ratios of various genome-level evolutionary events [23,30], we choose to use (10, 50, 75%, 50%) as our default parameter set. For each parameter set, 50 random datasets are simulated and the average prediction accuracies of the three programs are calculated. The performance of the three programs on various parameter sets are shown in Figures 5, 6, 7, and 8.

From Figures 5, 6, 7 and 8, we can see that parameter *k *has little effect on the prediction accuracies of the three programs as it only defines the number of outparalogs. Parameter *p*, on the other hand, has a great impact on the performance of all the programs. With the increase of *p*, the prediction accuracies of all the three programs sharply decrease. This is because when the number of evolutionary events increases, it is more difficult for MSOAR and MSOAR 2.0 to correctly reconstruct the evolutionary history based on the parsimony principle. Also orthologous genes may become less similar to each other for InParanoid to correctly identify them based on sequence similarity. Parameter *α *defines the ratio between duplications and reversals. As *α *goes up, the number of duplications increases while the number of reversals decreases. It becomes easier for MSOAR and MSOAR 2.0 to correctly identify reversals and assign one-to-one orthologs while it becomes harder for InParanoid to differentiate main orthologs from their duplicated inparalogs due to the large number of duplications. Parameter *β *defines the ratio between tandem duplications and random duplications.

As the ratio of tandem duplications goes up, the sensitivities of all three programs increase. This is due to the definition of true positives (TPs) in the simulation test. For each pair of orthologous TAGs from the two genomes, any pair of genes consisting of one gene from each TAG could be counted as a TP since these pairs are indistinguishable. However, at most one pair in the orthologous TAGs is counted as a TP. So, when the number of tandem duplications increases, all three programs output more TPs. As for specificity, since MSOAR 2.0 removes most of the inparalogs in TAGs based on the phylogenetic analysis and only the main ortholog pairs found by InParanoid are considered, the two programs do not introduce more false positives (FPs) when the number of tandem duplications increases. Thus, the specificities of these two programs both increase. On the other hand, MSOAR may tend to assign more than one one-to-one ortholog pairs between two orthologous TAGs. This results in more FPs for MSOAR and an almost unchanged specificity.

The simulation results show that, in general, MSOAR 2.0 and MSOAR are more accurate than InParanoid in terms of both sensitivity and specificity on randomly simulated data. The sensitivity of MSOAR 2.0 is slightly better than that of MSOAR while its specificity is significantly (2% ~ 5%) higher than that of MSOAR. Note that the design of our simulation study was rather simplistic and the genomes simulated were not of real genome sizes. Hence, the above simulation results might not faithfully reflect the relative performance of InParanoid, MSOAR and MSOAR 2.0 on real data.

### Real Data Experiments

In order to evaluate the performance of MSOAR 2.0 on real data, we apply MSOAR 2.0 to several real datasets. Since the human genome is the best annotated genome and has been used as the reference genome to assign gene symbols for other species, we use it as the "center" in our pairwise comparisons and compare it with four other mammalian genomes, mouse, rat, chimpanzee, and macaque that have been completely sequenced. The detailed procedure for downloading and pre-processing these genomes is described in the Methods section.

#### Results on Human, Mouse and Rat

For the one-to-one ortholog assignments between human and mouse and between human and rat, Table 1 shows the contributions of each major step in MSOAR 2.0. The phylogenetic analysis step is able to identify more than 1,000 duplicated inparalogs in TAGs in each species (1,232/2,675 for human-mouse and 1,354/2,216 for human-rat), and remove most of them before MSOAR is invoked. Then one-to-one orthology is assigned by MSOAR on the remaining genes on each genome. Finally, in the post-processing step, MSOAR 2.0 is able to catch a few hundred one-to-one ortholog pairs (113 for human-mouse and 112 for human-rat) from the "gaps" between consecutive orthologous blocks on each genome.

**Table 1:** Contributions of the major steps in MSOAR 2.0.

Pair of Species	Inparalogs in TAGs Identified by Phylogenetic Analysis	Orthologs Assigned by MSOAR	Orthologs Assigned after Post-Processing
human vs mouse	1,232/2,675	16,661	16,774

human vs rat	1,354/2,216	15,830	15,942

In order to validate the prediction results of MSOAR 2.0, we choose to use gene symbols. Gene symbols are used by researchers to refer to a specific gene of interest across species. Each symbol for a species should be unique and each gene within a genome should be given only one approved gene symbol [31]. The nomenclature of a gene is done by the nomenclature committees for each species. At present, there are only three official nomenclature committees in the world, for human, mouse, and rat respectively. So only these three species have official gene symbols. To obtain the most accurate gene symbol lists, we download the most recent gene symbols for human, mouse, rat from HGNC http://www.genenames.org/, MGI http://www.informatics.jax.org/, and RGD http://rgd.mcw.edu/ respectively, all of which are the official nomenclature committees for the involved species. Note that since some gene symbols were assigned using information from some orthology databases, we should take the validation results based on gene symbols with a grain of salt. However, everything considered, gene symbols may still be the best available benchmark for validating genome-wide one-to-one ortholog assignment results.

To compare the performance of MSOAR 2.0 with that of MSOAR, InParanoid, the Ensembl ortholog database, and MultiZ, we consider the gene symbols of each output ortholog pair. Some genes may not have official gene symbols. Some symbols may not be meaningful, *e.g.*, when they are composed of "LOC" and gene ID, or when the gene functions have not yet been validated. In the latter case, the genes only have transcript identifiers (*e.g.*, gene symbols with the prefix "OTTMUSG" or the suffix "RIK" in the mouse genome). For each pair of orthologs, if both genes have identical official gene symbols, we count it as a true positive pair (*i.e.*, *TP*). If the genes have different official gene symbols, we count it as a false positive pair (*i.e.*, *FP*). If only one gene in the pair has an official gene symbol and another gene on the other genome (which is not in the pair) has the same gene symbol, then this pair is also considered as a false positive pair. For all other cases, we deem the pair as an unknown pair and ignore it in the accuracy assessment. We also calculate the assignable true one-to-one ortholog pairs between two species by counting the number of identical gene symbols. The performance of the five methods validated using gene symbols is shown in Table 2. The actual one-to-one ortholog assignment results of MSOAR 2.0 as well as the raw data and the MSOAR 2.0 software source code can be downloaded from the MSOAR website http://msoar.cs.ucr.edu/.

**Table 2:** Comparison of the performance of five programs using gene symbol validation.

Pair of Species	Program	Assignable	Total Assigned	True Positives	Unknowns	Sensitivity	Specificity
human vs mouse	InParanoid	14,341	16,058	13,216	1,394	92.16%	90.13%
	Ensembl	14,341	20,670	13,619	2,850	94.97%	76.43%
	MultiZ	14,341	16,543	13,136	1,433	91.60%	86.94%
	MSOAR	14,341	16,769	13,528	1,554	94.33%	88.91%
	MSOAR 2.0	14,341	16,774	13,625	1,551	95.01%	89.50%

human vs mouse	InParanoid	12,688	15,197	11,750	1,529	92.61%	85.97%
	Ensembl	12,688	18,814	12,004	2,490	94.61%	73.54%
	MultiZ	12,688	16,102	11,600	1,570	91.42%	79.82%
	MSOAR	12,688	15,883	11,970	1,723	94.34%	84.53%
	MSOAR 2.0	12,688	15,942	12,085	1,765	95.25%	85.24%

In order to assess the accuracy of InParanoid, we take the first pair of genes in each ortholog group (*i.e.*, the main ortholog pair of the group) as a one-to-one ortholog pair. For the Ensembl ortholog database, we directly download all the ortholog pairs from Ensembl Biomart Browser, which includes one-to-one, one-to-many, and many-to-many orthology relationships. In order to extract the orthology information from MultiZ, we download the whole-genome multiple alignment for human, mouse and rat from UCSC genome browser, and map the annotated genes to the alignment based on their coordinates on each genome.

Table 2 suggests that MSOAR 2.0 achieves the best sensitivity among the five programs although its specificity is slightly worse than that of InParanoid. A detailed analysis on the differences among the ortholog assignment results by these programs is given in Table 3.

**Table 3:** Differences between the ortholog pairs assigned by MSOAR 2.0 and those by the other programs.

Pair of Species	MSOAR 2.0 vs InParanoid		MSOAR 2.0 vs Ensembl		MSOAR 2.0 vs MSOAR	
			
	TPs in MSOAR 2.0 but not in **InParanoid**^*a*^	Not **BBHs**^*b*^	FPs in Ensembl but not in **MSOAR 2.0**^*c*^	Inparalogs in **TAGs**^*d*^	FPs in MSOAR but not in **MSOAR 2.0**^*e*^	Inparalogs in **TAGs**^*f*^
human vs mouse	487	408	2,997	2,664	330	312

human vs rat	429	400	2,681	2,366	311	299

(a) This column lists the number of TPs found by MSOAR 2.0 but missed by InParanoid. (b) This column lists the number of TPs in the previous column that are not BBHs. (c) This column lists the number of FPs found by Ensembl but not by MSOAR 2.0. (d) This column lists the number of FPs in the previous column that are inparalogs occurring in TAGs. (e) This column lists the number of FPs found by MSOAR but not by MSOAR 2.0. (f) This column lists the number of FPs in the previous column that are inparalogs occurring in TAGs.

**Figure 9:**
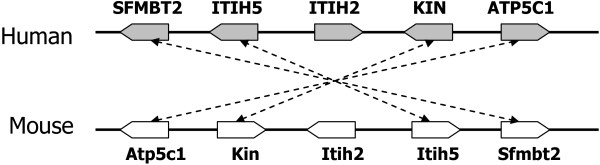
**A real example of non-BBH true one-to-one ortholog pairs in the human-mouse comparison caught by MSOAR 2.0 in the post-processing step**. Four one-to-one ortholog pairs were assigned by MSOAR between two corresponding orthologous blocks on human chromosome 10 (7,244,255 bp-7,900,507 bp) and mouse chromosome 2 (9,977,663 bp-10,636,794 bp). The genes ITIH2 and Itih2 were not assigned orthology by MSOAR, since ITIH2 is not among the top hits of Itih2. However, because Itih2 is the best hit of ITIH2 and the genes are located in corresponding "gaps", MSOAR 2.0 outputs them as an additional one-to-one ortholog pair.

Since InParanoid is a sequence similarity based method, it produces ortholog groups solely based on sequence similarity. In order to compare the performance of InParanoid with MSOAR 2.0 properly, we take the first pair of each ortholog group output by InParanoid, *i.e.*, the main ortholog pair [2], as the one-to-one ortholog pairs assigned by InParanoid. As a result, all of the main ortholog pairs assigned by InParanoid are BBHs. Although many of the true one-to-one ortholog pairs may be indeed BBHs, some of them are not. In fact, more than 80% of the true one-to-one ortholog pairs assigned by MSOAR 2.0 but missed by InParanoid in the human-mouse and human-rat comparisons (408/487 for human-mouse and 400/429 for human-rat) are not BBHs as shown in Table 3 (the first two columns). An example from the human-mouse comparison can be seen in Figure 9. Here, the true one-to-one ortholog pair (ITIH2, Itih2) is missed by InParanoid since ITIH2 and Itih2 are not BBHs. But MSOAR 2.0 was able to catch this pair correctly.

**Figure 10:**
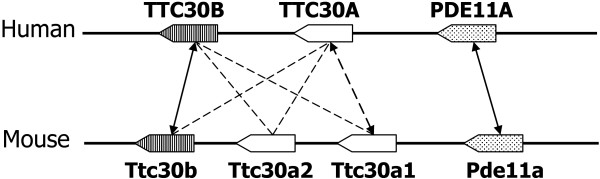
**Comparison of ortholog assignments made by Ensembl, MSOAR and MSOAR 2.0 for the two segments of human chromosome 2 (178,123,219 bp-178,685,428 bp) and mouse chromosome 2 (75,773,906 bp-76,192,000 bp)**. Among the 7 pairs of genes illustrated in the figure, only (TTC30B, Ttc30b) and (PDE11A, Pde11a) are known one-to-one ortholog pairs according to gene symbols, as indicated by solid lines. Since the Ensembl ortholog database includes many-to-many relationship, it outputs 7 ortholog pairs, *i.e.*, (TTC30B, Ttc30b), (TTC30B, Ttc30a2), (TTC30B, Ttc30a1), (TTC30A, Ttc30b), (TTC30A, Ttc30a2), (TTC30A, Ttc30a1), and (PDE11A, Pde11a), introducing 5 false ortholog pairs, as indicated by dashed lines. MSOAR assigns three one-to-one ortholog pairs as indicated by the arrows in the figure, *i.e.*, (TTC30B, Ttc30b), (TTC30A, Ttc30a1), and (PDE11A, Pde11a), including one false one-to-one ortholog pair. MSOAR 2.0, however, identifies TTC30A as an inparalog of TTC30B on the human genome and Ttc30a2 and Ttc30a1 as inparalogs of Ttc30b on the mouse genome during the phylogenetic analysis of TAGs, and removes them before invoking MSOAR. Thus, MSOAR 2.0 only outputs two one-to-one ortholog pairs, *i.e.*, (TTC30B, Ttc30b) and (PDE11A, Pde11a), both of which are true positives.

While we mainly focus on finding the one-to-one orthology relationship between two genomes, the Ensembl ortholog database presents orthology in general as a many-to-many relationship. Thus, for each ortholog group, it outputs all pairs of genes consisting of one gene from one genome and another from the other. As a result, the specificity of the Ensembl ortholog database is quite low because each large ortholog group may result in many false positives. (Hence, our measure of specificity is unfair to Ensembl since it treats orthology as a one-to-one relationship.) What is interesting is that even though it outputs a large number of ortholog pairs, its sensitivity is still a little bit worse than that of MSOAR 2.0 in both human-mouse and human-rat comparisons as shown in Table 2. It is interesting to observe that most of the false positive pairs output by Ensembl but not by MSOAR 2.0 (*i.e.*, 2,664/2,997 for the human-mouse comparison and 2,366/2,681 for the human-rat comparison) were actually found by MSOAR 2.0 to be inparalogs that appear in some TAGs, as shown in Table 3 (the two middle columns). See Figure 10 for an example of inparalogs in TAGs caught by MSOAR 2.0.

The last two columns of Table 3 clearly demonstrate that MSOAR 2.0 achieves a better specificity than MSOAR because of its treatment of TAGs, since most of the false positives output by MSOAR but not by MSOAR 2.0 (312/330 and 299/311 for the human-mouse and human-rat comparisons, respectively) were identified as inparalogs in TAGs by MSOAR 2.0. For a detailed example where MSOAR 2.0 is able to catch false positives output by MSOAR, see again Figure 10.

MultiZ is generally viewed as a whole-genome multiple alignment program, but we can easily extract orthology information from the multiple alignment produced by MultiZ. To compare with the performance of MultiZ in one-to-one ortholog assignment, we download the human, mouse and rat genome alignment by MultiZ from UCSC genome browser, and map the annotated genes to the alignment according to their coordinates on each genome. If a gene contains several regions which are aligned to different locations belonging to different genes on another genome, then it forms a one-to-many orthology relationship and all pairs are counted in the same way as we dealt with the Ensembl ortholog database. Table 2 shows that MultiZ is worse than InParanoid in both sensitivity and specificity. Since both methods are based on sequence similarity, we will not include MultiZ in further comparative studies.

#### Results on Human, Chimpanzee and Macaque

**Figure 11:**
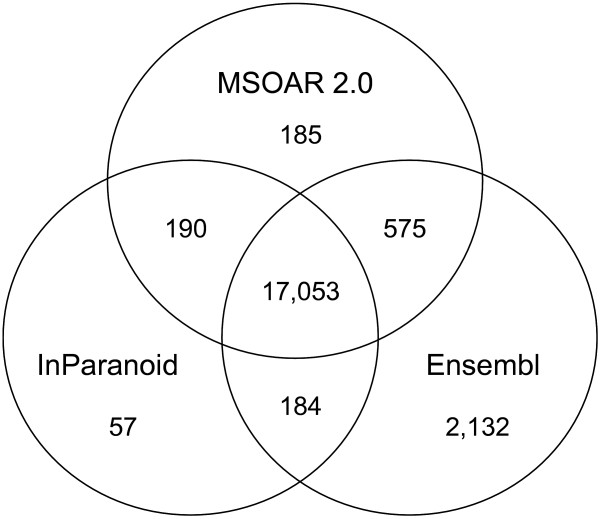
**Orthologs assigned between human and chimpanzee**.

**Figure 12:**
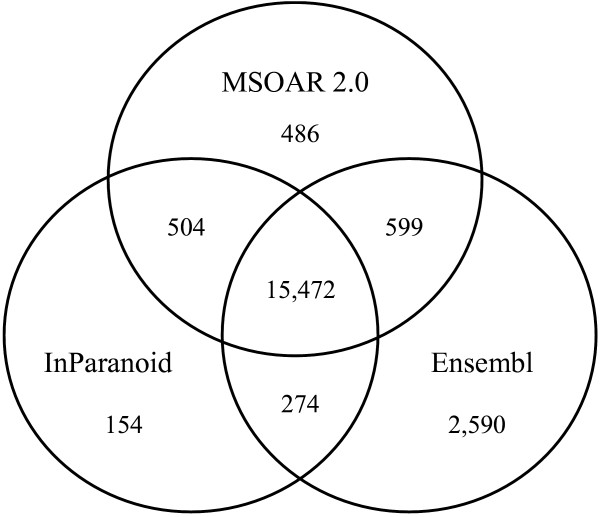
**Orthologs assigned between human and macaque**.

Since chimpanzee and macaque do not have official gene symbols, we only compare our assignment results with those of InParanoid and the Ensembl ortholog database. Figures 11 and 12 use Venn diagrams to show the commonality and difference among the ortholog pairs assigned by MSOAR 2.0, InParanoid, and the Ensembl ortholog database. We see that the three programs share more than 75% of the ortholog pairs. InParanoid outputs the least number of unique ortholog pairs while Ensembl has the most. More than 70% of the ortholog pairs unique to Ensembl are found to be inparalogs in TAGs (results not shown).

Table 4 shows the number of ortholog pairs output by MSOAR 2.0 that are shared by at least one of the other two programs. We observe that the closer the compared species is to human, the more support the result of MSOAR 2.0 receives from the other programs. For a pair of very closely related species, such as human and chimpanzee, the one-to-one ortholog pairs assigned by MSOAR 2.0 have nearly 99% support from at least one of the other two programs, which is consistent with our expectation, and confirms that MSOAR 2.0 is a highly accurate tool for one-to-one ortholog assignment between closely related species. (Note that the same claim can be made for the other two programs. In other words, ortholog assignment in general becomes easier for closely related species.)

Finally, we also observe that the number of inparalogs found in human by MSOAR 2.0 increases with the increase of evolutionary distance between human and the other species, as shown in Table 5. This is consistent with the definition of inparalogs.

**Table 4:** Support of the MSOAR 2.0 one-to-one ortholog pairs by the other two programs.

Support	human vs chimpanzee	human vs macaque	human vs mouse	human vs rat
By both programs	94.72%	90.69%	89.93%	87.71%
By at least one program	98.97%	97.15%	96.98%	96.48%

**Table 5:** Inparalogs found in human and the other species by MSOAR 2.0

Inparalogs found by MSOAR 2.0	human vs chimpanzee	human vs macaque	human vs mouse	human vs rat
Inparalogs in human	3,161	4,103	4,390	5,222
Inparalogs in the other species	569	3,962	6,454	6,548

## Conclusions

In this paper, we have incorporated a new gene duplication model, the tandem duplication model, into MSOAR, and developed an improved system of one-to-one ortholog assignment by combining gene phylogeny and genome rearrangement. By comparison with MSOAR, InParanoid, the Ensembl ortholog database, and MultiZ on both simulated and real data, we showed that MSOAR 2.0 achieves the best sensitivity while maintaining a high specificity. Although MSOAR 2.0 has a slightly lower specificity as compared to InParanoid on real data using gene symbols as the benchmark (*e.g.*, in the human-mouse comparison, 90.13% for InParanoid vs. 89.50% for MSOAR 2.0), it nevertheless identified several hundred of true one-to-one ortholog pairs that were missed by InParanoid. Because the majority of the "missed" one-to-one orthologs are not BBHs, which are what the InParanoid assignment is based on, MSOAR 2.0 clearly addresses a weakness of InParanoid. Moreover, MSOAR 2.0 shows a better specificity in the simulation tests. Note that MSOAR 2.0 also reconstructs the evolutionary history in terms of gene duplication and genome rearrangement, which could be of independent interest. Although Ensembl tends to assign a higher number of ortholog pairs than both InParanoid and MSOAR 2.0, MSOAR 2.0 outperforms it in terms of not only specificity but also sensitivity.

We evaluated the performance of the programs by computer simulations and gene symbols. However, simulations could be limited because the real evolutionary processes are much more complicated than what we can simulate. Furthermore, the use of gene symbols is not always feasible as many species do not have standard gene symbol assignment. We need to develop additional validation methods such as incorporating other available information, *e.g.*, gene functions. In addition, with the discovery of more mechanisms of gene evolution, new models of gene duplication (*e.g.*, segmental duplications) and genome operations (*e.g.*, *double cut and join *or DCJ), have been proposed. How to incorporate these new gene duplication models and operations into MSOAR 2.0 is our next challenge.

## Methods

### Gene Family Definition and Construction

A gene family is defined to be the set of genes that are all descended from a common ancestral gene [4,14]. Given two input genomes, our improved system starts by constructing gene families for all the genes on both genomes. We mix all protein sequences on both genomes and calculate the pairwise similarity scores by applying an all-versus-all BLASTp comparison [32]. By analyzing the results of BLASTp, we obtain a square similarity matrix, whose elements contain sequence similarity measurements for each pair of proteins in the dataset. Gene families can be calculated using the MCL (Markov clustering) algorithm [33] with default parameters.

Based on probability and graph flow theory, MCL simulates random walks on a graph using Markov matrices to determine the transition probabilities among the vertices of the graph. Unlike many other protein sequence clustering algorithms, MCL is able to deal with the presence of multi-domain proteins, promiscuous domains and fragmented proteins, making it one of the most widely used clustering algorithms in bioinformatics [33,34]. Some papers use MCL directly to identify ortholog groups such as OrthoMCL [12], while some others use TribeMCL (an extension of MCL) as a tool to find paralogs within a genome [23]. In our system, we apply MCL to cluster all homologous genes on both genomes (including all possible orthologs and paralogs) into gene families.

### DNA-based Gene Tree Reconstruction

For each gene family, we perform multiple sequence alignment using MAFFT [35,36] on the amino acid sequences of the genes and then calculate a DNA-based distance matrix. MAFFT is a rapid multiple sequence alignment tool based on fast Fourier transform, which has shown to be more accurate than other available tools including TCoffee [37] and ClustalW [38]. Moreover, MAFFT (with the fast mode) is able to align a large number (*e.g.*, several hundred) of sequences on a standard desktop PC in a few minutes.

Since DNA-based distance measure is shown to be more accurate than either protein-based distance or dS-based distance (*i.e.*, synonymous substitution rate) [5], we calculate the DNA-based distance for each gene family using the PHYLIP's DNADIST program [39] with the F84 nucleotide substitution model [40,41]. To obtain DNA sequence alignments, we reverse translate the amino acid sequence of each gene into its corresponding codon sequence using the program PAL2NAL [42] and the given genome sequences and then map the codon sequence onto its respective protein sequence alignment.

After getting the DNA-based distance matrix, we use the algorithm BIONJ [43] to reconstruct a gene tree for each family. Not only is BIONJ the best neighbor-joining algorithm for phylogenetic reconstruction, it was found to have a competitive (if not better) accuracy as many other popular phylogenetic reconstruction methods including PHYML [44], MrBayes [45] and PAML [46] in genome-wide reconstruction of gene trees according to a recent study [5]. Although maximum-likelihood methods are known to be more accurate than distance-based methods in general phylogenetic reconstruction, we chose a distance-based method here mostly because of its efficiency since MSOAR 2.0 has to deal with many large gene families consisting of very long sequences on real data. In order to produce a rooted gene tree for each family, we introduce before BIONJ is run an artificial outgroup gene whose distance to each of the other genes in the family is twice the maximum distance in the original distance matrix. This can be achieved by simply adding a new row and a new column in the original distance matrix. Running BIONJ on this expanded distance matrix is equivalent to mid-point rooting [47].

### Gene Duplication Dating on the Gene Tree

Once a gene tree is reconstructed, we need to label each of its internal nodes as either a duplication event or a speciation event. This process is a special case of the *gene duplication dating *problem, or the problem of reconciling a gene tree with a species tree. The phylogenetic tree reconciliation problem has been studied extensively in the literature, and many exact and heuristic algorithms have been proposed (see, *e.g.*, [48]). In our case, since only two species are involved, we propose a straightforward algorithm to date the duplication events in linear time.

To avoid postulating unnecessary gene losses, every internal node with descendant genes from the same species is labeled as a duplication event. Then, the lowest internal nodes with descendant genes from both species are labeled as speciation events. All ancestral nodes of the speciation nodes must be labeled as duplication events since there are only two species. An example of such a gene duplication dating algorithm is shown in Additional file 1, Figure S4.

### Identification of Inparalogs in TAGs

After dating duplications in a gene tree, we may deem each set of genes duplicated after the speciation event as a potential set of inparalogs (*e.g.*, *M*_*A *_and *M*_*B *_in Additional file 1, Figure S4). In order to confirm a potential set of inparalogs, we need to consider the positions of the genes on the concerned genome. If the potential inparalogs are adjacent to each other on the genome, *i.e.*, they appear in the same TAG, then we define them as inparalogs. For each such set of inparalogs, at most one gene can be included in a one-to-one ortholog pair. Since these genes appear in tandem, it would make no difference to the RD distance (which is the objective function of MSOAR) which of them is chosen to represent the set in the one-to-one ortholog pair. Thus, we will keep the gene that has the highest similarity score against any gene in the other genome and remove the other inparalogs in the same set so they will not be considered by MSOAR later on. If some potential inparalogs are separated by other genes on the genome, they will all be kept at this step and dealt with by MSOAR later on.

### Invocation of MSOAR and Post-Processing

After removing duplicated inparalogs in TAGs on each genome, MSOAR is now invoked on the remaining genes. To further improve the performance of MSOAR, we use a post-processing step. If we consider the positions of the one-to-one orthologs assigned by MSOAR on each genome, we find that in many cases a large consecutive block of assigned genes on one genome are orthologous to a consecutive block of assigned genes on the other genome with the same or reverse orientation. However, in some cases, there is a single unassigned gene (called a "gap") in each of the blocks forming an orthologous pair, and the gap appears at the same relative location in both blocks (see Figure 9 for an illustration). If the sequences of the two genes in the corresponding gaps are sufficiently similar (*e.g.*, at least one of the genes is the best hit of the other), then we deem the two genes as a one-to-one ortholog pair and add the pair to the output list.

### Generation of Simulated Data

The simulation is controlled by a set of 4 parameters (*k*, *p*, *α*, *β*) which are defined in the Simulation Results section. The simulation is performed as follows. We first generate an ancestral genome *G *with 100 genes, each of which is a random sequence of 3000 nucleotides (*i.e.*, 1000 codons). We randomly perform *k *duplications in *G *to obtain another genome *H*. Then, a speciation happens and the genome *H *evolves into two contemporary genomes *H*_1 _and *H*_2_. The evolution from genome *H *to each of the contemporary genomes involves *p *evolutionary events, including *p*·*α *duplications and *p *· (1 - *α*) reversals. Among all duplications, *β *of them are tandem (*i.e.*, we randomly choose a gene and insert its copy next to it) while the others are random (*i.e.*, we randomly choose a gene and insert its copy randomly into the genome). In order to simulate the sequence change of each gene along the evolutionary process, we set a constant mutation rate *μ *= 1% to allow each gene on the genomes to have up to 3000 *μ *= 30 random mutations of its nucleotides between every two evolutionary events (*i.e.*, 15 random nucleotide mutations would be performed on the average).

### Real Data

Protein sequences, transcripts, and gene locations for all five species, human (*Homo sapiens*), mouse (*Mus musculus*), rat (*Rattus norvegicus*), chimpanzee (*Pan troglodytes*) and macaque (*Macaca mulatta*) (version 52, December 2008) were downloaded from Ensembl genome browser http://www.ensembl.org/. Genes annotated as novel, supercontig, or mitochondrial are removed, and only protein-coding genes with known chromosome locations are kept. For genes with alternative splicing variants, we use their longest transcripts. Similar methods have been used in the previous studies [23,49]. After such pre-processing, we obtained 21,164, 23,228, 22,490, 18,572, and 21,023 genes for human, mouse, rat, chimpanzee, and macaque, respectively.

## Authors' contributions

GS designed and implemented the improved system MSOAR 2.0. LZ provided insight on gene duplication models. GS and TJ drafted the manuscript, and TJ supervised the project. All authors read and approved the manuscript.

## Supplementary Material

Additional file 1 contains four supplementary figures which may help explain some fundamental concepts in gene duplication, orthology and paralogy Click here for file

Additional file 2 contains the software MSOAR 2.0. Click here for file
