# Supplementary material for: MSOAR 2.0: Incorporating tandem duplications into ortholog assignment based on genome rearrangement
Source: BMC Bioinformatics. 2010 Jan 6;11:10. doi: 10.1186/1471-2105-11-10 (PMC2821317; doi:10.1186/1471-2105-11-10)
Supplement: Additional file 1 — contains four supplementary figures which may help explain some fundamental concepts in gene duplication, orthology and paralogy [file 1471-2105-11-10-S1.PDF]

Supplementary Figures

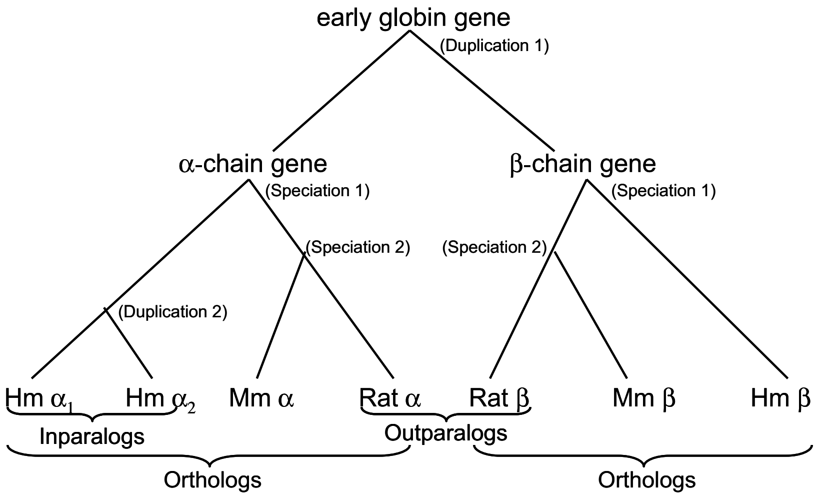

Figure S1: An illustration of orthology and paralogy relationships. The α globin genes on different species are orthologous to each other, and they form an ortholog group. So do the β globin genes. The rat α and β globin genes are outparalogs with respect to the speciation of mouse and rat (*i.e.*, speciation 2) as well as with respect to speciation 1. The human α_1 and α_2 globin genes are inparalogs with respect to the speciation of human and rat (*i.e.*, speciation 1).

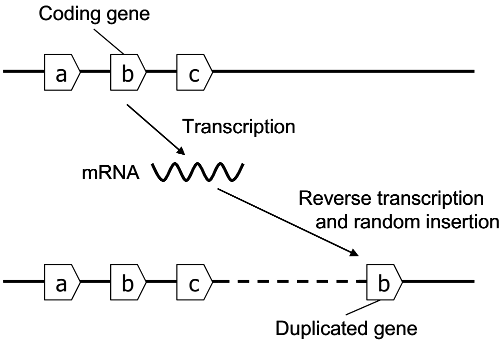

Figure S2: Retrotransposition.

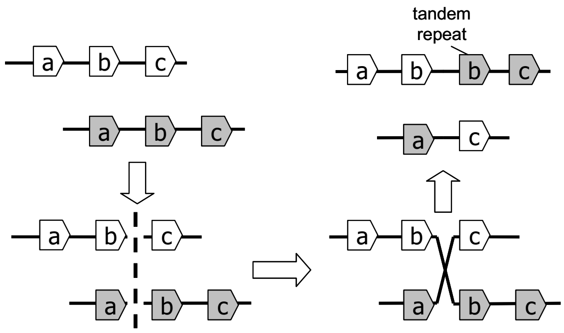

Figure S3: Unequal crossover.

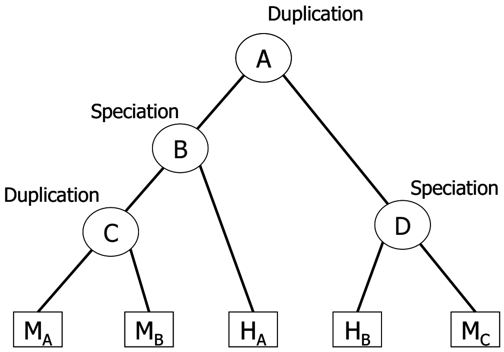

Figure S4: An example of the gene duplication dating algorithm. Node C is a duplication event since M_A and M_B are both from the same species. Node B and D correspond to speciation events since they have descendant genes from two species. Node A is a duplication event since it is the ancestral node of speciation nodes B and D.
