# Supplementary material for: MSOAR 2.0: Incorporating tandem duplications into ortholog assignment based on genome rearrangement
Source: BMC Bioinformatics. 2010 Jan 6;11:10. doi: 10.1186/1471-2105-11-10 (PMC2821317; doi:10.1186/1471-2105-11-10)
Supplement: Additional file 2 — contains the software MSOAR 2.0. [file 1471-2105-11-10-S2.GZ › MSOAR2.0/Programs/mcl-06-058/src/alien/oxygen/doc/mclblastline.html]

The mclblastline manual


27 Feb 2006   
**mclblastline**
1.006, 06-058

## NAME

mclblastline - a pipeline for clustering from BLAST files.

## SYNOPSIS

**mclblastline** file-name

**mclblastline** [deblast options] [pipeline options] file-name

**NOTE**  
**mcl** has acquired the ability to proceed from label input
as produced by **mcxdeblast**. This enables a very lightweight
mechanism of generating clusterings from BLAST files.
You might want to use this mechanism,
documented in the mcl manual.

**NOTE**  
mclblastline used to require (given default parameters)
the presence of the **zoem** macro processor to produce detailed output.
This is no longer the case. By default mclblastline now creates
a line-based tab-separated dump file.
Zoem *will* be invoked when the --fmt-fancy option is supplied.
In that case, zoem obviously has to be installed.

## DESCRIPTION

**mclblastline** wraps around **mclpipeline**. It supplies the
**--parser**=*app*
and **--parser-tag**=*str* options, setting them respectively
to *mcxdeblast* and *blast*. This tells
mclpipeline to use
mcxdeblast as the parse script in its pipeline.
The significance of
the *blast* tag is that any mcxdeblast option can be passed through
mclblastline and mclpipeline by inserting this tag into the option.
For example, mcxdeblast accepts the **--score**=*x* option.
When using mclblastline, you specify it as **--blast-score**=*x*.
There are two exceptions to this rule, namely the
**--xi-dat**=*str* and **--xo-dat**=*str* options. Refer to
the mclpipeline manual for more information.

Additionally, all mclpipeline options are acceptable to mcxdeblast as
well. The **--whatif** is useful for getting a feel for the pipeline.
The **--mcl-I**=*f* inflation option and **--mcl-scheme**=*i* scheme
index options are your basic means for respectively manipulating cluster
granularity and allocating resources. Read the mcl manual entries for
a description of the corresponding **-I** and **-scheme** mcl
options.

The best advice is to glance over the mcxdeblast and mclpipeline
options in order to get a feeling for which of those may come in handy for
you. Then start experimenting. Use the **--whatif** option, it will
tell you what would happen without actually doing it.

## OPTIONS

All mcxdeblast and mclpipeline options.
mcxdeblast options must be passed using the mechanism described above.

## EXAMPLES

```
   mclblastline --blast-score=b
      --blast-sort=a --blast-bcut=5 --mcl-I=2.5 myblastfile
```

This will use bit scores, sort cluster indices such that the corresponding
labels are ordered alphabetically, ignore bit scores not exceeding 5, and
use inflation value 2.5. In this case, the output clustering will be in the
file named myblastfile.I25s2 (I25 identifying the inflation value and s2
identifying the resource scheme) and the formatted output will be in the
file myblastfile.I25s2.fmt.

```
   mclblastline --prepare-mcl myblastfile
   mclblastline --start-mcl --mcl-I=1.2 myblastfile
   mclblastline --start-mcl --mcl-I=1.6 myblastfile
   mclblastline --start-mcl --mcl-I=2.0 myblastfile
   mclblastline --start-mcl --mcl-I=2.4 myblastfile
   mclblastline --start-mcl --mcl-I=2.8 myblastfile
   etc ..
```

The first run prepares an input matrix to be read by **mcl**.
In this case its file will be named myblastfile.sym.
The subsequent runs use this matrix.
**CAVEAT** there are some options that you need to repeat
when executing such a resumed run. They are clearly marked
in the mclpipeline manual - namely those options
that affect names of (intermediate) files. Most importantly,
this concerns the mclpipeline options that have prefix
**--xo** or **--xi**. For example,

```
   mclblastline --prepare-mcl --blast-score=b --xo-dat=b myblastfile
   mclblastline --start-mcl --xo-dat=b --mcl-I=1.2 myblastfile
   mclblastline --start-mcl --xo-dat=b --mcl-I=1.6 myblastfile
   mclblastline --start-mcl --xo-dat=b --mcl-I=2.0 myblastfile
   mclblastline --start-mcl --xo-dat=b --mcl-I=2.4 myblastfile
   mclblastline --start-mcl --xo-dat=b --mcl-I=2.8 myblastfile
   etc ..
```

In this case, the matrix file will be named myblastfile.b.sym,
and the **--xo-dat** options must be repeated in all runs
so that the pipeline reconstructs the correct file name(s).

## AUTHOR

Stijn van Dongen

## SEE ALSO

mcxdeblast, mclpipeline, mcxassemble.
