# Supplementary material for: MSOAR 2.0: Incorporating tandem duplications into ortholog assignment based on genome rearrangement
Source: BMC Bioinformatics. 2010 Jan 6;11:10. doi: 10.1186/1471-2105-11-10 (PMC2821317; doi:10.1186/1471-2105-11-10)
Supplement: Additional file 2 — contains the software MSOAR 2.0. [file 1471-2105-11-10-S2.GZ › MSOAR2.0/Programs/mcl-06-058/doc/clmresidue.html]

The clmresidue manual


27 Feb 2006   
**clmresidue**
1.006, 06-058

|  |  |  |
| --- | --- | --- |
| 1. |  | NAME |
| 2. |  | SYNOPSIS |
| 3. |  | DESCRIPTION |
| 4. |  | OPTIONS |
| 5. |  | AUTHOR |
| 6. |  | SEE ALSO |

## NAME

clmresidue - extend clustering of subgraph to clustering of graph.

## SYNOPSIS

**clmresidue**
**-icl** fname (*input clustering*)
**-imx** fname (*input graph*)
**[-rpm** fname (*residue projection matrix*)**]**
**[--adapt** (*allow domain mismatch*)**]**
**[-mvp** fname (*majority vote projection (clustering)*)**]**


## DESCRIPTION

The cluster file presumably contains a clustering of a subgraph of the
graph G contained by the matrix file, implying that the row domain of
that clustering is a subset of the node domain of the graph. clmresidue will
compute a simple upwards projection of that clustering, resulting in a
clustering of G.

## OPTIONS

|  |  |  |
| --- | --- | --- |
| **-rpm** fname (*residue projection matrix*) | | |
|  |  | The name for the file in which the residue projection matrix is stored. It contains for each node and for each cluster in the input clustering the combined weight of the (edges to the) neighbours of that node in that cluster. The default output file name is out.rpm. |
|  |  |  |
| **-mvp** fname (*majority vote projection (clustering)*) | | |
|  |  | The name for the file in which the majority vote projection clustering is stored. Aka the majority vote projection clustering storage file name. This embodies a very crude approach to distributing the missing nodes in the input clustering onto that same cluster. For each node, the cluster is taken for which the residue projection is the largest (see above). The residue (i.e. the set of missing nodes) is initially viewed as a cluster; the set of nodes for which the projection is largest for the residue itself will form a new cluster, if it is not empty. The default output file name is out.mvp. |
|  |  |  |
| **--adapt** (*allow domain mismatch*) | | |
|  |  | Allow the clustering domain to contain nodes not present in the graph domain. **clmresidue** will take the appropriate subclustering. |

## AUTHOR

Stijn van Dongen.

## SEE ALSO

mclfamily for an overview of all the documentation
and the utilities in the mcl family.
