# Supplementary material for: MSOAR 2.0: Incorporating tandem duplications into ortholog assignment based on genome rearrangement
Source: BMC Bioinformatics. 2010 Jan 6;11:10. doi: 10.1186/1471-2105-11-10 (PMC2821317; doi:10.1186/1471-2105-11-10)
Supplement: Additional file 2 — contains the software MSOAR 2.0. [file 1471-2105-11-10-S2.GZ › MSOAR2.0/Programs/mcl-06-058/doc/clminfo.html]

The clminfo manual


27 Feb 2006   
**clminfo**
1.006, 06-058

|  |  |  |
| --- | --- | --- |
| 1. |  | NAME |
| 2. |  | SYNOPSIS |
| 3. |  | DESCRIPTION |
| 4. |  | OPTIONS |
| 5. |  | AUTHOR |
| 6. |  | SEE ALSO |
| 7. |  | REFERENCES |

## NAME

clminfo - Compute performance measures for graphs and clusterings.

## SYNOPSIS

**clminfo** [options] <graph file> <cluster file> <cluster file>\*

**clminfo**
**[--adapt** (*allow domain mismatch*)**]**
**[-pi** f (*apply inflation beforehand*)**]**
**[--append-pf** (*append performance measures*)**]**
**[--append-gr** (*append granularity measures*)**]**
**[-tag** <str> (*write tag=<str> in performance section*)**]**
<graph file> <cluster file> <cluster file>\*

## DESCRIPTION

**clminfo** computes several numbers indicative for the efficiency with
with a clustering captures the edge mass of a given graph.
Use it in conjunction with **clmdist** to determine which clusterings
you accept. See the EXAMPLES section in clmdist
for an example of **clmdist** and **clminfo** (and **clmmeet**) usage.
Output can be generated for multiple clusterings at the same time.

The first number is called the **efficiency** and is described in [1] (see
the REFERENCES section). It tries to balance the dual aims of
capturing a lot of edges or edge weights and keeping the cluster footprint
or area fraction small. The efficiency number has several appealing
mathematical properties, cf. [1]. It is related to, but not derivable from,
the second and third numbers, the *mass fraction* and the
*area fraction*.

The second number is the mass fraction, which is defined as follows.
Let **e** be an edge of the graph. The clustering *captures* **e**
if the two nodes associated with **e** are in the same cluster.
Now the mass fraction is the joint weight of all captured edges divided
by the joint weight of all edges in the input graph.

The third number is the area fraction, which is roughly the sum of the
squares of all cluster sizes for all clusters in the clustering, divided by
the square of the number of nodes in the graph. It says *roughly*,
because the actual formula uses the quantity **N**\*(**N-1**) wherever it
says square (of **N**) above. A low/high area fraction indicates a
fine-grained/coarse clustering.

The fourth number is the cluster link weight, which is the average
edge weight over all edges captured by the clustering.

The fifth number is the graph link weight, which is the average weight
over all edges in the graph.

## OPTIONS

|  |  |  |
| --- | --- | --- |
| **--adapt** (*allow domain mismatch*) | | |
|  |  | The **--adapt** option allows the graph domain and cluster domain not to match. **clmdist** will take the appropriate subclustering and subgraph as it goes along. |
|  |  |  |
| **-pi** f (*apply inflation beforehand*) | | |
|  |  | Apply inflation to the graph matrix and compute the performance measures for the result. |
|  |  |  |
| **--append-pf** (*append performance measures*) | | |
|  |  | **clminfo** will reread each cluster file and append the computed performance measures to it. |
|  |  |  |
| **--append-gr** (*append granularity measures*) | | |
|  |  | **clminfo** will reread each cluster file and append the computed granularity measures to it. |
|  |  |  |
| **-tag** <str> (*write tag=<str> in performance section*) | | |
|  |  | When appending, **clminfo** will include tag=<str> in the performance section. This can be useful e.g. to indicate the pre-inflation value corresponding with the section. |

## AUTHOR

Stijn van Dongen.

## SEE ALSO

mclfamily for an overview of all the documentation
and the utilities in the mcl family.

## REFERENCES

[1] Stijn van Dongen. *Performance criteria for graph clustering and Markov
cluster experiments*. Technical Report INS-R0012, National Research
Institute for Mathematics and Computer Science in the Netherlands,
Amsterdam, May 2000.  
http://www.cwi.nl/ftp/CWIreports/INS/INS-R0012.ps.Z
