# Supplementary material for: MSOAR 2.0: Incorporating tandem duplications into ortholog assignment based on genome rearrangement
Source: BMC Bioinformatics. 2010 Jan 6;11:10. doi: 10.1186/1471-2105-11-10 (PMC2821317; doi:10.1186/1471-2105-11-10)
Supplement: Additional file 2 — contains the software MSOAR 2.0. [file 1471-2105-11-10-S2.GZ › MSOAR2.0/Programs/mcl-06-058/doc/clmmate.html]

The clmmate manual


27 Feb 2006   
**clmmate**
1.006, 06-058

|  |  |  |
| --- | --- | --- |
| 1. |  | NAME |
| 2. |  | SYNOPSIS |
| 3. |  | DESCRIPTION |
| 4. |  | AUTHOR |
| 5. |  | SEE ALSO |

## NAME

clmmate - compute best matches between two clusterings

## SYNOPSIS

**clmmate** [-l] [-o fname] <clfile1> <clfile2>

## DESCRIPTION

**clmmate** computes for each cluster X in clfile1 all clusters
Y in clfile2 that have non-empty intersection and outputs
a line with the data points listed below.

```
   overlap(X,Y)               # 2 * size(meet(X,Y)) / (size(X)+size(Y))
   index(X)                   # name of cluster
   index(Y)                   # name of cluster
   size(meet(X,Y))
   size(X-Y)                  # size of left difference
   size(Y-X)                  # size of right difference
   size(X)
   size(Y)
   projection(X, clfile2)     # see below
   projection(Y, clfile1)     # see below
```

Use the **-l** option to include a legend heading the output.

The projected size of a cluster X relative to a clustering K is
simply the sum of all the nodes shared between any cluster Y in K
and X, duplications allowed. For example, the projected size of
(0,1) relative to {(0,2,4), (1,4,9), (1,3,5)} equals 3.

The overlap between X and Y is exactly
1.0 if the two clusters are identical, and for nearly identical
clusterings the score will be close to 1.0.

All of this information can also be obtained from the
contingency matrix defined for two clusterings.
The [i,j] row-column entry in a contigency matrix between
to clusterings gives the number of entries in the intersection
between cluster i and cluster j from the respective
clusterings. The other information is implicitly present;
the total number of nodes in clusters i and j
for example can be obtained as the sum of entries in row i
and column j respectively, and the difference counts
can then be obtained by substracting the intersection count.
The contingency matrix can easily be computed using **mcx**;
e.g.

```
mcx /clfile2 lm /clfile1 lm tp mul /ting wm
```

will create the contingency matrix in mcl matrix format
in the file ting, where columns range over the clusters
in clfile1.

The output can be put to good use by sorting it numerically on
that first score field. It is advisable to use a stable sort routine
(use the **-s** option for UNIX sort)
From this information one can quickly extract the closest
clusters between two clusterings.

## AUTHOR

Stijn van Dongen.

## SEE ALSO

mclfamily for an overview of all the documentation
and the utilities in the mcl family.
