# Supplementary material for: MSOAR 2.0: Incorporating tandem duplications into ortholog assignment based on genome rearrangement
Source: BMC Bioinformatics. 2010 Jan 6;11:10. doi: 10.1186/1471-2105-11-10 (PMC2821317; doi:10.1186/1471-2105-11-10)
Supplement: Additional file 2 — contains the software MSOAR 2.0. [file 1471-2105-11-10-S2.GZ › MSOAR2.0/Programs/mcl-06-058/doc/clmclose.html]

The clmclose manual


27 Feb 2006   
**clmclose**
1.006, 06-058

|  |  |  |
| --- | --- | --- |
| 1. |  | NAME |
| 2. |  | SYNOPSIS |
| 3. |  | DESCRIPTION |
| 4. |  | AUTHOR |
| 5. |  | SEE ALSO |

## NAME

clmclose - Fetch connected components from graphs or subgraphs

## SYNOPSIS

**clmclose** -imx <fname> [options]

**clmclose**
**-imx** fname (*input file*)
**-dom** fname (*input domain/cluster file*)
**[-o** fname (*output file*)**]**
**[-cc** (*output connected components*)**]**
**[-h** (*list help*)**]**
**[--apropos** (*list synopsis of all options*)**]**


## DESCRIPTION

Currently, **-cc** is the only mode provided, implying
its use is required.

Use **clmclose** to fetch the connected components from a graph.
They will be returned as a clustering file. Use this file
in conjunction with **mcxsubs** and the graph file itself to
retrieve individual subgraphs corresponding to connected components.

If the **-dom** *fname* option is used, clmclose will
for each of the domains in file *fname* retrieve
the associated subgraph from the input graph, decompose it into
its connected components, and write each connected components
as a separate domain/cluster in the output file.

## AUTHOR

Stijn van Dongen.

## SEE ALSO

mclfamily for an overview of all the documentation
and the utilities in the mcl family.
