# Supplementary material for: MSOAR 2.0: Incorporating tandem duplications into ortholog assignment based on genome rearrangement
Source: BMC Bioinformatics. 2010 Jan 6;11:10. doi: 10.1186/1471-2105-11-10 (PMC2821317; doi:10.1186/1471-2105-11-10)
Supplement: Additional file 2 — contains the software MSOAR 2.0. [file 1471-2105-11-10-S2.GZ › MSOAR2.0/Programs/mcl-06-058/doc/clmdist.html]

The clmdist manual


27 Feb 2006   
**clmdist**
1.006, 06-058

|  |  |  |
| --- | --- | --- |
| 1. |  | NAME |
| 2. |  | SYNOPSIS |
| 3. |  | DESCRIPTION |
| 4. |  | SPLIT/JOIN DISTANCE |
| 5. |  | EXAMPLES |
| 6. |  | AUTHOR |
| 7. |  | SEE ALSO |
| 8. |  | REFERENCES |

## NAME

clmdist - compute the distance between two or more partitions (clusterings).

Distance can be any of
*split/join distance*, *variance of information*,
*Mirkin metric*, or *scatter distance*.

## SYNOPSIS

**clmdist** [options] <file name> <file name>+

**clmdist**
**[--adapt** (*allow domain mismatch*)**]**
**[-mode** <sj|vi|mk|sc> (*distance type*)**]**
**[-digits** k (*output decimals*)**]**
**[-width** k (*tab width*)**]**
**[--noindex** (*omit indices*)**]**
**[-parts** <l|d|u>+ (*array parts to print*)**]**
<file name> <file name>+

## DESCRIPTION

**clmdist** computes distances between clusterings. It can compute
the *split/join distance* (described below),
the *variance of information measure*,
the *Mirkin metric*,
and the *scatter* distance.
It can compute the chosen distance for all pairs of distances in a set of
clusterings. Clusterings must be in the mcl matrix format (Cf. mcxio),
and are supplied on the command line as the names of the files in which they
are stored.

Currently, **clmdist** cannot compute different distance types simultaneously,
with one exception. The scatter distance *can* be computed simultaneously
with any of the other three distances.

|  |  |  |
| --- | --- | --- |
| **--adapt** (*allow domain mismatch*) | | |
|  |  | Allow a pair of clusterings to pertain to different domains. **clmdist** will project both clusterings onto the meet of the domains. |
|  |  |  |
| **-mode** <sj\|vi\|mk\|sc> (*distance type*) | | |
|  |  | Use **sj** for the *split/join distance* (described below), **vi** for the *variance of information measure*, **mk** for the *Mirkin metric*, and **sc** for the *scatter distance*. The scatter distance *can* be computed simultaneously with any of the other three distances. This is achieved e.g. by supplying **-mode** **sj** **-mode** **sc**. |
|  |  |  |
| **-digits** k (*output decimals*) | | |
|  |  | The number of decimals printed when using the variance of information measure. |
|  |  |  |
| **-width** k (*tab width*) | | |
|  |  | The tabbing width in the output table. |
|  |  |  |
| **--noindex** (*omit indices*) | | |
|  |  | Do not print indices around the table. |
|  |  |  |
| **-parts** <l\|d\|u>+ (*array parts to print*) | | |
|  |  | Use **l** to print the lower diagonal part of the table, use **d** to print the (boring) diagonal part of the table (it's full of zeroes), use **u** to print the upper diagonal part of the table. You may use any combination of these. |

## SPLIT/JOIN DISTANCE

For each pair of clusterings **C1**, **C2**, two numbers are given,
say **d1** and **d2**. Then **d1** + **d2** equals the number
of nodes that have to be exchanged in order to transform any of the two
clusterings into the other, and you can think of (**d1**+**d2**)/**2N**
as the percentage that the two clusterings differ. The split/join
distance has a linearity property with respect to the meet of **C1** and
**C2**, see below.

The split/join distance **sjd** is very handy in computing the consistency of
two or more clusterings of the same graph, or comparing clusterings made
with different resource (but otherwise identical) parameters. The latter is
for finding out whether you can settle for cheaper mcl settings, or whether
you need to switch to more expensive settings. The former is for finding out
whether clusterings are identical, conflicting, or whether one is (almost) a
subclustering of the other - mostly for comparing a set of clusterings of
different granularity, made by letting the mcl parameter **-I** vary.
The EXAMPLES section contains examples of all these **clmdist** uses,
and the use of clminfo and clmmeet is also discussed there.

**sjd** is a metric distance on the space of partitions of
a set of a given fixed cardinality. It has the following linearity
property. Let **P1** and **P2** be partitions, then

**sjd**(**P1**, **P2**) = **sjd**(**P1**, **D**) + **sjd**(**P2**, **D**)

where **D** (for Dutch Doorsnede)
is the intersection of **P1** and **P2**, i.e. the unique clustering
that is both a subclustering of **P1** and **P2** *and* a superclustering of
all other subclusterings of **P1** and **P2**. Sloppily worded, **D** is the largest
subclustering of both **P1** and **P2**. See the REFERENCES section for
a pointer to the technical report in which **sjd** was first defined (and in
which the non-trivial triangle inequality is proven).

Because it is useful to know whether one partition (or clustering)
is almost a subclustering of the other, **clmdist** returns the
two constituents **sjd**(**P1**,**D**) and **sjd**(**P2**,**D**).

The output of **clmdist** is slightly different depending on whether two or
more than two clusterings are given. In the first case, one pair of numbers
is returned (the **sjd** constituents). In the latter case, an array is
returned for which the upper triangular part contains all possible
distances. The array is extended with the (file) names of the clusterings.

Let **P1** and **P2** be two clusterings of a graph of cardinality **N**,
and suppose **clmdist** returns the integers **d1** and **d2**. You can think of
**100 \* (d1 + d2) / N** as the percentage that **P1** and **P2** differ.
This interpretation is in fact slightly conservative.
The numerator is the number of nodes that need to be exchanged in order to
transform one into the other. This number may grow as large as
**2\*N - 2\*sqrt(N)**, so it would be justified to take 50 as a scaling
factor rather than 100.

For example, if **A** and **B** are both clusterings of a graph
on a set of 9058 nodes and **clmdist** returns [38, 2096], this conveys
that **A** is almost a subclustering of **B** (by splitting 38 nodes
in **A** we obtain a clustering **D** that is a subclustering of **B**),
and that **B** is much less granular than **A**. The latter is
because we can obtain **B** from **D** by *joining* 2096 nodes
in some way.

## EXAMPLES

The following is an example of several mcl validation tools
applied to a set of clusterings on a protein graph of 9058 nodes.
In the first experiment, six
different clusterings were generated for different values of the inflation
parameter, which was respectively set to 1.2, 1.6, 2.0, 2.4, 2.8, and 3.2.
It should be noted that protein graphs seem somewhat special in that an
inflation parameter setting as low as 1.2 still produces a very acceptable
clustering. The six clusterings are scrutinized using **clmdist**,
**clminfo**, and **clmmeet**.
In the second experiment, four different clusterings were generated
with identical flow (i.e. inflation) parameter, but
with different resource parameters. **clmdist** is used to choose
a sufficient resource level.

High **-P/-S/-R** values make **mcl** more accurate but also
more time and memory consuming. Run **mcl** with different settings for these
parameters, holding other parameters fixed. If the expensive and supposedly
more accurate clusterings are very similar to the clusterings resulting from
cheaper settings, the cheaper setting is sufficient. If the distances
between cheaper clusterings and more expensive clusterings are large, this
is an indication that you need the expensive settings. In that case, you may
want to increase the **-P/-S/-R** parameters (or simply the
**-scheme** parameter) until associated
clusterings at nearby resource levels are very similar.

In this particular example, the validation tools do not reveal that one
clustering in particular can be chosen as 'best', because all clusterings
seem at least acceptable. They do aid however in showing the relative
merits of each clusterings. The most important issue in this respect is
cluster granularity. The table below shows the output of clminfo.

```
     Efficiency  Mass frac  Area frac  Cl weight  Mx link weight
1.2   0.42364     0.98690    0.02616    52.06002    50.82800
1.6   0.58297     0.95441    0.01353    55.40282    50.82800
2.0   0.63279     0.92386    0.01171    58.09409    50.82800
2.4   0.65532     0.90702    0.01091    59.58283    50.82800
2.8   0.66854     0.84954    0.00940    63.19183    50.82800
3.2   0.67674     0.82275    0.00845    66.10831    50.82800
```

This data shows that there is exceptionally strong cluster structure present
in the input graph. The 1.2 clustering captures almost all edge mass using
only 2.5 percent of 'area'. The 3.2 clustering still captures 82 percent of
the mass using less than 1 percent of area. We continue with looking at the
mutual consistency of the six clusterings. Below is a table that shows all
pairwise distances between the clusterings.

```
    |   1.6  |   2.0  |   2.4  |   2.8  |   3.2  |   3.6
-----------------------------------------------------------.
1.2 |2096,38 |2728,41 |3045,48 |3404,45 |3621,43 |3800, 42 |
-----------------------------------------------------------|
1.6 |        | 797,72 |1204,76 |1638,78 |1919,70 |2167, 69 |
-----------------------------------------------------------|
2.0 |        |        | 477,68 | 936,78 |1235,85 |1504, 88 |
-----------------------------------------------------------|
2.4 |        |        |        | 498,64 | 836,91 |1124,103 |
-----------------------------------------------------------|
2.8 |        |        |        |        | 384,95 | 688,119 |
-----------------------------------------------------------|
3.2 |        |        |        |        |        | 350,110 |
-----------------------------------------------------------.
```

The table shows that the different clusterings are pretty consistent with
each other, because for two different clusterings it is generally true that
one is almost a subclustering of the other. The interpretation for the
distance between the 1.6 and the 3.2 clustering for example, is that by
rearranging 43 nodes in the 3.2 clustering, we obtain a subclustering of the
1.6 clustering. The table shows that for any pair of clusterings, at most
119 entries need to be rearranged in order to make one a subclustering of
the other.

The overall consistency becomes all the more clear by looking at the meet of
all the clusterings:

```
clmmeet -o meet out12 out16 out20 out24 out28 out32
clmdist meet out12 out16 out20 out24 out28 out32
```

results in the following distances between the respective clusterings
and their meet.

```
    |   1.2  |    1.6 |  2.0   |   2.4  |  2.8   |  3.2    |  
-------------- --------------------------------------------.
meet|  0,3663|  0,1972| 0,1321 |  0,958 | 0,559  | 0,283   |
-------------- --------------------------------------------.
```

This shows that by rearranging only 283 nodes in the 3.2 clustering,
one obtains a subclustering of all other clusterings.

In the last experiment, **mcl** was run with inflation parameter 1.4,
for each of the four different preset pruning schemes k=1,2,3,4.
The **clmdist** distances between the different clusterings
are shown below.

```
    |  k=2   |   k=3  |   k=4  |
-------------------------------.
k=1 |  17,17 |  16,16 |  16,16 |
-------------------------------.
k=2 |        |   3,3  |   5,5  |
-------------------------------.
k=3 |        |        |   4,4  |
-------------------------------.
```

This example is a little boring in that the cheapest scheme seems adequate.
If anything, the gaps between the k=1 scheme and the rest are a little
larger than the three gaps between the k=2, k=3, and k=4
clusterings. Had all distances been much larger, then such an observation
would be reason to choose the k=2 setting.

Note that you need not feel uncomfortable with the clusterings
still being different at high resource levels, if ever so slightly.
In all likelihood, there are anyway nodes which are not in any core of
attraction, and that are on the boundary between two or more clusterings.
They may go one way or another, and these are the nodes which
will go different ways even at high resource levels.
Such nodes may be stable in clusterings obtained for lower inflation
values (i.e. coarser clusterings), in which the different clusters
to which they are attracted are merged.

## AUTHOR

Stijn van Dongen.

## SEE ALSO

mclfamily for an overview of all the documentation
and the utilities in the mcl family.

## REFERENCES

Stijn van Dongen. *Performance criteria for graph clustering and Markov
cluster experiments*. Technical Report INS-R0012, National Research
Institute for Mathematics and Computer Science in the Netherlands,
Amsterdam, May 2000.  
http://www.cwi.nl/ftp/CWIreports/INS/INS-R0012.ps.Z

Marina Meila. *Comparing Clusterings - An Axiomatic View*.
In *Proceedings of the 22nd International Conference on Machine Learning*,
Bonn, Germany, 2005.

Marina Meila. *Comparing Clusterings*,
UW Statistics Technical Report 418.  
http://www.stat.washington.edu/www/research/reports/2002/tr418.ps
