# Supplementary material for: MSOAR 2.0: Incorporating tandem duplications into ortholog assignment based on genome rearrangement
Source: BMC Bioinformatics. 2010 Jan 6;11:10. doi: 10.1186/1471-2105-11-10 (PMC2821317; doi:10.1186/1471-2105-11-10)
Supplement: Additional file 2 — contains the software MSOAR 2.0. [file 1471-2105-11-10-S2.GZ › MSOAR2.0/Programs/mcl-06-058/doc/mcx.html]

The mcx manual


27 Feb 2006   
**mcx**
1.006, 06-058

|  |  |  |
| --- | --- | --- |
| 1. |  | NAME |
| 2. |  | SYNOPSIS |
| 3. |  | DESCRIPTION |
| 4. |  | OPERATORS |
| 5. |  | INDEX |
| 6. |  | AUTHOR |
| 7. |  | SEE ALSO |

## NAME

mcx - a stack language interpreter for interaction with the **mcl** libraries.

## SYNOPSIS

**mcx** (enter interactive mode)

**mcx** <mcx expression>

## DESCRIPTION

**mcx** can be used both from the command line and interactively, and supports
a rich set of operations such as transposition, scaling, column scaling,
multiplication, Hadamard powers and products, et cetera. It has variables,
control primitives, and stack manipulation primitives.
The general aim is to support basic number and matrix arithmetic, as well as
graph, set, and clustering operations. The language will be kept simple,
and should serve only as a means of making the **mcl** framework a pleasant one
for working with graphs, matrices, clusterings, and sets.
The language is typed, and currently has the types *integer, real,
matrix, string, and block*.
Blocks are used both in control statements such as **ifelse** and
**while**, and for defining compound statements resembling procedures or
functions.
Some of **mcx**'s primitives are overloaded. The following is a very simple
example of implementing and using **mcl** in this language.

```
 2.0 .i def                   # define inflation value.
 /small lm                    # load matrix in file 'small'.
 dim id add                   # add identity matrix.
 st .x def                    # make stochastic, bind to x.
 { xpn .i infl vm } .mcl def  # define one mcl iteration.
 20 .x .mcl repeat            # iterate 20 times
 imac                         # interpret matrix as clustering.
 vm                           # view matrix (clustering).
```

Somewhat misleadingly, '#' is not recognized as a comment
symbol by **mcx**, so don't use it (yet).

**mcx** has quite a lot of primitives, and they are not all explained in here.
However, **mcx** has several help facilities. **mcx**'s munge modes (command
line, interactive, and file) are discussed first, followed by some syntactic
and semantic rules.
Several groups of related primitives are then discussed. These are the help
primitives **help**, **grep**, **ops**, and others,
the binding primitives **def**, **free**, and **unlink**,
the control primitives **ifelse**, **do**, **repeat**, and **while**,
and the stack manipulation primitives **pop**, **exch**, **dup**,
**mdup**, **copy**, **roll**, **clear**.
And for the record, **mcx** has **lt**, **lq**, **eq**, **gq**, and
**gt**.
In the grandiloquently named INDEX section, you find a list
of all current **mcx** primitives.

First, there is no difference as far as **mcx** is concerned whether
you give it a bunch of operators and operands directly from the command
line, or whether you do it in interactive mode. Interactive mode is
line-based; each time you type a carriage return, **mcx** starts interpreting
the line you typed. Newlines can be escaped by preceding them with a
backslash;
**mcx** will delay interpretation and keep building up your expression.
Command line mode is the same as entering interactive mode and entering a
single line. For non-batch processing, interactive mode is the safest,
because **mcx** will simply refuse to carry out impossible operations,
whereas in command line mode it will exit.

In interactive mode, **mcx** will by default list the stack after each line you
type. This is verbosity level 1. There are four verbosity levels, namely 0,
1, 2, and 3 (in increasingly verbose order). Set the level with the **vb**
primitive. Verbosity control is still a bit crude, this is one of the areas
in which **mcx** will be improved.

There is another **mcx** munge mode which is entered when reading files
with the **lf** (load file) primitive. This mode is not line-based,
the whole file is interpreted in one go - there is little
difference with line-based mode. Beware though, it is currently
not possible (nor necessary) to escape newlines in files.

Currently, **mcx** parses lines of text into atoms in an extremely
straightforward way: Whitespace is the only separator, and it is always a
separator. This has as a consequence that the block delimiters **{** and
**}** are not allowed to appear next to a non-white character. Another
consequence is that strings cannot contain white spaces. Both these
conditions may be relaxed in the future though.

Strings are always entered
with a leading slash, and anything with a leading slash is a string, so
/this is a string. Currently, the double quote has no special
meaning, but it may acquire meaning in the future, especially as in
/"maybe the mcx future will be brighter".

Variables names are always entered with a leading dot, and anything with a
leading dot is a variable name. Variables are implicitly typed by the
object to which they are bound. There is no support for arrays (yet).
If variables are used, as in .x .y add, only the names of
the variables are popped, and the binding between the names and their
values stays intact.

There are some operators that do in-place modification of the object that
they act on, such as infl (inflation). Currently, these are all
operators acting on matrices. When presented with a variable,
they do change the object associated with the variable.
In the built-in documentation strings (accessible with **help**
and **grep**), such in-place modification is denoted with
a prime as in <m> -> <m'>.

**dup** can be used to duplicate objects. In case of variables,
this will be a copy of the variable name. If one occurrence is
used to free the associated object by some operator, all other
instances of the variable become *stale*.
**mcx** will notice this and you will see such elements marked as stale
handles in the stack listing. **copy** can be used to copy the
object associated with a variable.

**mcx** is/interprets a stack language. In order to add 1 and 2 you need to
write 1 2 add. When add is evaluated, it removes the previous two
items from the stack and replaces it with their sum, in this case the
integer 3.
**mcx**'s types are denoted as <int>, <real>, <mx>,
<str>, <block>, and so are objects that can be of that type. An
object that is of underspecified type (such as taken by overloaded
operators) is denoted <obj>.
Where a variable name must occur, <var> is written.
In **mcx** parlance a 'variable' is the same as 'handle'.
Remember that a variable named x is written .x, and
that a string "foo" is entered as /foo in **mcx**.

## OPERATORS

|  |  |  |
| --- | --- | --- |
| **help** | | |
|  |  | **help** with no arguments prints a descriptory line for each primitive. Can only be used at bottom of stack. Alias: **h**. |
|  |  |  |
| /str **help** | | |
|  |  | **help** with argument /str prints a descriptive line for the primitive named str. The string must be identical to the primitive's name, or help will not show anything. Alias: **h**. |
|  |  |  |
| /str **grep** | | |
|  |  | Prints each descriptive line in which the string str occurs (literally). So /matrix grep should produce a lot of matrix related primitives (although it may miss generic primitives such as **add** and **mul**). At bottom of stack, prints all descriptive lines. Alias: **g**. |
|  |  |  |
| **ops** | | |
|  |  | Lists all primitives and aliases concisely in a paragraph. |
|  |  |  |
| **list** | | |
|  |  | Lists the contents of the operand stack. |
|  |  |  |
| **info** | | |
|  |  | Lists the top element of the operand stack. |
|  |  |  |
| <int> **tell** | | |
|  |  | Lists the top <int> elements of the operand stack. The trio of **list**, **info**, and **tell** is perhaps overdoing it slightly, but anyway there it is. |
|  |  |  |
| **vars** | | |
|  |  | Lists all variables currently bound. |
|  |  |  |
| <obj> <var> **def** | | |
|  |  | Bind <obj> to <var>. Redefinition is allowed. |
|  |  |  |
| <var> **free** | | |
|  |  | Break the binding between <var> and the object it currently binds, and remove object. |
|  |  |  |
| **unlink** | | |
|  |  | Break the binding between <var> and the object it currently binds, and push object on the stack. |
|  |  |  |
| <int> <block1> <block2> **ifelse** | | |
|  |  | If <int> is nonzero execute <block1>, otherwise execute <block2>. The equate operators **lt**, **lq**, **eq**, **gq**, and **gt** can be used to leave an <int> on the stack. |
|  |  |  |
| <block> **do** | | |
|  |  | Execute <block> (one time). |
|  |  |  |
| <int> <block> **repeat** | | |
|  |  | Execute <block> <int> times. |
|  |  |  |
| <block1> <block2> **while** | | |
|  |  | This primitive repeatedly executes the *condition* <block1>, *which must push an integer each time it is executed*. Each time, the pushed integer is popped. If it was non-zero, <block2> is executed, <block1> is executed, and the cycle continues. If it was zero, **while** is done. |
|  |  |  |
| **pop** | | |
|  |  | Pops one element off of the stack. |
|  |  |  |
| **exch** | | |
|  |  | Exchanges the top two elements of the stack. |
|  |  |  |
| **dup** | | |
|  |  | Duplicates the top element and pushes the duplicate onto the stack. In case of a variable, the name is duplicated and not the object it points to. In case of anonymous matrices and strings (and whatever other (composite) objects may emerge), a *deep copy* is made that is completely independent of the original object. |
|  |  |  |
| <int> **mdup** | | |
|  |  | Duplicates the top <int> elements. See the **dup** entry above for the semantics of duplication. I wrote **mdup** because I thought some other operator needed it, which turned out to be false. In the meantime, I became very much attached to **mdup**, so mail me if you think you have any useful use for it. |
|  |  |  |
| **copy** | | |
|  |  | Copies the top element and pushes the copy onto the stack. In case of a variable, copies the object associated with the variable rather than the variable's name (as **dup** would do). |
|  |  |  |
| <int1> <int2> **roll** | | |
|  |  | Shift <int1> objects cyclically <int2> times to the right if <int2> is positive, to the left otherwise. |
|  |  |  |
| **clear** | | |
|  |  | Empty the stack. All objects bound to variables are unaffected. |

## INDEX

This is a list of all **mcx**'s primitives, including aliases.
Do 'mcx /<op> help' to see a short descriptive string, where <op>
is one of the primitives below (remember, strings need a leading slash).

**add** **addto** **cdim** **ch** **clear** **copy** **def** **dgt**
**dim** **div** **do** **dup** **eq** **exch** **free** **gq**
**grep** **gt** **hdm** **hdp** **help** **id** **ifelse** **imac**
**infl** **info** **jj** **lf** **list** **lm** **lq** **lt** **max**
**mdup** **min** **mod** **mul** **ops** **pop** **pow** **quit**
**rdim** **repeat** **roll** **st** **tell** **tp** **tut** **unlink**
**vars** **vb** **vm** **while** **wm** **xpn** **bye=quit** **d=def**
**f=free** **h=help** **i=info** **l=list** **p=pop** **g=grep**
**u=unlink** **x=exch**.

## AUTHOR

Stijn van Dongen.

## SEE ALSO

mclfamily for an overview of all the documentation
and the utilities in the mcl family.
