# Supplementary material for: MSOAR 2.0: Incorporating tandem duplications into ortholog assignment based on genome rearrangement
Source: BMC Bioinformatics. 2010 Jan 6;11:10. doi: 10.1186/1471-2105-11-10 (PMC2821317; doi:10.1186/1471-2105-11-10)
Supplement: Additional file 2 — contains the software MSOAR 2.0. [file 1471-2105-11-10-S2.GZ › MSOAR2.0/Programs/mcl-06-058/doc/mclfamily.html]

The MCL family


27 Feb 2006   
**The MCL family**
1.006, 06-058

|  |  |  |
| --- | --- | --- |
| 1. |  | NAME |
| 2. |  | DESCRIPTION |

## NAME

mclfamily - a description of the mcl family of cluster applications.

**mcl** is the Amsterdam implementation of the Markov Cluster Algorithm.
It is described in the mcl manual.
Several other utilities are part of the MCL distribution. This manual pages
gives an overview.

|  |  |  |
| --- | --- | --- |
| mcl |  | the cluster algorithm |
| mclfamily |  | MCL Frequently Asked Questions |
|  |  |  |
| mcxio |  | the graph/matrix input/output format |
|  |  |  |
| mcxconvert |  | convert between interchange/binary storage types |
| mcxassemble |  | create matrices from raw data |
| mcxdump |  | dump a matrix optionally with label substitions |
| mcxload |  | load label data into matrix and tab files |
| mcxarray |  | transform array data to MCL matrices |
| mcxmap |  | relabel indices in a graph/matrix |
|  |  |  |
| mcx |  | general matrix operations |
| mcxsubs |  | extracting submatrices in various ways |
|  |  |  |
| clmdist |  | compute split/join distance between clusterings |
| clminfo |  | compute performance measure for clusterings |
| clmmeet |  | compute intersection of clusterings |
| clmmate |  | find best matching clusters between clusterings |
| clmclose |  | fetch connected components from graphs or subgraphs |
| clmimac |  | interpret MCL iterand/matrix as clustering |
| clmresidue |  | extend subgraph clustering |
| clmformat |  | display clusters as html or txt files |
| clmorder |  | reorder indices to represent blocks from different clusterings |
|  |  |  |
| mclpipeline |  | parsing/assembly/clustering/display |
| mclblastline \* |  | BLAST pipeline |
| mcxdeblast \* |  | parse BLAST files |

Entries marked \* are not available if only a default
install is done.

## DESCRIPTION

mcl - the clustering program. Since the 05-314 release
it has the ability to read in and cluster label input.

mclfamily - Frequently Asked Questions.

mcxio - a description of the mcl matrix format.

mcxconvert - convert matrices from interchange mcl format to
binary mcl format or vice versa.

mcxassemble - assemble a matrix/graph from partial edge
weight scores. Useful intermediate format to be used when transforming
application specific data into an mcl input matrix.
However, **mcl** has now acquired the ability to read graphs directly
from label input, removing the need for **mcxassemble** in most cases.

mcxdump - dump matrices in a line-based format, optionally map
indices to labels. Either a node pair (matrix entry) or a node list
(matrix row) is output per line.

mcxload - load matrices and tab files
from a line-based ID1 ID2 format.
It can load bipartite structures in which the two columns
contain labels from different domains. It has many options
to further symmetrize and transform the input.

mcxarray - transform array data to MCL matrices. The data
may be of rectangular M x N type. Either an M x M
or an N x N dimensional matrix can be made, by computing
correlation scores between the vectors in one of the to domains.
The Pearson correlation coefficient and the cosine are supported,
and further tearing and pruning options can be applied.

mcxmap - relabel indices in a graph.

mcx - an interpreter for a stack language that enables
interaction with the **mcl** matrix libraries. It can be used both from the
command line and interactively, and supports a rich set of operations such
as transposition, scaling, column scaling, multiplication, Hadamard powers
and products, et cetera. The general aim is to provide handles for simple
number and matrix arithmetic,
and for graph, set, and clustering operations. The following is
a very simple example of implementing and using **mcl** in this language.

```
 2.0 .i def                    # define inflation value.
 /small lm                     # load matrix in file 'small'.
 dim id add                    # add identity matrix.
 st .x def                     # make stochastic, bind to x.
 { xpn .i infl vm } .mcl def   # define one mcl iteration.
 20 .x .mcl repeat             # iterate  20 times
 imac                          # interpret matrix as clustering.
 vm                            # view matrix (clustering).
```

One of the more interesting things that can be done is doing mcl runs
with more complicated inflation profiles than the two-constant approach used
in **mcl** itself.

mcxsubs - compute a submatrix of a given matrix, where row
and column index sets can be specified as lists of indices combined
with list of clusters in a given clustering. Useful for inspecting
local cluster structure.

clmdist - compute the split/join distance between two
partitions. The split/join distance is better suited for measuring partition
similarity than the long-known equivalence mismatch coefficient. The former
measures the number of node moves required to transform one partition into
the other, the latter measures differences between volumes of edges of
unions of complete graphs associated with partitions.

clminfo - compute a performance measure saying how well
a clustering captures the edge weights of the input graph. Useful
for comparing different clusterings on the same graph, best used in
conjunction with **clmdist** - because comparing clusterings at
different levels of granularity should somewhat change the performance
interpretation. The latter issue is discussed in the clmdist
entry.

clmmeet - compute the intersection of a set of clusterings,
i.e. the largest clustering that is a subclustering of all. Useful
for measuring the consistency of a set of different clusterings
at supposedly different levels of granularity (in conjunction with
**clmdist**).

clmmate - find best matching clusters between two different
clusterings.

clmclose - fetch connected components from graphs or subgraphs.

clmimac - interpret MCL iterands as clusterings. The clusterings
associated with early iterands may contain overlap, should you be
interested therein.

clmresidue - extend a clustering of a subgraph onto a clustering
of the larger graph.

clmformat - display clusters suitable for scrutinizing.

clmorder - reorder indices to represent blocks from
different clusterings.

mclpipeline - set up a pipeline from data parsing stage
unto clustering format/display stage.

mclblastline - BLAST specific pipeline.

mcxdeblast - BLAST parser. Can be used to directly stream
a graph into **mcl**. Can also prepare input for mcxassemble
or can be plugged into the heavy-weight mclblastline.
