# Supplementary material for: MSOAR 2.0: Incorporating tandem duplications into ortholog assignment based on genome rearrangement
Source: BMC Bioinformatics. 2010 Jan 6;11:10. doi: 10.1186/1471-2105-11-10 (PMC2821317; doi:10.1186/1471-2105-11-10)
Supplement: Additional file 2 — contains the software MSOAR 2.0. [file 1471-2105-11-10-S2.GZ › MSOAR2.0/Programs/mcl-06-058/doc/clmmeet.html]

The clmmeet manual


27 Feb 2006   
**clmmeet**
1.006, 06-058

|  |  |  |
| --- | --- | --- |
| 1. |  | NAME |
| 2. |  | SYNOPSIS |
| 3. |  | DESCRIPTION |
| 4. |  | AUTHOR |
| 5. |  | SEE ALSO |

## NAME

clmmeet - compute the intersection of a set of clusterings.

## SYNOPSIS

**clmmeet** [--adapt] [-o <output file name>] <file name>+

## DESCRIPTION

**clmmeet** computes the intersection of a set of clusterings, that is,
the largest clustering that is a subclustering of all the clusterings
in the set. It stores the intersection (or meet) in the argument
to the **-o** flag, which must be the first option given.
All remaining arguments are interpreted as names of files containing
clusterings in mcl format (see mcxio), which must all
pertain to a set of the same cardinality.

This utility can be useful in measuring (in conjunction with
clmdist) the consistency of a set of clusterings at different
levels of granularity - if the meet of all those clusterings is close to
being a subclustering of each of them, consistency is good. See
clmdist for an example.

The --adapt option allows domain mismatches to occur between clusterings.
**clmmeet** will project the clusterings onto the meet of their domains.

## AUTHOR

Stijn van Dongen.

## SEE ALSO

mclfamily for an overview of all the documentation
and the utilities in the mcl family.
