# Supplementary material for: MSOAR 2.0: Incorporating tandem duplications into ortholog assignment based on genome rearrangement
Source: BMC Bioinformatics. 2010 Jan 6;11:10. doi: 10.1186/1471-2105-11-10 (PMC2821317; doi:10.1186/1471-2105-11-10)
Supplement: Additional file 2 — contains the software MSOAR 2.0. [file 1471-2105-11-10-S2.GZ › MSOAR2.0/Programs/mcl-06-058/doc/mcxload.html]

The mcxload manual


27 Feb 2006   
**mcxload**
1.006, 06-058

|  |  |  |
| --- | --- | --- |
| 1. |  | NAME |
| 2. |  | SYNOPSIS |
| 3. |  | DESCRIPTION |
| 4. |  | OPTIONS |
| 5. |  | AUTHOR |
| 6. |  | SEE ALSO |

## NAME

mcxload - load matrices and tab files from label format

## SYNOPSIS

**mcxload** **-abc** <fname> (*label file*)
**-o** <fname> (*output file*)

**[--stream-mirror** (*symmetrify, same domain*)**]**
**[--graph** (*assume same domain*)**]**
**[-re** <mode> (*edge deduplication mode*)**]**
**[-ri** <mode> (*image symmetrification mode*)**]**
**[-cache-tab** <fname> (*save domain tab*)**]**
**[-cache-tabc** <fname> (*save column tab*)**]**
**[-cache-tabr** <fname> (*save row tab*)**]**
**[-strict-tab** <fname> (*tab universe*)**]**
**[-strict-tabc** <fname> (*tabc universe*)**]**
**[-strict-tabr** <fname> (*tabr universe*)**]**
**[-restrict-tab** <fname> (*tab world*)**]**
**[-restrict-tabc** <fname> (*tabc world*)**]**
**[-restrict-tabr** <fname> (*tabr world*)**]**
**[-extend-tab** <fname> (*tab launch*)**]**
**[-extend-tabc** <fname> (*tabc launch*)**]**
**[-extend-tabr** <fname> (*tabr launch*)**]**
**[--stream-log** (*log transform stream values*)**]**
**[--stream-neg-log** (*minus log transform stream values*)**]**
**[-stream-tf** (*transform stream values*)**]**
**[-tf** <tf-spec> (*transform (not so) final matrix*)**]**
**[-etc** <fname> (*leader 'etc' label file*)**]**
**[-etc-ai** <fname> (*leaderless 'etc' label file*)**]**
**[-t** (*transpose*)**]**
**[--binary** (*output binary format*)**]**
**[--debug** (*debug*)**]**
**[-h** (*option listing*)**]**
**[--apropos** (*option listing*)**]**
**[--version** (*version*)**]**

## DESCRIPTION

**mcxload** reads label input from a file. The format of the file
should be line-based, each line containing two white-space separated
strings (labels) and optionally a number separated from the second label
by whitespace. In the absence of a value, mcxload will use the
default value 1.0.

mcxload will transform the labels into mcl numerical identifiers and the
pairs of labels into graph edges or equivalently matrix entries.
The weight of an edge is the value associated with the associated
labels. mcxload constructs dictionaries (sometimes just one) that
map labels onto mcl identifiers as it goes along. It can optionally
write these to file.
In MCL (family) parlance, a such a dictionary written to file
is called a *tab file*.

A major mcxload modality is whether the input refers to a single
domain or to two separate domains. An example of the first is where
labels are names of people and the value is the extent to which they
like one another. This encodes a *likability* graph where all
the nodes represent people. The reasonable thing to do in this
case is to create a single dictionary with all names wherever
they occur. All **tab** options (as opposed to **tabc** and **tabr**)
pertain to this scenario and likewise for the options **--graph**
and **--stream-mirror**.

An example of the second mode is where the first label is again the name of
a person, the second label is the name of an animal species, and the value
is the extent to which that person appreciates the species. In this case,
the reasonable thing to do is to create two dictionaries, one for persons
and one for species. All **tabc** and **tabr** options pertain to
this scenario. The **tabc** options *always refer to the first label*
and the **tabr** options *always refer to the second label*.
The letters **c** and **r** refer to *column* and *row* respectively.
The latter are the names of the matrix domains corresponding
to the input domains. Refer to mcxio.

A further mcxload modality is whether it constructs dictionaries
on the fly, or whether it proceeds from a tab file already
available.
By default mcxload will construct dictionaries on the fly. You
need to save them with the appropriate **cache** option(s).
All the **strict** options read a tab file
and require any labels in the **-abc** *label input*
to be present in the corresponding tab file. mcxload will then fail in
the face of absent labels.
All the **restrict** options simply ignore labels that are
not found in the corresponding tab file.
The **extend** options extend the existing tab file with
labels that are not found.
It presumably only makes sense to do so if the corresponding
**cache** options are used as well.

The input stream is deduplicated on a per-node neighbourhood basis
using the **-re** option.

mcxload has a few options to transform or select based on
the values in the input stream and the values in the
constructed matrix. These are
**--stream-log**,
**--stream-neg-log**,
**-stream-tf** and
**-tf**.
Refer to mcxio for a description of the syntax accepted
by the latter two options - it is a syntax accepted
by a few more mcl siblings.
Finally it is possible to transpose the final result
using the **-t** option. Keep in mind that
mcxload does not accordingly change its idea of row and
column domains.

The final matrix can be symmetrified using the **-ri** option.

The **-etc** options may be useful for the odd job.
These options assume a format where all entries for a given
column (or equivalently all neighbours for a given node)
are joined onto a single line. This can be useful e.g. to read
in externally generated clusterings.

**STAGES**  
Conceptually, input matrix creation consists of the following stages

|  |  |  |
| --- | --- | --- |
| i |  | Read the input stream, apply **-stream-tf** transformation specification, and optionally push reverse elements (**--stream-mirror**). |
| ii |  | Deduplicate edges in the context of all edges/arcs originating from a given node according to the **-re** option. |
| iii |  | Apply transpose symmetrification according to the **-ri** option, if used. |
| iv |  | Apply **-tf** transformation specification. |

## OPTIONS

|  |  |  |
| --- | --- | --- |
| **-abc** <fname> (*label file*) | | |
|  |  | The file to read label data from. |
|  |  |  |
| **-o** <fname> (*output file*) | | |
|  |  | The output file where the constructed matrix is written. |
|  |  |  |
| **--stream-mirror** (*symmetrify, same domain*) | | |
|  |  | Whenever *label1* *label2* *value* is encountered in the input, mcxload inserts *label2* *label1* *value* in the input stream as well. This option implies that both labels belong to the same domain. |
|  |  |  |
| **--graph** (*assume same domain*) | | |
|  |  | This tells mcxload that both labels belong to the same domain. |
|  |  |  |
| **-re** <max\|add\|mul\|first\|last> (*deduplication mode*) | | |
|  |  | This specifies how mcxload should collapse repeated entries, that is edges for which a value is specified multiple times. This is done relative to a single node at a time, taking into account all neighbours assembled from the input stream. Note that **--stream-mirror** will result in duplicated entries if the input contains edge specifications in both ways. Also note that **first** and **last** might not result in symmetric input if only **--stream-mirror** is used. |
|  |  |  |
| **-cache-tab** <fname> (*save domain tab*) | | |
|  |  | Write the domain to file. It applies to both label types. |
|  |  |  |
| **-cache-tabc** <fname> (*save column tab*) | | |
|  |  | Write the column domain to file. It applies to the first label found on each input line. |
|  |  |  |
| **-cache-tabr** <fname> (*save row tab*) | | |
|  |  | Write the column domain to file. It applies to the second label found on each input line. |
|  |  |  |
| **-strict-tab** <fname> (*tab universe*) | | |
|  |  | Read a dictionary from file and require each label to be present in the dictionary. mcxload will exit on absentees. |
|  |  |  |
| **-strict-tabc** <fname> (*tabc universe*) | | |
|  |  | Read a dictionary from file and require the first label on each line to be present in the dictionary. mcxload will exit on absentees. |
|  |  |  |
| **-strict-tabr** <fname> (*tabr universe*) | | |
|  |  | Read a dictionary from file and require the second label on each line to be present in the dictionary. mcxload will exit on absentees. |
|  |  |  |
| **-restrict-tab** <fname> (*tab world*) | | |
|  |  | Read a dictionary from file and only accept input lines (edges) for which both labels are present in the dictionary. mcxload will ignore absentees. |
|  |  |  |
| **-restrict-tabc** <fname> (*tabc world*) | | |
|  |  | Read a dictionary from file and ignore input lines for which the first label is absent from the dictionary. |
|  |  |  |
| **-restrict-tabr** <fname> (*tabr world*) | | |
|  |  | Read a dictionary from file and ignore input lines for which the second label is absent from the dictionary. |
|  |  |  |
| **-extend-tab** <fname> (*tab launch*) | | |
|  |  | Read a dictionary from file and extend it with any label from the input not yet present in the dictionary. |
|  |  |  |
| **-extend-tabc** <fname> (*tabc launch*) | | |
|  |  | Read a dictionary from file and extend it with all first labels from the input not yet present in the dictionary. |
|  |  |  |
| **-extend-tabr** <fname> (*tabr launch*) | | |
|  |  | Read a dictionary from file and extend it with all second labels from the input not yet present in the dictionary. |
|  |  |  |
| **--stream-log** (*log transform stream values*) | | |
|  |  | Replace each entry by its natural logarithm. |
|  |  |  |
| **--stream-neg-log** (*minus log transform stream values*) | | |
|  |  | Replace each entry by the negative of its natural logarithm. This is most likely useful to convert scores that denote probabilities or p-values such as BLAST scores. |
|  |  |  |
| **-stream-tf** (*transform stream values*) | | |
|  |  | Transform the stream values as they are read in according to the syntax described in mcxio. |
|  |  |  |
| **-tf** <tf-spec> (*transform (not so) final matrix*) | | |
|  |  | Transform the matrix values after deduplication and symmetrification according to the syntax described in mcxio. |
|  |  |  |
| **-ri** (*<max\|add\|mul>*) | | |
|  |  | After the initial matrix has been assembled, it can be symmetrified by either of these options. They indicate the operation used to combine the entries of the transposed matrix and the original matrix. **mul** is special in that it treats missing entries (which are normally considered zero in mcl matrix operations) as one. |
|  |  |  |
| **-t** (*transpose*) | | |
|  |  | Write the transposed matrix to file. This is obviously not useful when a symmetric matrix has been generated. |
|  |  |  |
| **-etc** <fname> (*leader 'etc' label file*) | | |
| **-etc-ai** <fname> (*leaderless 'etc' label file*) | | |
|  |  | The input is read in lines; each line is split on whitespace into labels. For **-etc** the first label is interpreted as the source node. All other labels are interpreted as destination nodes. Currently no values are recognized, but this functionality may be introduced in the future. For **-etc-ai** (*auto-increment*) all labels are interpreted as destination nodes and mcxload automatically creates a source node for each line it reads. This option can be useful to read in files encoding a clustering, where each line represents a cluster of white-space separated labels. |
|  |  |  |
| **--binary** (*output binary format*) | | |
|  |  | Write the output matrix in native binary format. This is generally smaller and faster to read, albeit not humanly unreadable. |
|  |  |  |
| **--debug** (*debug*) | | |
|  |  | Among other things, this turns on warnings when **restrict** tab files are used and labels are found to be missing. |
|  |  |  |
| **-h** (*option listing*) | | |
|  |  | List short description of all options. |
|  |  |  |
| **--apropos** (*option listing*) | | |
|  |  | List short description of all options. |
|  |  |  |
| **--version** (*version*) | | |
|  |  | Output version information. |

## AUTHOR

Stijn van Dongen.

## SEE ALSO

mcxdump,
mcl,
mclfaq,
and mclfamily for an overview of all the documentation
and the utilities in the mcl family.
