# Supplementary material for: MSOAR 2.0: Incorporating tandem duplications into ortholog assignment based on genome rearrangement
Source: BMC Bioinformatics. 2010 Jan 6;11:10. doi: 10.1186/1471-2105-11-10 (PMC2821317; doi:10.1186/1471-2105-11-10)
Supplement: Additional file 2 — contains the software MSOAR 2.0. [file 1471-2105-11-10-S2.GZ › MSOAR2.0/Programs/mcl-06-058/doc/mcxconvert.html]

The mcxconvert manual


27 Feb 2006   
**mcxconvert**
1.006, 06-058

|  |  |  |
| --- | --- | --- |
| 1. |  | NAME |
| 2. |  | SYNOPSIS |
| 3. |  | DESCRIPTION |
| 4. |  | AUTHOR |
| 5. |  | SEE ALSO |

## NAME

mcxconvert - convert between mcx storage types

## SYNOPSIS

**mcxconvert** <input file name> <output file name>

**mcxconvert** <input file to test>

In the two-argument invocation, **mcxconvert** converts from the format
found in the first file to the other format.
In the one-argument invocation, mcxconvert will simply output
one of the strings interchange
or binary depending on the format found.

## DESCRIPTION

The **mcl** libraries make extensive use of matrices. Matrices
are used to encode graphs, matrices and clusterings.
They can be stored either in interchange or in binary format. The latter is
somewhat more efficient in storage and much faster in both reading and
writing, but the default is interchange format.

The **mcl** input routines recognize the type of storage they are dealing
with. If you want to convert a matrix to the other storage type,
simply specify the file name of the matrix you want to convert.
**mcxconvert** will recognize its type, and write the other type
to the file specified as the second argument.

In the future **mcxconvert** will probably be generalized towards
other objects such as vectors.

## AUTHOR

Stijn van Dongen.

## SEE ALSO

mcxio,
and mclfamily for an overview of all the documentation
and the utilities in the mcl family.
