# Supplementary material for: MSOAR 2.0: Incorporating tandem duplications into ortholog assignment based on genome rearrangement
Source: BMC Bioinformatics. 2010 Jan 6;11:10. doi: 10.1186/1471-2105-11-10 (PMC2821317; doi:10.1186/1471-2105-11-10)
Supplement: Additional file 2 — contains the software MSOAR 2.0. [file 1471-2105-11-10-S2.GZ › MSOAR2.0/Programs/mcl-06-058/doc/mcxarray.html]

The mcxarray manual


27 Feb 2006   
**mcxarray**
1.006, 06-058

|  |  |  |
| --- | --- | --- |
| 1. |  | NAME |
| 2. |  | SYNOPSIS |
| 3. |  | DESCRIPTION |
| 4. |  | OPTIONS |
| 5. |  | AUTHOR |
| 6. |  | SEE ALSO |

## NAME

mcxarray - Transform array data to MCL matrices

## SYNOPSIS

**mcxarray** [options] <array data matrix>

**mcxarray**
**[-o** fname (*output file fname*)**]**
**[-co** num (*cutoff for output values*)**]**
**[-gq** num (*ignore input smaller than*)**]**
**[-lq** num (*ignore input larger than*)**]**
**[-t** (*work with the transpose*)**]**
**[-ctr** num (*add center (for graph-type input)*)**]**
**[--ctr** (*add center with default value (for graph-type input)*)**]**
**[--01** (*remap output to [0,1] interval*)**]**
**[--cosine** (*use cosine*)**]**
**[--pearson** (*use Pearson correlation (default)*)**]**
**[-tear** num (*inflate input columns*)**]**
**[-teartp** num (*inflate tranposed columns*)**]**
**[-pi** num (*inflate the result*)**]**
<fname> (*array data matrix*)

## DESCRIPTION

**mcxarray** reads a data file satisfying the mcl input format
(refer to mcxio). By default it will work with the
columns as the data vectors (note that mcl matrices are
presented as a listing of columns). This can be changed using
the **-t** option.

## OPTIONS

|  |  |  |
| --- | --- | --- |
| **-o** fname (*output file fname*) | | |
|  |  | Indeed. |
|  |  |  |
| **-co** num (*cutoff for output values*) | | |
|  |  | Output values smaller than *num* are removed (set to zero). |
|  |  |  |
| **-gq** num (*ignore input smaller than*) | | |
|  |  | Input values smaller than *num* are ignored. |
|  |  |  |
| **-lq** num (*ignore input larger than*) | | |
|  |  | Input values larger than *num* are ignored. |
|  |  |  |
| **-t** (*work with the transpose*) | | |
|  |  | Work with the transpose of the input data matrix. |
|  |  |  |
| **-ctr** num (*add center (for graph-type input)*) | | |
|  |  | Add diagonal values to the output according to a (so called) *center* formula. |
|  |  |  |
| **--ctr** (*add center with default value (for graph-type input)*) | | |
|  |  | Same as **-ctr** **1.0**. |
|  |  |  |
| **--01** (*remap output to [0,1] interval*) | | |
|  |  | Rescale output values so that they are within the [0,1] interval. |
|  |  |  |
| **--cosine** (*use cosine*) | | |
|  |  | Use the cosine as correlation measure. |
|  |  |  |
| **--pearson** (*use Pearson correlation (default)*) | | |
|  |  | Compute the Pearson correleation coefficient as the similarity between the data columns. |
|  |  |  |
| **-tear** num (*inflate input columns*) | | |
|  |  | Apply (column-wise) inflation with parameter *num* to the input values before further processing. |
|  |  |  |
| **-teartp** num (*inflate tranposed columns*) | | |
|  |  | Apply (column-wise) inflation with parameter *num* to the input values in the transposed matrix before further processing. |
|  |  |  |
| **-pi** num (*inflate the result*) | | |
|  |  | Apply inflation with parameter *num* to the output values. |

## AUTHOR

Stijn van Dongen.

## SEE ALSO

mcl,
mclfaq,
and mclfamily for an overview of all the documentation
and the utilities in the mcl family.
