# Supplementary material for: MSOAR 2.0: Incorporating tandem duplications into ortholog assignment based on genome rearrangement
Source: BMC Bioinformatics. 2010 Jan 6;11:10. doi: 10.1186/1471-2105-11-10 (PMC2821317; doi:10.1186/1471-2105-11-10)
Supplement: Additional file 2 — contains the software MSOAR 2.0. [file 1471-2105-11-10-S2.GZ › MSOAR2.0/Programs/mcl-06-058/doc/mclfaq.html]

The MCL FAQ


27 Feb 2006   
**MCL FAQ**
1.006, 06-058

|  |  |  |
| --- | --- | --- |
| 1. |  | NAME |
| 2. |  | RESOURCES |
| 3. |  | TOC |
| 4. |  | FAQ |
| 5. |  | BUGS |
| 6. |  | AUTHOR |
| 7. |  | SEE ALSO |
| 8. |  | REFERENCES |
| 9. |  | NOTES |

## NAME

mclfaq - faqs and facts about the MCL cluster algorithm.

MCL refers to the generic MCL algorithm and the MCL process on which the
algorithm is based. **mcl** refers to the implementation. This FAQ answers
questions related to both. In some places MCL is written where MCL or mcl
can be read. This is the case for example in
section 3, What kind of graphs.
It should in general be obvious from the context.

This FAQ does not begin to attempt to explain the motivation
and mathematics behind the MCL algorithm - the internals are not
explained. A broad view is given in faq 1.2,
and see also faq 1.5 and section REFERENCES.

Some additional sections preceed the actual faq entries.
The TOC section contains a listing of all questions.
**Clicking on the number of a question**
(where it is answered) will take you to
the corresponding section of the table of contents.

## RESOURCES

The manual pages for all the utilities that come with **mcl**;
refer to mclfamily for an overview.

mcl development is discussed on mcl-devel@micans.org,
list information is at
https://lists.micans.org:446/listinfo/mcl-devel.

See the REFERENCES Section for publications detailing the
mathematics behind the MCL algorithm.

## TOC

---

|  |  |  |
| --- | --- | --- |
| **1** |  | **General questions** |
|  |  |  |
| **1.1** |  | **For whom is mcl and for whom is this FAQ?** |
| **1.2** |  | **What is the relationship between the MCL process, the MCL algorithm, and the 'mcl' implementation?** |
| **1.3** |  | **What do the letters MCL stand for?** |
| **1.4** |  | **How could you be so feebleminded to use MCL as abbreviation? Why is it labeled 'Markov cluster' anyway?** |
| **1.5** |  | **Where can I learn about the innards of the MCL algorithm/process?** |
| **1.6** |  | **For which platforms is mcl available?** |
| **1.7** |  | **How does mcl's versioning scheme work?** |

---

|  |  |  |
| --- | --- | --- |
| **2** |  | **Input format** |
|  |  |  |
| **2.1** |  | **How can I get my data into the MCL matrix format?** |

---

|  |  |  |
| --- | --- | --- |
| **3** |  | **What kind of graphs** |
|  |  |  |
| **3.1** |  | **What is legal input for MCL?** |
| **3.2** |  | **What is sensible input for MCL?** |
| **3.3** |  | **Does MCL work for weighted graphs?** |
| **3.4** |  | **Does MCL work for directed graphs?** |
| **3.5** |  | **Can MCL work for lattices / directed acyclic graphs / DAGs?** |
| **3.6** |  | **Does MCL work for tree graphs?** |
| **3.7** |  | **For what kind of graphs does MCL work well and for which does it not?** |
| **3.8** |  | **What makes a good input graph? How do I construct the similarities? How to make them satisfy this Markov condition?** |
| **3.9** |  | **My input graph is directed. Is that bad?** |
| **3.10** |  | **Why does mcl like undirected graphs and why does it dislike uni-directed graphs so much?** |
| **3.11** |  | **How do I check that my graph/matrix is symmetric/undirected?** |

---

|  |  |  |
| --- | --- | --- |
| **4** |  | **Speed and complexity** |
|  |  |  |
| **4.1** |  | **How fast is mcl/MCL?** |
| **4.2** |  | **What statistics are available?** |
| **4.3** |  | **Does this implementation need to sort vectors?** |
| **4.4** |  | **mcl does not compute the ideal MCL process!** |
| **4.5** |  | **I've read elsewhere that MCL is slow [compared with XYZ]** |

---

|  |  |  |
| --- | --- | --- |
| **5** |  | **Resource tuning / accuracy** |
|  |  |  |
| **5.1** |  | **What do you mean by resource tuning?** |
| **5.2** |  | **How do I compute the maximum amount of RAM needed by mcl?** |
| **5.3** |  | **How much does the mcl clustering differ from the clustering resulting from a perfectly computed MCL process?** |
| **5.4** |  | **How do I know that I am using enough resources?** |
| **5.5** |  | **Where is the mathematical analysis of this mcl pruning strategy?** |
| **5.6** |  | **What qualitative statements can be made about the effect of pruning?** |
| **5.7** |  | **At different high resource levels my clusterings are not identical. How can I trust the output clustering?** |

---

|  |  |  |
| --- | --- | --- |
| **6** |  | **Tuning cluster granularity** |
|  |  |  |
| **6.1** |  | **How do I tune cluster granularity?** |
| **6.2** |  | **The effect of inflation on cluster granularity.** |
| **6.3** |  | **The effect of similarity distribution homogeneity on cluster granularity.** |
| **6.4** |  | **The effect of initial centering on cluster granularity.** |

---

|  |  |  |
| --- | --- | --- |
| **7** |  | **Implementing the MCL algorithm** |
|  |  |  |
| **7.1** |  | **How easy is it to implement the MCL algorithm?** |

---

|  |  |  |
| --- | --- | --- |
| **8** |  | **Cluster overlap / MCL iterand cluster interpretation** |
|  |  |  |
| **8.1** |  | **Introduction** |
| **8.2** |  | **Can the clusterings returned by mcl contain overlap?** |
| **8.3** |  | **How do I obtain the clusterings associated with MCL iterands?** |

---

|  |  |  |
| --- | --- | --- |
| **9** |  | **Miscellaneous** |
|  |  |  |
| **9.1** |  | **How do I find the default settings of mcl?** |
| **9.2** |  | **What's next?** |

  
  


## FAQ

### 1 General questions

|  |  |  |
| --- | --- | --- |
| 1.1 |  | **For whom is mcl and for whom is this FAQ?** For everybody with an appetite for graph clustering. Regarding the FAQ, I have kept the amount of mathematics as low as possible, as far as matrix analysis is concerned. Inevitably, some terminology pops up and some references are made to the innards of the MCL algorithm, especially in the section on resources and accuracy. Graph terminology is used somewhat more carelessly though. The future might bring definition entries, right now you have to do without. Mathematically inclined people may be interested in the pointers found in the REFERENCES section. Given this mention of mathematics, let me point out this one time only that using **mcl** is extremely straightforward anyway. You need only mcl and an input graph (refer to the mcl manual page), and many people trained in something else than mathematics are using mcl happily. |
|  |  |  |
| 1.2 |  | **What is the relationship between the MCL process, the MCL algorithm, and the 'mcl' implementation?** **mcl** is what you use for clustering. It implements the MCL algorithm, which is a cluster algorithm for graphs. The MCL algorithm is basically a shell in which the MCL process is computed and interpreted. I will describe them in the natural, reverse, order. The MCL process generates a row of stochastic matrices given some initial stochastic matrix. The elements with even index are obtained by *expanding* the previous element, and the elements with odd index are obtained by *inflating* the previous element given some inflation constant. Expansion is nothing but normal matrix squaring, and inflation is a particular way of rescaling the entries of a stochastic matrix such that it remains stochastic. The row of MCL elements (from the MCL process) is in principle without end, but what happens is that the elements converge to some specific kind of matrix, called the *limit* of the process. The heuristic underlying MCL predicts that the interaction of expansion with inflation will lead to a limit exhibiting cluster structure in the graph associated with the initial matrix. This is indeed the case, and several mathematical results tie MCL iterands and limits and the MCL interpretation together (REFERENCES). The MCL algorithm is simply a shell around the MCL process in which an input graph is transformed into an initial matrix suitable for starting the process, in which inflation parameters are set, and in which the MCL process is stopped once the limit is reached, and in which the result is interpreted as a clustering. The **mcl** implementation supplies the functionality of the MCL algorithm, with some extra facilities for manipulation of the input graph, interpreting the result, manipulating resources while computing the process, and monitoring the state of these manipulations. |
|  |  |  |
| 1.3 |  | **What do the letters MCL stand for?** For *Markov Cluster*. The MCL algorithm is a **cluster** algorithm that is basically a shell in which an algebraic process is computed. This process iteratively generates stochastic matrices, also known as **Markov** matrices, named after the famous Russian mathematician Andrei Markov. |
|  |  |  |
| 1.4 |  | **How could you be so feebleminded to use MCL as abbreviation? Why is it labeled 'Markov cluster' anyway?** Sigh. It is a widely known fact that a TLA or Three-Letter-Acronym is *the canonical self-describing abbreviation for the name of a species with which computing terminology is infested* (quoted from the Free Online Dictionary of Computing). Back when I was thinking of a nice tag for this cute algorithm, I was totally unaware of this. I naturally dismissed *MC* (and would still do that today). Then *MCL* occurred to me, and without giving it much thought I started using it. A Google search (or was I still using Alta-Vista back then?) might have kept me from going astray. Indeed, *MCL* is used as a tag for *Macintosh Common Lisp*, *Mission Critical Linux*, *Monte Carlo Localization*, *MUD Client for Linux*, *Movement for Canadian Literacy*, and a gazillion other things - refer to the file mclmcl.txt. Confusing. It seems that the three characters MCL possess otherworldly magical powers making them an ever so strange and strong attractor in the space of TLAs. It probably helps that Em-See-Ell (Em-Say-Ell in Dutch) has some rhythm to it as well. Anyway MCL stuck, and it's here to stay. On a more general level, the label *Markov Cluster* is not an entirely fortunate choice either. Although phrased in the language of stochastic matrices, MCL theory bears very little relation to Markov theory, and is much closer to matrix analysis (including Hilbert's distance) and the theory of dynamical systems. No results have been derived in the latter framework, but many conjectures are naturally posed in the language of dynamical systems. |
|  |  |  |
| 1.5 |  | **Where can I learn about the innards of the MCL algorithm/process?** Currently, the most basic explanation of the MCL algorithm is found in the technical report [2]. It contains sections on several other (related) subjects though, and it assumes some working knowledge on graphs, matrix arithmetic, and stochastic matrices. |
|  |  |  |
| 1.6 |  | **For which platforms is mcl available?** It should compile and run on virtually any flavour of UNIX (including Linux and the BSD variants of course). Following the instructions in the INSTALL file shipped with mcl should be straightforward and sufficient. Courtesy to Joost van Baal who completely autofooled **mcl**. Building MCL on Wintel (Windows on Intel chip) should be straightforward if you use the full suite of cygwin tools. Install cygwin if you do not have it yet. In the cygwin shell, unpack mcl and simply issue the commands *./configure, make, make install*, i.e. follow the instructions in INSTALL. This MCL implementation has not yet been reported to run on MAC. For the latest Mac OS X one would expect that it is certainly possible to make this happen.  If you have further questions or news about this issue, contact mcl-devel <at> lists <dot> micans <dot> org. |
|  |  |  |
| 1.7 |  | **How does mcl's versioning scheme work?** The current setup, which I hope to continue, is this. All releases are identified by a date stamp. For example 02-095 denotes day 95 in the year 2002. This date stamp agrees (as of April 2000) with the (differently presented) date stamp used in all manual pages shipped with that release. For example, the date stamp of the FAQ you are reading is **27 Feb 2006**, which corresponds with the MCL stamp **06-058**. The Changelog file contains a list of what's changed/added with each release. Currently, the date stamp is the primary way of identifying an **mcl** release. When asked for its version by using **--version**, mcl outputs both the date stamp and a version tag (see below).  In early 2002 it occurred to me that **mcl** should, in addition to time stamps, also have something like version numbers, wanting to use those to indicate noteworthy changes. The April 2002 release got version tag 1.001, in order to celebrate the then-recent addition of this FAQ, mcl's new logging facility **--log**, and **clmimac** to the MCL distribution. The January 2003 release had its version number bumped to 1.002, marking MCL's ability to directly deal with a much more general type of graph encoding. 1.003 brought mclpipeline, mcxdeblast, mclblastline, mcxassemble, clmformat and automatic output naming. 1.004 revived binary format, improved mcxsubs, and had a host of other additions. 1.005 removed an IO bottleneck, considerably speeding up matrix reads. 1.006 featured mcl's ability to directly read and produce label output. Currently, the version tag is not used in the mcl distribution name - only the date stamp is used for that. |

### 2 Input format

|  |  |  |
| --- | --- | --- |
| 2.1 |  | **How can I get my data into the MCL matrix format?** One of the easiest ways is if you have a list of label pairs with similarities attached to the pairs. Put this information into a file with each pair of labels and the associated similarity on a single line separated by whitespace. Example:   ``` apples    oranges    2.14 oranges   pears      0.78 oranges   apples     1.57 pears     apples     1.01 ```   Suppose the file is called mydata. You can simply cluster it like this:   ```    mcl mydata --abc -I 2.0 -o mydata.cls-I20    mcl mydata --abc -I 2.4 -o mydata.cls-I24    mcl mydata --abc -I 2.8 -o mydata.cls-I28 ```   In this example a few runs were shown with different inflation parameters. This is nonsensical with this particular 4-node input but a good idea in general. **mcl** provides many options to cache the intermediate matrix input and the intermediate tab file. Refer to the mcl manual page, specifically look for the **-cache-tab** and **-cache-graph** options.  Alternatively, use mcxload to save label input as an mcl matrix and one or more tab files, then apply mcl to the matrix file using the **-use-tab** option.  **NOTE**  Simply doing   ```    mcl mydata --abc -I 2.0    mcl mydata --abc -I 2.4    mcl mydata --abc -I 2.8 ```   will result in the output files out.mydata.I20s6, out.mydata.I24s6, and out.mydata.I28s6. The s6 bit indicates the resource scheme used by mcl. The automatic naming facility is very convenient - try it. If you need to script mcl use the **-az** option to capture the name that mcl *would* generate given the rest of the command-line. |

### 3 What kind of graphs

|  |  |  |
| --- | --- | --- |
| 3.1 |  | **What is legal input for MCL?** Any graph (encoded as a matrix of similarities) that is nonnegative, i.e. all similarities are greater than or equal to zero. |
|  |  |  |
| 3.2 |  | **What is sensible input for MCL?** It is ok for graphs to be weighted, and they should preferably be symmetric. They should certainly not contain parts that are (almost) cyclic, although nothing stops you from experimenting with such input. |
|  |  |  |
| 3.3 |  | **Does MCL work for weighted graphs?** Yes, unequivocally. They should preferably be symmetric/undirected though. See entries 3.7 and 3.8. |
|  |  |  |
| 3.4 |  | **Does MCL work for directed graphs?** Maybe, with a big caveat. See entries 3.8 and 3.9. |
|  |  |  |
| 3.5 |  | **Can MCL work for lattices / directed acyclic graphs / DAGs?** Such graphs [term] can surely exhibit clear cluster structure. If they do, there is only one way for mcl to find out. You have to change all arcs to edges, i.e. if there is an arc from i to j with similarity s(i,j) - by the DAG property this implies s(j,i) = 0 - then make s(j,i) equal to s(i,j). This may feel like throwing away valuable information, but in truth the information that is thrown away (direction) is *not* informative with respect to the presence of cluster structure. This may well deserve a longer discussion than would be justified here. |
|  |  |  |
| 3.6 |  | **Does MCL work for tree graphs?** Nah, I don't think so. More info at entry 3.7. |
|  |  |  |
| 3.7 |  | **For what kind of graphs does MCL work well and for which does it not?** Graphs in which the diameter [term] of (subgraphs induced by) natural clusters is not too large. Additionally, graphs should preferably be (almost) undirected (see entry below) and not so sparse that the cardinality of the edge set is close to the number of nodes. A class of such very sparse graphs is that of tree graphs. You might look into *graph visualization* software and research if you are interested in decomposing trees into 'tight' subtrees. The diameter criterion could be violated by neighbourhood graphs derived from vector data. In the specific case of 2 and 3 dimensional data, you might be interested in *image segmentation* and *boundary detection*, and for the general case there is a host of other algorithms out there. [add] In case of weighted graphs, the notion of *diameter* is sometimes not applicable. Generalizing this notion requires inspecting the *mixing properties* of a subgraph induced by a natural cluster in terms of its spectrum. However, the diameter statement is something grounded on heuristic considerations (confirmed by practical evidence [4]) to begin with, so you should probably forget about mixing properties. |
|  |  |  |
| 3.8 |  | **What makes a good input graph? How do I construct the similarities? How to make them satisfy this Markov condition?** To begin with the last one: you *need not and must not* make the input graph such that it is stochastic aka Markovian [term]. What you need to do is make a graph that is preferably symmetric/undirected, i.e. where s(i,j) = s(j,i) for all nodes i and j. It need not be perfectly undirected, see the following faq for a discussion of that. **mcl** will work with the graph of random walks that is associated with your input graph, and that is the natural state of affairs. The input graph should preferably be honest in the sense that if s(x,y)=N and s(x,z)=200N (i.e. the similarities differ by a factor 200), then this should really reflect that the similarity of y to x is neglectible compared with the similarity of z to x. For the rest, anything goes. Try to get a feeling by experimenting. Sometimes it is a good idea to filter out high-frequency and/or low-frequency data, i.e. nodes with either very many neighbours or extremely few neighbours. |
|  |  |  |
| 3.9 |  | **My input graph is directed. Is that bad?** It depends. The class of directed graphs can be viewed as a spectrum going from undirected graphs to uni-directed graphs. *Uni-directed* is terminology I am inventing here, which I define as the property that for all node pairs i, j, at least one of s(i,j) or s(j,i) is zero. In other words, if there is an arc going from i to j in a uni-directed graph, then there is no arc going from j to i. I call a node pair i, j, *almost uni-directed* if s(i,j) << s(j,i) or vice versa, i.e. if the similarities differ by an order of magnitude. If a graph does not have (large) subparts that are (almost) uni-directed, have a go with mcl. Otherwise, try to make your graph less uni-directed. You are in charge, so do anything with your graph as you see fit, but preferably abstain from feeding mcl uni-directed graphs. |
|  |  |  |
| 3.10 |  | **Why does mcl like undirected graphs and why does it dislike uni-directed graphs so much?** Mathematically, the mcl iterands will be *nice* when the input graph is symmetric, where *nice* is in this case *diagonally symmetric to a semi-positive definite matrix* (ignore as needed). For one thing, such nice matrices can be interpreted as clusterings in a way that generalizes the interpretation of the mcl limit as a clustering (if you are curious to these intermediate clusterings, see faq entry 8.3). See the REFERENCES section for pointers to mathematical publications. The reason that mcl dislikes uni-directed graphs is not very mcl specific, it has more to do with the clustering problem itself. Somehow, directionality thwarts the notion of cluster structure. [add]. |
|  |  |  |
| 3.11 |  | **How do I check that my graph/matrix is symmetric/undirected?** Whether your graph is created by third-party software or by custom sofware written by someone you know (e.g. yourself), it is advisable to test whether the software generates symmetric matrices. This can be done as follows using the mcx utility, assuming that you want to test the matrix stored in file matrix.mci. The mcx utility should be available on your system if mcl was installed in the normal way.  ``` mcx /matrix.mci lm tp -1 mul add /check wm ```   This loads the graph/matrix stored in matrix.mci into **mcx**'s memory with the mcx *lm* primitive. - the leading slash is how strings are introduced in the stack language interpreted by **mcx**. The transpose of that matrix is then pushed on the stack with the *tp* primitive and multiplied by minus one. The two matrices are added, and the result is written to the file check. The transposed matrix is the mirrored version of the original matrix stored in matrix.mci. If a graph/matrix is undirected/symmetric, the mirrored image is necessarily the same, so if you subtract one from the other it should yield an all zero matrix. Thus, the file check *should look like this*:  ``` (mclheader mcltype matrix dimensions <num>x<num> ) (mclmatrix begin ) ```   Where <num> is the same as in the file matrix.mci. If this is not the case, find out what's prohibiting you from feeding mcl symmetric matrices. Note that any nonzero entries found in the matrix stored as check correspond to node pairs for which the arcs in the two possible directions have different weight. |

### 4 Speed and complexity

|  |  |  |
| --- | --- | --- |
| 4.1 |  | **How fast is mcl/MCL?** It's fast - here is how and why. Let N be the number of nodes in the input graph. A straigtforward implementation of MCL will have time and space complexity respecively O(N^3) (i.e. cubic in N) and O(N^2) (quadratic in N). So you don't want one of those. **mcl** implements a slightly perturbed version of the MCL process, as discussed in section Resource tuning / accuracy. Refer to that section for a more extensive discussion of all the aspects involved. This section is only concerned with the high-level view of things *and* the nitty gritty complexity details. While computing the square of a matrix (the product of that matrix with itself), mcl keeps the matrix sparse by allowing a certain maximum number of nonzero entries per stochastic column. The maximum is one of the mcl parameters, and it is typically set somewhere between 500 and 1000. Call the maximum K. mcl's time complexity is governed by the complexity of matrix squaring. There are two sub-algorithms to consider. The first is the algorithm responsible for assembling a new vector during matrix multiplication. This algorithm has worst case complexity O(K^2). The pruning algorithm (which uses heap selection) has worst case complexity O(L\*log(K)), where L is how large a newly computed matrix column can get before it is reduced to at most K entries. L is *bound by* the smallest of the two numbers N and K^2 (the square of K), but on average L will be much smaller than that, as the presence of cluster structure aids in keeping the factor L low. [Related to this is the fact that clustering algorithms are actually used to compute matrix splittings that minimize the number of cross-computations when carrying out matrix multiplication among multiple processors.] In actual cases of heavy usage, L is of order in the tens of thousands, and K is in the order of several hundreds up to a thousand. It is safe to say that in general the worst case complexity of mcl is of order O(N\*K^2); for extremely tight and dense graphs this might become O(N\*N\*log(K)). Still, these are worst case estimates, and observed running times for actual usage are much better than that. (refer to faq 4.2). In this analysis, the number of iterations required by mcl was not included. It is nearly always far below 100. Only the first few iterations are genuinely time consuming; the first few iterations (some number below 10) are usually responsible for more than 95 percent of the running time. The process of removing the smallest entries of a vector is called pruning. mcl provides extensive facilities for monitoring and controlling the effect of pruning, and it will output statistics and a summary once it is done. More information is provided in the pruning section of the mcl manual and Section 5 in this FAQ. The space complexity is of order O(N\*K). |
|  |  |  |
| 4.2 |  | **What statistics are available?** Few. Some experiments are described in [4], and [5] mentions large graphs being clustered in very reasonable time. In protein clustering, **mcl** has been applied to graphs with up to one million nodes, and on high-end hardware such graphs can be clustered within a few hours. |
|  |  |  |
| 4.3 |  | **Does this implementation need to sort vectors?** No, it does not. You might expect that one needs to sort a vector in order to obtain the K largest entries, but a simpler mechanism called *heap selection* does the job nicely. Selecting the K largest entries from a set of L by sorting would require O(L\*log(L)) operations; heap selection requires O(L\*log(K)) operations. |
|  |  |  |
| 4.4 |  | **mcl does not compute the ideal MCL process!** Indeed it does not. What are the ramifications? Several entries in section Resource tuning / accuracy discuss this issue. For a synopsis, consider two ends of a spectrum. On the one end, a graph that has very strong cluster structure, with clearly (and not necessarity fully) separated clusters. This mcl implementation will certainly retrieve those clusters if the graphs falls into the category of graphs for which mcl is applicable. On the other end, consider a graph that has only weak cluster structure superimposed on a background of a more or less random graph. There might sooner be a difference between the clustering that should ideally result and the one computed by mcl. Such a graph will have a large number of whimsical nodes that might end up either here or there, nodes that are of a peripheral nature, and for which the (cluster) destination is very sensitive to fluctutations in edge weights or algorithm parametrizations (any algorithm, not just mcl). One can say that the perturbation effect of the pruning process applied by mcl is just a small source of noise. Additionally, graphs at the noisy end of the spectrum will generally be very susceptible to changes in parametrization of the MCL algorithm and process, and the perturbation caused by computing an imperfect process will generally be small compared with the effect of changing parametrizations. Of course, there is the issue of very large and very dense graphs. The act of pruning will have a larger impact as graphs grow larger and denser. Obviously, mcl will have trouble dealing with such very large and very dense graphs - so will other methods. Finally, there is the engineering approach, which offers the possibility of pruning a whole lot of speculation. Do the experiments with **mcl**, try it out, and see what's there to like and dislike. |
|  |  |  |
| 4.5 |  | **I've read elsewhere that MCL is slow [compared with XYZ]** Presumably, they did not know mcl, and did not read the parts in [1] and [2] that discuss implementation. Perhaps they assume or insist that the only way to implement MCL is to implement the ideal process. And there is always the genuine possibility of a *really* stupifyingly fast algorithm. [One such publication is Ulrik Brandes, Marco Gaertler, and Dorothea Wagner: Experiments on Graph Clustering Algorithms. Proc. 11th Europ. Symp. Algorithms (ESA '03), Springer LNCS]. |

### 5 Resource tuning / accuracy

|  |  |  |
| --- | --- | --- |
| 5.1 |  | **What do you mean by resource tuning?** **mcl** computes a process in which stochastic matrices are alternately expanded and inflated. Expansion is nothing but standard matrix multiplication, inflation is a particular way of rescaling the matrix entries. Expansion causes problems in terms of both time and space. mcl works with matrices of dimension N, where N is the number of nodes in the input graph. If no precautions are taken, the number of entries in the mcl iterands (which are stochastic matrices) will soon approach the square of N. The time it takes to compute such a matrix will be proportional to the cube of N. If your input graph has 100.000 nodes, the memory requirements become infeasible and the time requirements become impossible. What mcl does is perturbing the process it computes a little by removing the smallest entries - it keeps its matrices *sparse*. This is a natural thing to do, because the matrices are sparse in a weighted sense (a very high proportion of the stochastic mass is contained in relatively few entries), and the process converges to matrices that are extremely sparse, with usually no more than N entries. It is thus known that the MCL iterands are sparse in a weighted sense and are usually very close to truly sparse matrices. The way mcl perturbs its matrices is by the strategy of pruning, selection, and recovery that is extensively described in the mcl manual page. The question then is: What is the effect of this perturbation on the resulting clustering, i.e. how would the clustering resulting from a *perfectly computed* mcl process compare with the clustering I have on disk? Faq entry 5.3 discusses this issue. The amount of *resources* used by mcl is bounded in terms of the maximum number of neighbours a node is allowed to have during all computations. Equivalently, this is the maximum number of nonzero entries a matrix column can possibly have. This number, finally, is the maximum of the the values corresponding with the **-S** and **-R** options. The latter two are listed when using the **-z** option (see faq 9.1). |
|  |  |  |
| 5.2 |  | **How do I compute the maximum amount of RAM needed by mcl?** It is rougly equal to  ``` 2 * s * K * N ```  bytes, where 2 is the number of matrices held in memory by **mcl**, s is the size of a single cell (c.q. matrix entry or node/arc specification), N is the number of nodes in the input graph, and where K is the maximum of the values corresponding with the **-S** and **-R** options (and this assumes that the average node degree in the input graph does not exceed K either). The value of s can be found by using the **-z** option. It is listed in one of the first lines of the resulting output. s equals the size of an int plus the size of a float, which will be 8 on most systems. The estimate above will in most cases be way too pessimistic (meaning you do not need that amount of memory). The **-how-much-ram** option is provided by mcl for computing the bound given above. This options takes as argument the number of nodes in the input graph. The theoretically more precise upper bound is slightly larger due to overhead. It is something like  ``` ( 2 * s * (K + c)) * N ```  where c is 5 or so, but one should not pay attention to such a small difference anyway. |
|  |  |  |
| 5.3 |  | **How much does the mcl clustering differ from the clustering resulting from a perfectly computed MCL process?** For graphs with up until a few thousand nodes a *perfectly computed* MCL process can be achieved by abstaining from pruning and doing full-blown matrix arithmetic. Of course, this still leaves the issue of machine precision, but let us wholeheartedly ignore that. Such experiments give evidence (albeit incidental) that pruning is indeed really what it is thought to be - a small perturbation. In many cases, the 'approximated' clustering is identical to the 'exact' clustering. In other cases, they are very close to each other in terms of the metric split/join distance as computed by clmdist. Some experiments with randomly generated test graphs, clustering, and pruning are described in [4]. On a different level of abstraction, note that perturbations of the inflation parameter will also lead to perturbations in the resulting clusterings, and surely, large changes in the inflation parameter will in general lead to large shifts in the clusterings. Node/cluster pairs that are different for the approximated and the exact clustering will very likely correspond with nodes that are in a boundary region between two or more clusters anyway, as the perturbation is not likely to move a node from one core of attraction to another. Faq entry 5.6 has more to say about this subject. |
|  |  |  |
| 5.4 |  | **How do I know that I am using enough resources?** In **mcl** parlance, this becomes *how do I know that my* **-scheme** *parameter is high enough* or more elaborately *how do I know that the values of the {-P, -S, -R, -pct} combo are high enough?* There are several aspects. First, watch the *jury marks* reported by **mcl** when it's done. The jury marks are three grades, each out of 100. They indicate how well pruning went. If the marks are in the seventies, eighties, or nineties, mcl is probably doing fine. If they are in the eighties or lower, try to see if you can get the marks higher by spending more resources (e.g. increase the parameter to the **-scheme** option). Second, you can do multiple **mcl** runs for different resource schemes, and compare the resulting clusterings using clmdist. See the clmdist manual for a case study. |
|  |  |  |
| 5.5 |  | **Where is the mathematical analysis of this mcl pruning strategy?** There is none. [add] Ok, the next entry gives an engineer's rule of thumb. |
|  |  |  |
| 5.6 |  | **What qualitative statements can be made about the effect of pruning?** The more severe pruning is, the more the computed process will tend to converge prematurely. This will generally lead to finer-grained clusterings. In cases where pruning was severe, the **mcl** clustering will likely be closer to a clustering ideally resulting from another MCL process with higher inflation value, than to the clustering ideally resulting from the same MCL process. Strong support for this is found in a general observation illustrated by the following example. Suppose u is a stochastic vector resulting from expansion:  ``` u   =  0.300 0.200 0.200 0.100 0.050 0.050 0.050 0.050 ```  Applying inflation with inflation value 2.0 to u gives  ``` v   =  0.474 0.211 0.211 0.053 0.013 0.013 0.013 0.013 ```  Now suppose we first apply pruning to u such that the 3 largest entries 0.300, 0.200 and 0.200 survive, throwing away 30 percent of the stochastic mass (which is quite a lot by all means). We rescale those three entries and obtain  ``` u'  =  0.429 0.286 0.286 0.000 0.000 0.000 0.000 0.000 ```  Applying inflation with inflation value 2.0 to u' gives  ``` v'  =  0.529 0.235 0.235 0.000 0.000 0.000 0.000 0.000 ```  If we had applied inflation with inflation value 2.5 to u, we would have obtained  ``` v'' =  0.531 0.201 0.201 0.038 0.007 0.007 0.007 0.007 ```  The vectors v' and v'' are much closer to each other than the vectors v' and v, illustrating the general idea. In practice, **mcl** should (on average) do much better than in this example. |
|  |  |  |
| 5.7 |  | **At different high resource levels my clusterings are not identical. How can I trust the output clustering?** Did you read all other entries in this section? That should have reassured you somewhat, except perhaps for Faq answer 5.5. You need not feel uncomfortable with the clusterings still being different at high resource levels, if ever so slightly. In all likelihood, there are anyway nodes which are not in any core of attraction, and that are on the boundary between two or more clusterings. They may go one way or another, and these are the nodes which will go different ways even at high resource levels. Such nodes may be stable in clusterings obtained for lower inflation values (i.e. coarser clusterings), in which the different clusters to which they are attracted are merged. By the way, you do know all about clmdist, don't you? Because the statement that clusterings are not identical should be quantified: *How much do they differ?* This issue is discussed in the clmdist manual page - clmdist gives you a robust measure for the distance (dissimilarity) between two clusterings. There are other means of gaining trust in a clustering, and there are different issues at play. There is the matter of how accurately this **mcl** computed the mcl process, and there is the matter of how well the chosen inflation parameter fits the data. The first can be judged by looking at the jury marks (faq 5.4) and applying clmdist to different clusterings. The second can be judged by measurement (e.g. use clminfo) and/or inspection (use your judgment). |

### 6 Tuning cluster granularity

|  |  |  |
| --- | --- | --- |
| 6.1 |  | **How do I tune cluster granularity?** There are several ways for influencing cluster granularity. These ways and their relative merits are successively discussed below. The clmdist(1) manual contains an example of doing multiple mcl runs for finding granularily different clusterings, using the most common approach, namely that of varying inflation. |
|  |  |  |
| 6.2 |  | **The effect of inflation on cluster granularity.** The main handle for changing inflation is the **-I** option. This is also *the* principal handle for regulating cluster granularity. Unless you are mangling huge graphs it could be the only **mcl** option you ever need besides the output redirection option **-o**. Increasing the value of **-I** will increase cluster granularity. Conceivable values are from 1.1 to 5.0 or so, but the range of suitable values will certainly depend on your input graph. For many graphs, 1.1 will be far too low, and for many other graphs, 5.0 will be far too high. You will have to find the right value or range of values by experimenting, using your judgment, and using measurement tools such as clmdist and clminfo. The default 2.0 is a good value to begin the experimental stage with. For experiments that are more subtle with respect to inflation, **mcl** provides the **-i** option in conjunction with the **-l** (small letter ell) option. Do this only if you have the intention of playing around with mcl in order to study the characteristics of the process that it computes, and *maybe*, just *maybe*, use it in a production environment if you find it useful. In the first vein, you may be interested to know that mcx is a stack language/interpreter in which the entire MCL algorithm can be written in three lines of code. It provides comprehensive access to the MCL graph and matrix libraries. However, the **mcx** interface to the MCL pruning facilities is not yet satisfactory at this time. |
|  |  |  |
| 6.3 |  | **The effect of similarity distribution homogeneity on cluster granularity.** How similarities in the input graph were derived, constructed, adapted, filtered (et cetera) will affect cluster granularity. It is important that the similarities are honest; refer to faq 3.8.  Another issue is that homogeneous similarities tend to result in more coarse-grained clusterings. You can make a set of similarities more homogeneous by applying some function to all of them, e.g. for all pairs of nodes (x y) replace S(x,y) by the square root, the logarithm, or some other convex function. Note that you need not worry about scaling, i.e. the possibly large changes in magnitude of the similarities. MCL is not affected by absolute magnitudes, it is only affected by magnitudes taken relative to each other.  As of version 03-154, mcl supports the pre-inflation **-pi** *f* option. Make a graph more homogeneous with respect to the weight function by using **-pi** with argument *f* somewhere in the interval [0,1] - 0.5 can be considered a reasonable first try. Make it less homogeneous by setting *f* somewhere in the interval [1,10]. In this case 5 is a reasonable starting point. |
|  |  |  |
| 6.4 |  | **The effect of initial centering on cluster granularity.** This refers to the **-c** parameter, which adds loops to the input graph. Its default value is 1.0, which results in loops of a somehow 'neutral' weight to be added. If you need to really fine-tune granularity, this option can be of use, otherwise you should abstain from using it. Increasing its value will increase cluster granularity.  Conceivable/normal values are in the range 1.0 to 5.0, but nothing stops you from going higher or slightly lower. Going lower than 0.5 is definitely not a good idea.  If you are into clustering at high levels of granularity, there is the issue whether to further increase **-I**, or whether to start increasing or further increase **-c**. It will really depend on the characteristics of the graph you are working with, and at this point in time I cannot even give advice in terms of a general categorization. Experiment, learn, and let me know the results if you like. |

### 7 Implementing the MCL algorithm

7.1

**How easy is it to implement the MCL algorithm?**

Very easy, if you will be doing small graphs only, say up to a few thousand
entries at most. These are the basic ingredients:

|  |  |  |
| --- | --- | --- |
| o |  | Adding loops to the input graph, conversion to a stochastic matrix. |
| o |  | Matrix multiplication and matrix inflation. |
| o |  | The interpretation function mapping MCL limits onto clusterings. |

These must be wrapped in a program that does graph input and cluster output,
alternates multiplication (i.e. expansion) and inflation in a loop, monitors
the matrix iterands thus found, quits the loop when convergence is detected,
and interprets the last iterand.

Implementing matrix muliplication is a standard exercise. Implementing
inflation is nearly trivial. The hardest part may actually be the
interpretation function, because you need to cover the corner cases of
overlap and attractor systems of cardinality greater than one.

In Mathematica or Maple, this should be doable in at most 50 lines of code.
For perl you may need 50 more lines - note that MCL does not use intricate
and expensive operations such as matrix inversion or matrix reductions. In
lower level languages such as C a basic MCL program may need a few hundred
lines, but the largest part will probably be input/output and
interpretation.

To illustrate all these points, mcl now ships with minimcl,
a small perl script that implements mcl for educational purposes.
Both its line and statement count well exceed the estimation made
above - it was not coded to be maximally succint. Its structure
is very simple though and should be easy to follow.

Implementing the basic MCL algorithm makes a
nice programming exercise. However, if you need an implementation that
scales to several hundreds of thousands of nodes and possibly beyond, then
your duties become much heavier. This is because one needs to prune MCL
iterands (c.q. matrices) such that they remain sparse. This must be done
carefully and preferably in such a way that a trade-off between speed,
memory usage, and potential losses or gains in accuracy can be controlled
via monitoring and logging of relevant characteristics.
Some other points are
i) support for threading via pthreads, openMP, or some other parallel
programming API.
ii) a robust and generic interpretation function is written in
terms of weakly connected components.

### 8 Cluster overlap / MCL iterand cluster interpretation

|  |  |  |
| --- | --- | --- |
| 8.1 |  | **Introduction** A natural mapping exists of MCL iterands to DAGs (directed acyclic graphs). This is because MCL iterands are generally *diagonally positive semi-definite* - see [3]. Such a DAG can be interpreted as a clustering, simply by taking as cores all endnodes (sinks) of the DAG, and by attaching to each core all the nodes that reach it. This procedure may result in clusterings containing overlap. In the MCL limit, the associated DAG has in general a very degenerated form, which induces overlap only on very rare occasions (see faq entry 8.2). Interpreting **mcl** iterands as clusterings may well be interesting. Few experiments have been done so far. It is clear though that early iterands generally contain the most overlap (when interpreted as clusterings). Overlap dissappears soon as the iterand index increases. For more information, consult the other entries in this section and the clmimac manual page. |
|  |  |  |
| 8.2 |  | **Can the clusterings returned by mcl contain overlap?** No. Clusterings resulting from the abstract MCL algorithm may in theory contain overlap, but the default behaviour in **mcl** is to remove it should it occur, by allocating the nodes in overlap to the first cluster in which they are seen. **mcl** will warn you if this occurs. This behaviour is switched off by supplying **--keep-overlap=yes**. Do note that overlap is mostly a theoretical possibility. It is conjectured that it requires the presence of very strong symmetries in the input graph, to the extent that there *exists an automorphism of the input graph mapping the overlapping part onto itself*. It is possible to construct (highly symmetric) input graphs leading to cluster overlap. Examples of overlap in which a few nodes are involved are easy to construct; examples with many nodes are exceptionally hard to construct. Clusterings associated with intermediate/early MCL iterands may very well contain overlap, see the introduction in this section and other entries. |
|  |  |  |
| 8.3 |  | **How do I obtain the clusterings associated with MCL iterands?** There are two options. If you are interested in clusterings containing overlap, you should go for the second. If not, use the first, but beware that the resulting clusterings may contain overlap. The first solution is to use **-dump** **cls** (probably in conjunction with either **-L** or **-dumpi** in order to limit the number of matrices written). This will cause **mcl** to write the clustering generically associated with each iterand to file. The **-dumpstem** option may be convenient as well. The second solution is to use the **-dump** **ite** option (**-dumpi** and **-dumpstem** may be of use again). This will cause **mcl** to write the intermediate iterands to file. After that, you can apply **clmimac** (interpret matrix as clustering) to those iterands. **clmimac** has a **-tight** parameter which affects the mapping of matrices to clusterings. It takes a value between 0 and 100 as argument. The default is 100 and corresponds with the strict mapping. Lowering the **-tight** value will generally result in clusterings containing more overlap. This will have the largest effect for early iterands; its effect will diminish as the iterand index increases. When set to 0, the **-tight** parameter results in the clustering associated with the DAG associated with an MCL iterand as described in [3]. This DAG is pruned (thus possibly resulting in less overlap in the clustering) by increasing the **-tight** parameter. [add] |

### 9 Miscellaneous

|  |  |  |
| --- | --- | --- |
| 9.1 |  | **How do I find the default settings of mcl?** Use **-z** to find out the actual settings - it shows the settings as resulting from the command line options (e.g. the default settings if no other options are given). |
|  |  |  |
| 9.2 |  | **What's next?** I'd like to port MCL to cluster computing, using one of the PVM, MPI, or openMP frameworks. For the 1.002 release, mcl's internals were rewritten to allow more general matrix computations. Among other things, mcl's data structures and primitive operations are now more suited to be employed in a distributed computing environment. However, much remains to be done before mcl can operate in such an environment. At some point in the future a second, xml-based, interchange input format may be introduced. If you feel that mcl should support some other standard matrix format, let us know. |

## BUGS

This FAQ tries to compromise between being concise and comprehensive. The
collection of answers should preferably cover the universe of questions at a
pleasant level of semantic granularity without too much overlap. It should
offer value to people interested in clustering but without sound
mathematical training. Therefore, if this FAQ has not failed somewhere,
it must have failed.

Send criticism and missing questions for consideration to mcl-faq at
micans.org.

## AUTHOR

Stijn van Dongen.

## SEE ALSO

mclfamily for an overview of all the documentation
and the utilities in the mcl family.

mcl's home at http://micans.org/mcl/.

## REFERENCES

[1]
Stijn van Dongen. *Graph Clustering by Flow Simulation*.
PhD thesis, University of Utrecht, May 2000.  
http://www.library.uu.nl/digiarchief/dip/diss/1895620/inhoud.htm

[2]
Stijn van Dongen. *A cluster algorithm for graphs*.
Technical Report INS-R0010, National Research Institute for Mathematics and
Computer Science in the Netherlands, Amsterdam, May 2000.  
http://www.cwi.nl/ftp/CWIreports/INS/INS-R0010.ps.Z

[3]
Stijn van Dongen. *A stochastic uncoupling process for graphs*.
Technical Report INS-R0011, National Research Institute for Mathematics and
Computer Science in the Netherlands, Amsterdam, May 2000.  
http://www.cwi.nl/ftp/CWIreports/INS/INS-R0011.ps.Z

[4]
Stijn van Dongen. *Performance criteria for graph clustering and Markov
cluster experiments*. Technical Report INS-R0012, National Research
Institute for Mathematics and Computer Science in the Netherlands,
Amsterdam, May 2000.  
http://www.cwi.nl/ftp/CWIreports/INS/INS-R0012.ps.Z

[5]
Enright A.J., Van Dongen S., Ouzounis C.A.
*An efficient algorithm for large-scale detection of protein families*,
Nucleic Acids Research 30(7):1575-1584 (2002).

## NOTES

This page was generated from **ZOEM** manual macros,
http://micans.org/zoem. Both html and roff pages can be created
from the same source without having to bother with all the usual conversion
problems, while keeping some level of sophistication in the typesetting.
This is the PostScript derived from the zoem
troff output.
