# Supplementary material for: MSOAR 2.0: Incorporating tandem duplications into ortholog assignment based on genome rearrangement
Source: BMC Bioinformatics. 2010 Jan 6;11:10. doi: 10.1186/1471-2105-11-10 (PMC2821317; doi:10.1186/1471-2105-11-10)
Supplement: Additional file 2 — contains the software MSOAR 2.0. [file 1471-2105-11-10-S2.GZ › MSOAR2.0/Programs/mcl-06-058/doc/clmimac.html]

The clmimac manual


27 Feb 2006   
**clmimac**
1.006, 06-058

|  |  |  |
| --- | --- | --- |
| 1. |  | NAME |
| 2. |  | SYNOPSIS |
| 3. |  | DESCRIPTION |
| 4. |  | OPTIONS |
| 5. |  | AUTHOR |
| 6. |  | SEE ALSO |

## NAME

clmimac - Interpret Matrices (c.q. MCL iterands output by **mcl**) As Clusterings.

## SYNOPSIS

**clmimac** -imx <fname> [options]

**clmimac**
**-imx** fname (*input file*)
**[-o** fname (*name/stem for output*)**]**
**[-dag** fname (*output DAG*)**]**
**[--enstrict** (*remove overlap*)**]**
**[-sort** str (*size|revsize|lex|none*)**]**
**[-h** (*list help*)**]**
**[--apropos** (*list synopsis of all options*)**]**


## DESCRIPTION

Use **clmimac** to interpret matrices (as clusterings) output by **mcl** using
**mcl**'s **-dump** **ite** option.

Use **clmimac** only if you have a special reason; the normal usage
of **mcl** is to do multiple runs for varying **-I** parameters
and use the clusterings output by **mcl** itself.
One reason is if you are interested in clusterings with overlap;
early MCL iterands generally induce clusterings possessing overlap.
Another reason is to investigate how the cluster structure
associated with the MCL process evolves over time.

## OPTIONS

|  |  |  |
| --- | --- | --- |
| **-imx** fname (*input file*) | | |
|  |  | The input file is presumably an MCL iterand resulting from the mcl option **-dump** **ite**. |
|  |  |  |
| **-o** fname (*file name/stem*) | | |
|  |  | Write to file named *fname*. |
|  |  |  |
| **-dag** fname (*output DAG*) | | |
|  |  | Write the DAG (directed acyclic graph) constructed from the input to file. This DAG is constructed according to the structure associated with diagonally positive semi-definite matrices as described in the PhD thesis *Graph clustering by flow simulation*. Consult mclfamily for references. |
|  |  |  |
| **-sort** str (*size\|revsize\|lex\|none*) | | |
|  |  | Sort the clusters either by increasing size, decreasing size, lexicographically by the indices they contain, or use the clustering exactly as obtained from the interpretation routine. |
|  |  |  |
| **--enstrict** (*remove overlap*) | | |
|  |  | Remove overlap should it be found, by allocating the nodes in overlap to the first cluster in which they were found. |
|  |  |  |
| **-h** (*list help*) | | |
|  |  | Print help, this could possibly be the same output as supplied with **--apropos**. |
|  |  |  |
| **--apropos** (*list synopsis of all options*) | | |
|  |  | List a one-line synopsis for all options. |

## AUTHOR

Stijn van Dongen.

## SEE ALSO

mclfamily for an overview of all the documentation
and the utilities in the mcl family.
