# Supplementary material for: MSOAR 2.0: Incorporating tandem duplications into ortholog assignment based on genome rearrangement
Source: BMC Bioinformatics. 2010 Jan 6;11:10. doi: 10.1186/1471-2105-11-10 (PMC2821317; doi:10.1186/1471-2105-11-10)
Supplement: Additional file 2 — contains the software MSOAR 2.0. [file 1471-2105-11-10-S2.GZ › MSOAR2.0/Programs/mcl-06-058/doc/mcl.html]

The mcl manual


27 Feb 2006   
**mcl**
1.006, 06-058

|  |  |  |
| --- | --- | --- |
| 1. |  | NAME |
| 2. |  | GETTING STARTED |
| 3. |  | SYNOPSIS |
| 4. |  | DESCRIPTION |
| 5. |  | OPTIONS |
| 6. |  | PRUNING OPTIONS |
| 7. |  | PIPELINES |
| 8. |  | EXAMPLES |
| 9. |  | APPLICABILITY |
| 10. |  | FILES |
| 11. |  | ENVIRONMENT |
| 12. |  | DIAGNOSTICS |
| 13. |  | BUGS |
| 14. |  | AUTHOR |
| 15. |  | HISTORY/CREDITS |
| 16. |  | SEE ALSO |
| 17. |  | REFERENCES |
| 18. |  | NOTES |

## NAME

mcl - The Markov Cluster Algorithm, aka the MCL algorithm.

**mcl** is a cluster algorithm for graphs. A single option controls the
granularity of the output clustering. This is the **-I** *inflation*
option, described further below.

## GETTING STARTED

There are two main modes of invocation. The most accessible is
*label mode*
that assumes label input. The input is then a file or stream in which each
line encodes an edge in terms of two labels and a numerical value, separated
by white space. The most basic example of usage is this:

```
   mcl <-|fname> --abc -o fname-out
```

The output is then a file where each line is a cluster of tab-separated
labels. MCL works natively with a numerical encoding of its input. This
*matrix input* is the second mode of operation. Label mode can save its
input for later use in matrix input. Here is how to do it.

```
   mcl <-|fname> --abc -o fname-out\
      -cache-tab map-name -cache-graph graph-name
```

Native mode (matrix input) is entered simply by *not* specifying
**--abc**. The input file should then have been created by an earlier
invocation of **-cache-graph** *fname* or by other means,
e.g. mcxload. It is
possible to obtain label output in native mode by specifying a label
dictionary, possibly one that was saved earlier using the
**-cache-tab** *fname* option. An example is this:

```
   mcl <-|fname> --yield-abc -o fname-out\
      -strict-tab fname-map
```

Here **-strict-tab** tells mcl that the input should not contain
labels other than found in the file **fname-map**. It is possible to
relax this by using **-restrict-tab** *fname-map* or
**-extend-tab** *fname-map*.

Label mode is very convenient for easy and fast exploration. A decided
advantage of full native mode (where both graph input and cluster output are
in matrix format) is that the data can easily be analyzed and subjected to
further processing. Second, input that is stored in native binary format
loads much faster than label data when the input data size grows large. If
neither of these is of concern then label mode may be entirely sufficient.
For more information on label mode and native mode refer to examples in this
manual and mcxio.

**Granularity**  
If you want to explore cluster structure in graphs with MCL, do use
the **-I** *inflation* option with varying parameters
to obtain clusterings at different levels of granularity.

**Clustering from blast files**  
Refer to the group of options discussed with
**--abc**. By way of a small example, consider these.

```
mcxdeblast --abc-out=- hsfsp.blast | mcl - --abc -o -
mcxdeblast --m9 --abc-out=- hsfsp.blast | mcl - --abc -o -
```

The single hyphens in this example (other than word-separators)
indicate that output is written to STDOUT or read from STDIN.
The blast files are respectively in default and column (-m8 or -m9) format.
They are parsed, the output is sent to mcl, and mcl sends a list of
tab-separated labels to STDOUT. All the abc strings serve to indicate
that the mode of communication between these programs is *label format*.
It is possible to cache the input graph in native mcl matrix format:

```
mcxdeblast --abc-out=- hsfsp.blast | \
   mcxload -abc - --stream-mirror -o hsfsp.mcx -cache-tab hsfsp.tab --binary
mcl hsfsp.mcx -use-tab hsfsp.tab -o  hsfsp.my-nice-clustering
```

The matrix is written in binary format to speed up
subsequent reads. The speed-up factor is approximately ten-fold.
For large graphs it is recommended to use binary format.
Keep in mind though that it is not portable across machines
and is not garantueed to be portable across different versions
of mcl or differently compiled versions of mcl.
**mcl** also accepts the **--binary** option, ensuring that
graphs written with **-cache-graph** are output in binary
format.
The mcxload **--mirror** option ensures that
the resulting graph is undirected. Input graphs that
are cached with mcl's **-cache-graph** option
get the same treatment.

## SYNOPSIS

The example invocation below assumes matrix input, as described in the
mcxio section. Switching to label mode requires the input file to be in
label format and the addition of the **--abc** option.

**mcl** <-|fname>
**[-I** f (*inflation*)**]**
**[-o** str (*fname*)**]**
**[-scheme** k (*resource scheme*)**]**

These options are sufficient in 95 percent of the cases or more. The first
argument must be the name of a file containing a graph/matrix in the mcl
input format, or a hyphen to read from STDIN. With respect to clustering,
only the **-I** option and
**-scheme** option are relevant and the rest is
immaterial.

A mechanism for pipelines is supported (as of the first 1.003 release).
Refer to the PIPELINES section for more information.
A prepackaged pipeline for BLAST data is present in the form of
mclblastline. As of release 1.006 a lightweight BLAST clustering
mechanism is also supported. GETTING STARTED is a small introduction,
with some examples of using BLAST results.

The full listing of **mcl** options is shown below, separated
into parts corresponding with functional aspects such
as clustering, threading, verbosity, pruning and resource management,
automatic output naming, and dumping.
The **-scheme** parameter provides a single access point to the
pruning options, and should be sufficient in most cases.
**mcl** allows comprehensive tuning and access to its internals
for those who are interested, so it has many options.

**Baseline clustering options**  
**[-I** f (*inflation*)**]**
**[-o** str (*fname*)**]**
**[-scheme** k (*resource scheme*)**]**

**Stream options**  
**[--abc** fname (*expect/write labels*)**]**
**[--expect-abc** (*expect labels*)**]**
**[--yield-abc** fname (*write labels*)**]**
**[-use-tab** fname (*use mapping to write*)**]**
**[-strict-tab** fname (*use mapping to enforce*)**]**
**[-restrict-tab** fname (*use mapping to restrict*)**]**
**[-extend-tab** fname (*use mapping and extend*)**]**
**[-cache-tab** fname (*write mapping*)**]**

**Transform options**  
**[-tf** <tf-spec> (*transform input matrix values*)**]**
**[-abc-tf** <tf-spec> (*transform input stream values*)**]**
**[--abc-log** (*take logarithm of stream values*)**]**
**[--abc-neg-log** (*take negative logarithm of stream values*)**]**

**Cache options**  
**[-cache-graph** fname (*write graph*)**]**
**[-cache-expanded** fname (*write expanded graph*)**]**
**[-ei** inflation (*use with expanded input*)**]**

**Additional clustering options**  
**[-l** n (*initial iteration number*)**]**
**[-L** n (*main iteration number*)**]**
**[-i** f (*initial inflation*)**]**

**Input manipulation options**  
**[-c** f (*centering*)**]**
**[-pi** f (*pre-inflation*)**]**
**[-pp** n (*preprune count*)**]**
**[-in-gq** f (*filter threshold*)**]**

**Clustering result options**  
**[-sort** str (*sort mode*)**]**
**[--keep-overlap=**y/n (*retain overlap*)**]**
**[--output-limit=**y/n (*write limit matrix*)**]**
**[--force-connected=**y/n (*analyze components*)**]**
**[--check-connected=**y/n (*analyze components*)**]**
**[--analyze=**y/n (*performance criteria*)**]**
**[--show-log=**y/n (*show log*)**]**
**[--append-log=**y/n (*append log*)**]**

**Verbosity options**  
**[-v** str (*verbosity type on*)**]**
**[-V** str (*verbosity type off*)**]**
**[--silent** (*very*)**]**
**[--verbose** (*very*)**]**
**[-progress** k (*gauge*)**]**
**[--show** (*print (small) matrices to screen*)**]**

**Thread options**  
**[-te** k (*#expansion threads*)**]**
**[-ti** k (*#inflation threads*)**]**
**[-t** k (*#threads*)**]**

**Output file name options**  
**[-o** str (*fname*)**]**
**[-ap** str (*use str as file name prefix*)**]**
**[-aa** str (*append str to suffix*)**]**
**[-az** (*show output file name and exit*)**]**
**[-ax** (*show output suffix and exit*)**]**

**Dump options**  
**[-dump-interval** i:j (*dump interval*)**]**
**[-dump-modulo** k (*dump modulo*)**]**
**[-dump-stem** stem (*dump file stem*)**]**
**[-dump** str (*type*)**]**
**[-dump-subi** spec (*index list for submatrix dump*)**]**
**[-dump-subd** spec (*domain list for submatrix dump*)**]**
**[-dump-dom** fname (*domain matrix file*)**]**
**[-digits** n (*printing precision*)**]**

**Info options**  
**[--jury-charter** (*explains jury*)**]**
**[--version** (*show version*)**]**
**[-how-much-ram** k (*RAM upper bound*)**]**
**[-h** (*most important options*)**]**
**[--apropos** (*one-line description for all options*)**]**
**[-z** (*show current settings*)**]**
**[-az** (*show output file name and exit*)**]**
**[-ax** (*show output suffix and exit*)**]**
**[--show-schemes** (*show resource schemes*)**]**

**Pruning options**  
The following options all pertain to the various pruning strategies that can
be employed by **mcl**. They are described in the PRUNING OPTIONS
section, accompanied by a description of the mcl pruning strategy.
If your graphs are huge
and you have an appetite for tuning, have a look at the following:

**[-p** f (*cutoff*)**]**
**[-P** n (*1/cutoff*)**]**
**[-S** n (*selection number*)**]**
**[-R** n (*recovery number*)**]**
**[-pct** f (*recover percentage*)**]**
**[-my-scheme** n (*tag custom scheme*)**]**
**[-warn-pct** n (*prune warn percentage*)**]**
**[-warn-factor** n (*prune warn factor*)**]**
**[--adapt** (*pruning*)**]**
**[-ae** f (*adaptive pruning exponent*)**]**
**[-af** f (*adaptive pruning factor*)**]**
**[-nx** x (*x window index*)**]**
**[-ny** y (*y window index*)**]**
**[-nj** j (*jury window index*)**]**
**[-nw** w (*nr of windows*)**]**
**[-nl** w (*nr of iterations*)**]**

The first argument of **mcl** must be a file name, but some options are allowed
to appear as the first argument instead. These are the options that cause
mcl to print out information of some kind, after which it will gracefully
exit. The full list of these options is

**-z**,
**-h**,
**--apropos**,
**--version**,
**--show-settings**,
**--show-schemes**,
**--jury-charter**,
**-how-much-ram** *k*.

## DESCRIPTION

**mcl** implements the **MCL algorithm**, short for the **Markov cluster
algorithm**, a cluster algorithm for graphs developed by Stijn van Dongen at
the Centre for Mathematics and Computer Science in Amsterdam, the
Netherlands. The algorithm simulates flow using two simple algebraic
operations on matrices.
The inception of this flow process and the theory behind it are
described elsewhere (see REFERENCES). Frequently asked questions
are answered in the mclfaq section.
The program described here is a fast threaded implementation written by the
algorithm's creator with contributions by several others. Anton Enright
co-implemented threading; see the HISTORY/CREDITS section for a complete
account.
See the APPLICABILITY section for a description of the type of
graph mcl likes best, and for a qualitative assessment of its speed.
**mcl** is accompanied by several other utilities for analyzing clusterings and
performing matrix and graph operations; see the SEE ALSO section.

The first argument is the input file name,
or a single hyphen to read from stdin. The rationale for
making the name of the input file a fixed parameter is that you typically do
several runs with different parameters. In command line mode it is
pleasant if you do not have to skip over an immutable parameter all the
time.

The **-I** *f* option is the main control,
affecting cluster granularity. Using **mcl** is as simple as
typing (assuming a file *proteins* contains a matrix/graph
in native matrix format)

```
mcl proteins -I 2.0
```

The above will result in a clustering written to the file
named *out.proteins.I20s2*. It is - of course -
possible to explicitly specify the name of the output file
using the **-o** *fname* option. Refer to the
**-ap** option for a description of mcl's procedure
in automatically constructing file names from it parameters.

In native mode clusterings are stored as matrices
- this is discussed in the mcxio section.
You presumably want to convert the output to something that
is easier to interpret. The native mcl matrix format is perhaps
unpleasant to parse in the quick and dirty way. You can use

```
mcl proteins -I 2.0 -use-tab proteins.tab --yield-abc
```

to get a line/tab based output format,
each line containing a cluster in the form of tab-separated labels.
Here **proteins.tab** should be a tab file previously
created by mcxdeblast or mcl.
Refer to mcxio for more information on tab files,
and the entries grouped under **--abc** for an extensive
discussion of the various ways in which mcl can combine
label format and matrix format in input and output.

In finding good **mcl** parameter settings for a particular domain,
or in finding cluster structure at different levels of granularity,
one typically runs mcl multiple times for varying values of f (refer
to the **-I** *inflation* option for further information).

**NOTE**  
mcl interprets the matrix
entries or graph edge weights as **similarities**, and it likes
**undirected input graphs** best. It can handle directed graphs, but any
node pair (i,j) for which w(i,j) is much smaller than w(j,i) or vice versa
will presumably have a slightly negative effect on the clusterings output by
mcl. Many such node pairs will have a distinctly negative effect, so try to
make your input graphs undirected. How your edge weights are computed may
affect mcl's performance. In protein clustering, one way to go is to
choose the negated logarithm of the BLAST probabilities (see
REFERENCES).

**mcl**'s default parameters should make it quite fast under almost all
circumstances. Taking default parameters, mcl has been used to generate
good protein clusters on 133k proteins, taking 10 minutes running time on a
Compaq ES40 system with four alpha EV6.7 processors. It has been applied
(with good results) to graphs with 800k nodes, and if you have the memory
(and preferably CPUs as well) nothing should stop you from going further.

For large graphs, there are several groups of parameters available for
tuning the mcl computing process, should it be necessary. The easiest thing
to do is just vary the **-scheme** option. This
triggers different settings for the group of pruning parameters
**-p/-P**, **-R**, **-S**, and
**-pct**. The default setting corresponds with
**-scheme** **6**.
There is an additional group of control parameters
**--adapt**, **-ae**,
**-af**, which may be helpful in speeding up **mcl**.
When doing multiple mcl runs for the same graphs with different
**-I** settings (for obtaining clusterings at different levels
of granularity), it can be useful to factor out the first bit
of computation that is common to all runs, by using
the **-cache-expanded** option one time
and then using **-ei** *inflation* for each run in the set.
Whether mcl considers a graph large depends mainly on the graph
connectivity; a highly connected graph on 50,000 nodes is large to
mcl (so that you might want to tune resources) whereas a sparsely
connected graph on 500,000 nodes may be business as usual.

**mcl** is a memory munger. Its precise appetite depends on the resource
settings. You can get a rough (and usually much too pessimistic) upper
bound for the amount of RAM that is needed by using the
**-how-much-ram** option. The corresponding
entry in this manual page contains the simple formula via which the upper
bound is computed.

Two other groups of interest are the thread-related
options (you can specify the number of threads to use)
**-t**, **-te**, **-ti**,
and the verbosity-related options
**--verbose**, **--silent**, **-v**,
**-V**.
The actual settings are shown with **-z**, and for graphs with
at most 12 nodes or so you can view the MCL matrix iterands on screen
by supplying **--show** (this may give some
more feeling).

MCL iterands allow a generic interpretation as clusterings as well. The
clusterings associated with early iterands may contain a fair amount of
overlap. Refer to the **-dump** option, the mclfaq
manual, and the clmimac utility (Interpret Matrices As Clusterings).
Use **clmimac** only if you have a special reason; the normal usage of **mcl** is
to do multiple runs for varying **-I** parameters and use the
clusterings output by mcl itself.

Under very rare circumstances, **mcl** might get stuck in a seemingly infinite
loop. If the number of iterations exceeds a hundred and the *chaos*
indicator remains nearly constant (presumably around value 0.37), you can
force mcl to stop by sending it the ALRM signal (usually done
by **kill -s ALRM** *pid*). It will finish the current
iteration, and interpret the last iterand a clustering. Alternatively, you
can wait and mcl might converge by itself or it will certainly stop after
10,000 iterations (the default value for the **-L** option). The
most probable explanation for such an infinite loop is that the input graph
contains the flip-flop graph of node size three as a subgraph.

The creator of this page feels that manual pages are a valuable resource,
that online html documentation is also a good thing to have, and
that info pages are way *way* ahead of their time. The
NOTES section explains how this page was created.

In the OPTIONS section options are listed in order of
importance, with related options grouped together.

## OPTIONS

|  |  |  |
| --- | --- | --- |
| **-I** f (*inflation*) | | |
|  |  | Sets the main inflation value to f. This value is the main handle for affecting cluster granularity. It is usually chosen somewhere in the range [1.2-5.0]. **-I** **5.0** will tend to result in fine-grained clusterings, and **-I** **1.2** will tend to result in very coarse grained clusterings. Your mileage will vary depending on the characteristics of your data. That is why it is a good idea to test the quality and coherency of your clusterings using clmdist and clminfo. This will most likely reveal that certain values of **-I** are simply not right for your data. The clmdist section contains a discussion of how to use the cluster validation tools shipped with **mcl** (see the SEE ALSO section).  A second option for affecting cluster granularity is the **-c** option. It may possibly increase granularity.  With low values for **-I**, like **-I** **1.2**, you should be prepared to use more resources in order to maintain quality of clusterings, i.e. increase the argument to the **-scheme** option. |
|  |  |  |
| **-o** str (*fname*) | | |
|  |  | Output the clustering to file named fname. It is possible to send the clustering to stdout by supplying **-o** **-**. If either one of **--abc**, **--yield-abc**, or **-use-tab** *tab-file* is used the output will be in label format - provided a tab file is specified or the input is in label format. Otherwise the clustering is output in the mcl matrix format; see the mcxio section for more information on this. Refer also to the group of options discussed at **--abc**.  Look at the **-ap** *prefix* option and its siblings for the automatic naming constructions employed by **mcl** if the **-o** option is not used. |
|  |  |  |
| **--abc** fname (*expect/write labels*) | | |
| **--expect-abc** (*expect labels*) | | |
| **--yield-abc** fname (*write labels*) | | |
| **-use-tab** fname (*use mapping to write*) | | |
| **-strict-tab** fname (*use mapping to enforce*) | | |
| **-restrict-tab** fname (*use mapping to restrict*) | | |
| **-extend-tab** fname (*use mapping and extend*) | | |
| **-cache-tab** fname (*write mapping*) | | |
|  |  | These items all relate to label input and/or label output. **--abc** tells mcl to expect label input and output clusters in terms of those labels. It is equivalent to the combination of **--expect-abc** and **--yield-abc**.  **-restrict-tab** and **-strict-tab** can be used both with label input and native format. When label input is used, they restrict, respectively require labels to be present in the tab file. When native input is used they restrict, respectively require indices to be present in the tab domain. **-strict-tab** fails in the face of exceptions, **-restrict-tab** will simply ignore them. **-strict-tab** and **-restrict-tab** do *not* automatically yield label output. You need **--abc** or **--yield-abc**.  **-extend-tab** is only useful when label input is used. It will create a new label/index mapping when a label is not found in the tab file. Presumably you want to use the **-cache-tab** as well then.  **-use-tab** is only useful when matrix input is used. It will use the tab file to convert the output to labels; it does not hitch on indices missing from the tab file. Take a pick from **-restrict-tab** or **-strict-tab** *and* **--abc** or **--yield-abc** if that is what you want.  **-cache-tab** can be used to preserve the tab file that was constructed from label input, either from scratch or by extension from a previous tab file in case **-extend-tab** was used.  **NOTE**  in all its dealings with tab files, mcl will only accept those for which the associated domain is *canonical*, that is, domains for which the indices range from zero to some number N without omissions. It is possible to hook up any tab file to mcl, but it requires mcxdump to act as an intermediary - mcxdump has no such limitations. This need in general not be of concern to you. If a tab file is created by mcl or mcxdeblast it will always be canonical. |
|  |  |  |
| **-tf** <tf-spec> (*transform input matrix values*) | | |
| **-abc-tf** <tf-spec> (*transform input stream values*) | | |
| **--abc-log** (*take logarithm of stream values*) | | |
| **--abc-neg-log** (*take negative logarithm of stream values*) | | |
|  |  | **-tf** transforms the values of the input matrix according to **<tf-spec>**. **-abc-tf** transforms the stream values (when **--abc** or **--expect-abc** is used) according to **<tf-spec>**. **--abc-neg-log** and **--abc-log** respectively imply that the stream input values are first replaced by their (negative) logarithm. The reason for their existence is documented in mcxio. For a description of the transform language excpected/accepted in **<tf-spec>** refer to the same. |
|  |  |  |
| **-cache-graph** fname (*write graph*) | | |
| **-cache-expanded** fname (*write expanded graph*) | | |
| **-ei** inflation (*use with expanded input*) | | |
|  |  | If you work with label input, **-cache-graph** can be used to save the matrix mcl constructs. In a following mcl invocation, you can use this graph rather than the label input. This should greatly speed up matters. Presumably the first time around you have use the **-cache-tab** *my.cache* option. You can put that my.cache file to good use by passing it in as **-use-tab** *my.cache* the second time around. The session below puts everything together.   ``` mcl xyz.data --abc -I 2.0 -o xyz.cls-I20 --binary\             -cache-graph xyz.mci -cache-tab xyz.tab mcl xyz.mci -I 2.4 -use-tab xyz.tab -o xyz.cls-I24 ```   The **--binary** option is useful for large graphs as it will drastically speed up subsequent load times. Otherwise it is not necessary, and bear in mind that binary format is not portable across machines and it is not garantueed to be portable across different version of mcl. For very large graphs it could be a bit wasteful to load a large tab structure into memory. The second line can then be replaced by   ``` mcl xyz.mci -I 2.4 -o -\|\ mcxdump -imx - --no-values --dump-rlines\             -tabr xyz.tab -o xyz.cls-I24 ```   Admittedly this is beginning to look like black magic, but truthfully it is not, my dear paduan. mcxdump simply needs to be told how it should format its output. It needs to know it should dump the matrix columns (clusters in this case) on a per-line basis, that it should not dump the index that identifies the cluster (an arbitrary rank in our case), and that there is no need to output values. **mcxdump** furthermore does not know how the tab file relates to its input matrix, whereas mcl previously had the advantage of knowing. Hence **-tabr** is telling mcxdump that the *row domain* in the clustering matrix identify the nodes.  The first step in almost any mcl run is that the matrix constructed from the input is *squared* or *expanded*. This is a rather costly operation if the input size is large. When you are doing multiple runs it can thus be useful to cache the expanded matrix at the cost of a little more hassle. Use **-cache-expanded** *fname* to write this matrix to *fname*. In subsequent runs supply *fname* as the input argument to mcl and use **-ei** *num* to indicate that the first thing to apply should be inflation with parameter *num*. Combining our previous two examples then yields   ``` mcl xyz.data --abc -I 2.0 -o xyz.cls-I20 --binary\             -cache-expanded xyz.mxp -cache-tab xyz.tab mcl xyz.mxp -ei 2.4 -I 2.4 -o -\|\ mcxdump -imx - --no-values --dump-rlines\             -tabr xyz.tab -o xyz.cls-I24 ```   Behold, this is a very time and space efficient setup. |
|  |  |  |
| **-scheme** k (*use a preset resource scheme*) | | |
|  |  | There are currently seven different resource schemes, indexed 1..7. High schemes result in more expensive computations that may possibly be more accurate. The default scheme is 4. When **mcl** is done, it will give a grade (the so called *jury synopsis*) to the appropriateness of the scheme used. *A low grade does not necessarily imply that the resulting clustering is bad* - but anyway, a low grade should be reason to try for a higher scheme. The grades are listed in the PRUNING OPTIONS section under the **-nj** option.  The PRUNING OPTIONS section contains an elaborate description of the way **mcl** manages resources, should you be interested. In case you are worried about the validation of the resulting clusterings, the mclfaq section has several entries discussing this issue. The bottom line is that you have to compare the clusterings resulting from different schemes (and otherwise identical parameters) using utilities such as clmdist, clminfo on the one hand, and your own sound judgment on the other hand.  If your input graph is extremely dense, with an average node degree (i.e. the number of neighbours per node) that is somewhere above 500, you may need to filter the input graph by removing the nodes of highest degree (and projecting them back onto the resulting clustering afterwards) or by using the **-pp** option. |
|  |  |  |
| **--show-schemes** (*show preset resource schemes*) | | |
|  |  | Shows the explicit settings to which the different preset schemes correspond. The characteristics are written in the same format (more or less) as the output triggered by **-v** **pruning**. |
|  |  |  |
| **-c** f (*centering*) | | |
|  |  | The larger the value of f the more nodes are attached to themselves rather than their neighbours, the more expansion (the spreading of flow through the graph) is opposed, and the more fine-grained clusterings tend to be. f should be chosen greater than or equal to 1.0. The default is f=1.0. This option has a much weaker effect than the **-I** option, but it can be useful depending on your data. |
|  |  |  |
| **-v** str (*verbosity type on*) | | |
|  |  | See the **--verbose** option below. |
|  |  |  |
| **-V** str (*verbosity type off*) | | |
|  |  | See the **--verbose** option below. |
|  |  |  |
| **--silent** (*very*) | | |
|  |  | See the **--verbose** option below. |
|  |  |  |
| **--verbose** (*very*) | | |
|  |  | These are the different verbosity modes: **progress**  **pruning**  **explain**  **clusters**  **all**  where *all* means all three previous modes. **--verbose** and **-v** **all** turn them all on, **--silent** and **-V** **all** turn them all off. **-v** *str* and **-V** *str* turn on/off the single mode *str* (for *str* equal to one of **progress**, **pruning**, or **explain**). Each verbosity mode is given its own entry below. |
|  |  |  |
| **-v** **progress** | | |
|  |  | This mode causes **mcl** to emit a gauge for each single matrix multiplication. It uses some default length for the gauge, which can be altered by the **-progress** *k* option. Simply using the latter will also turn on this verbosity mode. This mode can give you quickly an idea how long an mcl run might take. If you use threading (see the **-t** option and its friends), this option may slow down the program a little (relative to **-V** **progress**, not relative to a single-CPU mcl run). |
|  |  |  |
| **-v** **explain** | | |
|  |  | This mode causes the output of explanatory headers illuminating the output generated with the **pruning** verbosity mode. |
|  |  |  |
| **-v** **pruning** | | |
|  |  | This mode causes output of resource-related quantities. It has a separate entry in the PRUNING OPTIONS section. |
|  |  |  |
| **-v** **clusters** | | |
|  |  | This mode prints a terse list of characteristics of the clusterings associated with intermediate iterands. |
|  |  |  |
| **-progress** k (*gauge*) | | |
|  |  | If k>0 then for each matrix multiplication **mcl** will print an ascii gauge telling how far it is. The gauge will be (in some cases approximately) k characters long. If k<0 then mcl will emit a gauge that is extended by one character after every \|k\| vectors computed. For large graphs, this option has been known to ease the pain of impatience. If k=0 then mcl will print a message only after every matrix multiplication, and not during matrix multiplication. This can be useful when you want mcl to be as speedy as possible, for example when using parallellized mode (as monitoring progress requires thread communication). For parallellization (by threading) see the **-t** option. |
|  |  |  |
| **-aa** str (*append str to suffix*) | | |
|  |  | See the **-ap** option below. |
|  |  |  |
| **-ap** str (*use str as file name prefix*) | | |
|  |  | If the **-o** *fname* option is not used, **mcl** will create a file name (for writing output to) that should uniquely characterize the important parameters used in the current invocation of mcl. The default format is **out.fname.suf**, where **out** is simply the literal string out, **fname** is the first argument containing the name of the file (with the graph) to be clustered, and where **suf** is the suffix encoding a set of parameters (described further below). The **-ap** *str* option specifies a prefix to use rather than **out.fname** as sketched above. However, **mcl** will interpret the character '=', if present in *str*, as a placeholder for the input file name.  If the **-aa** *str* option is used, **mcl** will append **str** to the suffix **suf** created by itself. You can use this if you need to encode some extra information in the file name suffix.  The suffix is constructed as follows. The **-I** *f* and **-scheme** parameter are always encoded. The **-pi** *f*, **-l** *k*, **-i** *f*, and **-c** *f* options are only encoded if they are used. Any real argument *f* is encoded using *exactly one* trailing digit behind the decimal separator (which itself is not written). The setting **-I** **3.14** is thus encoded as I31. The **-scheme** option is encoded using the letter 's', all other options mentioned here are encoded as themselves (stripped of the hyphen). For example   ``` mcl small.mci -I 3 -c 2.5 -pi 0.8 -scheme 5 ```   results in the file name out.small.mci.I30s5c25pi08. If you want to know beforehand what file name will be produced, use the **-az** option. |
|  |  |  |
| **-az** (*show output file name and exit*) | | |
| **-ax** (*show output suffix and exit*) | | |
|  |  | If **mcl** automatically constructs a file name, it can be helpful to known beforehand what that file name will be. Use **-az** and mcl will write the file name to STDOUT and exit. This can be used if mcl is integrated into other software for which the automatic creation of unique file names is convenient. By default MCL incorporates the input file name into the output file name and appends a short suffix describing the most important option settings. Use **-ax** to find out what that suffix is. This can be useful in wrapper pipeline scripts such as clxcoarse. |
|  |  |  |
| **-te** k (*#expansion threads*) | | |
|  |  | See the **-t** *k* option below. |
|  |  |  |
| **-ti** k (*#inflation threads*) | | |
|  |  | See the **-t** *k* option below. |
|  |  |  |
| **-t** k (*#threads*) | | |
|  |  | The **-t** options are self-explanatory. Note that threading inflation is hardly useful, as inflation is orders of magnitude faster than expansion. Also note that threading is only useful if you have a multi-processor system.  When threading, it is best not to turn on pruning verbosity mode if you are letting mcl run unattended, unless you want to scrutinize its output later. This is because it makes **mcl** run somewhat slower, although the difference is not dramatic. |
|  |  |  |
| **-l** n (*initial iteration number*) (small letter ell) | | |
|  |  | The number of times **mcl** will use a different inflation value before it switches to the (main) inflation given by the **-I** (capital eye) option. The different value is called *initial inflation* and is tunable using the **-i** *f* option (default value f=2.0). The default value (to **-l**) is zero. This option supplies new ways of affecting cluster granularity, e.g. by supplying  ``` mcl proteins -i 1.4 -l 2 -I 4.0 ```   one lets expansion prevail during the first two iterations, followed by inflation catching up (in a figurative way of writing). This may be useful in certain cases, but this type of experiment is *certainly secondary* to simply varying **-I** (capital eye). |
|  |  |  |
| **-L** n (*main iteration number*) | | |
|  |  | Normally, **mcl** computes the MCL process until it has converged fully to a doubly idempotent matrix. The number of iterations required is typically somewhere in the range 10-100. The first few iterations generally take the longest time. The **-L** option can be used to specify the number of iterations mcl may do at most. When this number is reached, mcl will output the clustering associated with the iterand last computed. |
|  |  |  |
| **-i** f (*initial inflation*) | | |
|  |  | The inflation value used during the first n iterations, where n is specified by the **-l** (ell) option. By default, n=0 and f=2.0. |
|  |  |  |
| **-pi** f (*pre-inflation*) | | |
|  |  | If used, **mcl** will apply inflation one time to the input graph before entering the main process. This can be useful for making the edge weights in a graph either more homogeneous (which may result in less granular clusterings) or more heterogeneous (which may result in more granular clusterings). Homogeneity is achieved for values *f* less than one, heterogeneity for values larger than one. Values to try are normally in the range [2.0,10.0]. |
|  |  |  |
| **-di** i:j (*dump interval*) | | |
| **-di** *all* | | |
| **-dump-interval** *i:j* | | |
|  |  | Dump during iterations i..j-1. Use *all* to dump in all iterations. See the **-dump** *str* option below. |
|  |  |  |
| **-dm** k (*dump i+0..i+k..*) | | |
| **-dump-modulo** *k* | | |
|  |  | Sampling rate: select only these iterations in the dump interval. See the **-dump** *str* option below. |
|  |  |  |
| **-ds** stem (*file stem*) | | |
| **-dump-stem** *stem* | | |
|  |  | Set the the stem for file names of dumped objects (default *mcl*). See the **-dump** *str* option below. |
|  |  |  |
| **-dump-subi** spec (*index list for submatrix dump*) | | |
| **-dump-subd** spec (*domain list for submatrix dump*) | | |
| **-dump-dom** fname (*domain matrix file*) | | |
|  |  | **-dump-subi** specifies a range of indices which will be used to select the extended principal submatrix. Argument *spec* can be a comma-separated list of single integers and integer ranges. Ranges are denoted by two integers separated by a hyphen. If **-dump-dom** is used and specifies a matrix file, the indices specified in the **-dump-subd** option should index columns in that matrix. These columns are merged and added to the list of entries used in selecting the extended principal submatrix. |
|  |  |  |
| **-dump** str (*type*) | | |
|  |  | *str* is checked for substring occurrences of the following entries. Repeated use of **-dump** is also allowed. **ite**  **dag**  **cls**  **chr**  **lines**  **cat**  **lines** and **cat** change the mode of dumping. The first changes the dump format to a line based pairwise format rather than the default mcl matrix format. The second causes all dumped items to be dumped to the default stream used for the output clustering, which is appended at the end.  The **ite** option writes **mcl** iterands to file. The **cls** option writes clusterings associated with mcl iterands to file. These clusters are obtained from a particular directed acyclic graph (abbreviated as DAG) associated with each iterand. The **dag** option writes that DAG to file. The DAG can optionally be further pruned and then again be interpreted as a clustering using clmimac, and clmimac can also work with the matrices written using the **ite** option. It should be noted that clusterings associated with intermediate iterands may contain overlap, which is interesting in many applications. For more information refer to mclfaq and the REFERENCES section below.  The **result** option dumps the usual MCL clustering.  The **chr** option says, for each iterand I, to output a matrix C with characteristics of I. C has the same number of columns as I. For each column k in C, row entry 0 is the diagonal or 'loop' value of column k in I *after* expansion and pruning, and *before* inflation and rescaling. Entry 1 is the loop value *after* inflation and rescaling. Entry 2 is the center of column k (the sum of its entries squared) computed *after* expansion and *before* pruning, entry 3 is the maximum value found in that column at the same time. Entry 4 is the amount of mass kept for that column *after pruning*.  The **-ds** option sets the stem for file names of dumped objects (default *mcl*). The **-di** and **-dm** options allow a selection of iterands to be made. |
|  |  |  |
| **-digits** n (*printing precision*) | | |
|  |  | This has two completely different uses. It sets the number of decimals used for pretty-printing **mcl** iterands when using the **--show** option (see below), and it sets the number of decimals used for writing the expanded matrix when using the **-cache-expanded** option. |
|  |  |  |
| **--show** (*print matrices to screen*) | | |
|  |  | Print matrices to screen. The number of significant digits to be printed can be tuned with **-digits** *n*. An 80-column screen allows graphs (matrices) of size up to 12(x12) to be printed with three digits precision (behind the comma), and of size up to 14(x14) with two digits. This can give you an idea of how **mcl** operates, and what the effect of pruning is. Use e.g. **-S** **6** for such a small graph and view the MCL matrix iterands with **--show**. |
|  |  |  |
| **--binary** (*output format*) | | |
|  |  | Write matrix dump output in binary mcl format rather than interchange mcl format (the default). Note that mcxconvert can be used to convert each one into the other. See mcxio and mcxconvert for more information. |
|  |  |  |
| **-sort** str (*sort mode*) | | |
|  |  | *str* can be one of **lex**, **size**, **revsize**, or **none**. The default is 'revsize', in which the largest clusters come first. If the mode is 'size', smallest clusters come first, if the mode is 'lex', clusters are ordered lexicographically, and if the mode is 'none', the order is the same as produced by the procedure used by mcl to map matrices onto clusterings. |
|  |  |  |
| **--keep-overlap** y/n (*retain overlap*) | | |
|  |  | The **keep-overlap** action causes mcl to retain overlap should this improbable event occur. In theory, **mcl** may generate a clustering that contains overlap, although this almost never happens in practice, as it requires some particular type of symmetry to be present in the input graph (not just any symmetry will do). Mathematically speaking, this is a conjecture and not a theorem, but the present author wil eat his shoe if it fails to be true (for marzipan values of shoe). It is easy though to construct an input graph for which certain mcl settings result in overlap - for example a line graph on an odd number of nodes. The default is to remove overlap should it occur.  This option has more than theoretical use because **mcl** is able to generate clusterings associated with intermediate iterands. For these clusterings, overlap is more than a theoretical possibility, and will often occur. If you specify the **-L** *k* option, mcl will output the clustering associated with the last iterand computed, and it may well contain overlap.  This option has no effect on the clusterings that are output when using **-dump** *cls* - the default for those is that overlap is not touched, and this default can not yet be overridden. |
|  |  |  |
| **--force-connected**=y/n (*analyze components*) | | |
| **--check-connected**=y/n (*analyze components*) | | |
|  |  | If the input graph has strong bipartite characteristics, mcl may yield clusters that do not correspond to connected components in the input graph. Turn one of these modes on to analyze the resultant clustering. If loose clusters are found they will be split into subclusters corresponding to connected components. With **--force-connected**=*y* mcl will write the corrected clustering to the normal output file, and the old clustering to the same file with suffix orig. With **--check-connected**=*y* mcl will write the loose clustering to the normal output file, and the corrected clustering to the same file with suffix coco.  These options are not on by default, as the analysis is currently (overly) time-consuming and mcl's behaviour actually makes some sense (when taking bipartite characteristics into account). |
|  |  |  |
| **--output-limit**=y/n (*write limit matrix*) | | |
|  |  | This will write the limit matrix to a file named **base**-limit. |
|  |  |  |
| **--analyze**=y/n (*performance criteria*) | | |
|  |  | With this mode turned on, **mcl** will reread the input matrix and compute a few performance criteria and attach them to the output file. Off by default. |
|  |  |  |
| **--append-log**=y/n (*append log*) | | |
|  |  | Appends a log with the process characteristics to the output file. By default, this mode is on. |
|  |  |  |
| **--show-log**=y/n (*show log*) | | |
|  |  | Shows the log with process characteristics on STDOUT. By default, this mode is off. |
|  |  |  |
| **-in-gq** f (*filter threshold*) | | |
|  |  | mcl will remove any edges in the input graph (equivalently, entries in the input matrix) for which the weight is below *f*. |
|  |  |  |
| **-pp** n (*preprune count*) | | |
|  |  | For each column vector (node) in the input matrix (graph) **mcl** will keep the n entries (outgoing edges) of that vector (node) that have largest weight and remove the rest. |
|  |  |  |
| **--jury-charter** (*explains jury*) | | |
|  |  | Explains how the jury synopsis is computed from the jury marks. |
|  |  |  |
| **--version** (*show version*) | | |
|  |  | Show version. |
|  |  |  |
| **-how-much-ram** n (*RAM upper bound*) | | |
|  |  | **n** is interpreted as the number of nodes of an input graph. mcl will print the maximum amount of RAM it needs for its computations. The formula for this number in bytes is:  ```    2 * c * k * n     2  :  two matrices are concurrently held in memory.    c  :  mcl cell size (as shown by -z).    n  :  graph cardinality (number of nodes).    k  :  MAX(s, r).    s  :  select number (-S, -scheme options).    r  :  recover number (-R, -scheme options). ```   This estimate will usually be too pessimistic. It does assume though that the average node degree of the input graph does not exceed k. The **-how-much-ram** option takes other command-line arguments into account (such as **-S** and **-R**), and it expresses the amount of RAM in megabyte units. |
|  |  |  |
| **-h** (*show help*) | | |
|  |  | Shows a selection of the most important **mcl** options. |
|  |  |  |
| **--apropos** (*show help*) | | |
|  |  | Gives a one-line description for all options. |
|  |  |  |
| **--show-settings** (*show settings*) | | |
|  |  | A synonym for the **-z** option. |
|  |  |  |
| **-z** (*show settings*) | | |
|  |  | Show current settings for tunable parameters. **--show-settings** is a synonym. |

## PRUNING OPTIONS

|  |  |  |
| --- | --- | --- |
| **-p** f (*cutoff*) | | |
| **-P** n (*1/cutoff*) | | |
| **-S** s (*selection number*) | | |
| **-R** r (*recover number*) | | |
| **-pct** pct (*recover percentage*) | | |
| **-my-scheme** n (*tag custom scheme*) | | |
|  |  | After computing a new (column stochastic) matrix vector during expansion (which is matrix multiplication c.q. squaring), the vector is successively exposed to different pruning strategies. The intent of pruning is that many small entries are removed while retaining much of the stochastic mass of the original vector. After pruning, vectors are rescaled to be stochastic again. MCL iterands are theoretically known to be sparse in a weighted sense, and this manoever effectively perturbs the MCL process a little in order to obtain matrices that are genuinely sparse, thus keeping the computation tractable. An example of monitoring pruning can be found in the discussion of **-v** **pruning** at the end of this section. **mcl** proceeds as follows. First, entries that are smaller than *cutoff* are removed, resulting in a vector with at most *1/cutoff* entries. The cutoff can be supplied either by **-p**, or as the inverse value by **-P**. The latter is more intuitive, if your intuition is like mine (and the P stands for precision or pruning by the way). The cutoff just described is rigid; it is the same for all vectors. The **--adapt** option causes the computation of a cutoff that depends on a vector's homogeneity properties, and this option may or may not speed up mcl.  Second, if the remaining stochastic mass (i.e. the sum of all remaining entries) is less than *pct*/100 and the number of remaining entries is less than *r* (as specified by the **-R** flag), **mcl** will try to regain ground by recovering the largest discarded entries. The total number of entries is not allowed to grow larger than *r*. If recovery was not necessary, mcl tries to prune the vector further down to at most *s* entries (if applicable), as specified by the **-S** flag. If this results in a vector that satisfies the recovery condition then recovery is attempted, exactly as described above. The latter will not occur of course if *r* <= *s*.  The default setting is something like **-P** **4000** **-S** **500** **-R** **600**. Check the **-z** flag to be sure. There is a set of precomposed settings, which can be triggered with the **-scheme** *k* option. *k*=4 is the default scheme; higher values for *k* result in costlier and more accurate computations (vice versa for lower, cheaper, and less accurate). The schemes are listed using the **--show-schemes** option. It is advisable to use the **-scheme** option only in interactive mode, and to use the explicit expressions when doing batch processing. The reason is that there is *no guarantee whatsoever* that the schemes will not change between different releases. This is because the scheme options should reflect good general purpose settings, and it may become appararent that other schemes are better.  Note that 'less accurate' or 'more accurate' computations may still generate the same output clusterings. Use **clmdist** to compare output clusterings for different resource parameters. Refer to clmdist for a discussion of this issue.  The **-my-scheme** **n** option sets a tag that is used in constructing the default output naming file. If not used, **mcl** will create a relatively long string describing the settings of the **-P**, **-pct**, **-R**, and **-S** parameters, e.g. **P600Q85R1000S1200** (where **Q** tags the *pct* setting). If used, mcl will simply use the tag **s***n*. |
|  |  |  |
| **-warn-pct** k (*prune warn percentage*) | | |
| **-warn-factor** k (*prune warn factor*) | | |
|  |  | The two options **-warn-pct** and **-warn-factor** relate to warnings that may be triggered once the *initial* pruning of a vector is completed. The idea is to issue warnings if initial pruning almost completely destroys a computed vector, as this may be a sign that the pruning parameters should be changed. It depends on the mass remaining after initial pruning whether a warning will be issued. If that mass is less than *warn-pct* or if the number of remaining entries is smaller by a factor *warn-factor* than both the number of entries originally computed *and* the recovery number, in that case, **mcl** will issue a warning. **-warn-pct** takes an integer between 0 and 100 as parameter, **-warn-factor** takes a real positive number. They default to something like 30 and 50.0. If you want to see less warnings, decrease *warn-pct* and increase *warn-factor*. Set *warn-factor* to zero if you want no warnings. |
|  |  |  |
| **--dense** (*allow matrices to fill*) | | |
|  |  | This renders all pruning options useless except for one. After each expansion step, **mcl** will remove all entries that are smaller than the threshold specified by **-p** or **-P**, which acts like a precision in this case. After removal, the matrix columns are rescaled to be stochastic again. If the **-p** threshold (precision) is zero or very small, the **--dense** option results in a rather accurate and very costly computation of the MCL process. Do not use this option for graphs with more than several thousands of entries, or you will have trouble digging your processor out of swap. |
|  |  |  |
| **-ae** f (*adaptive pruning exponent*) | | |
|  |  | See the **--adapt** option below. |
|  |  |  |
| **-af** f (*adaptive pruning factor*) | | |
|  |  | See the **--adapt** option below. |
|  |  |  |
| **--adapt** (*pruning*) | | |
|  |  | The default **mcl** pruning behaviour as described under the **-P** option is called *rigid pruning*, refering to the fact that the first stage of pruning removes entries smaller than a fixed threshold. The options discussed here enable the computation of a threshold that depends on the homogeneity characteristics of a vector. This behaviour is triggered by supplying **--adapt**. The **--adapt** behaviour only affects the first pruning stage, c.q. the computation of the first threshold (see the discussion under the **-P** option). It does not interfere with either selection or recovery. It is affected however by the threshold as specified by the **-P** option. When using **--adapt**, you typically use the **-P** option as well, and you can and should use a higher value then you would without using **--adapt**.  All that said, **--adapt** triggers this behaviour: Given a stochastic vector v, its mass center of order two is computed, which is the sum of each entry squared. The mass center of v, call it c, is strongly related to its homogeneity properties (see REFERENCES). The threshold T is computed as 1/f \* pow(c, e), where e and f are the arguments to the **-af** *f* and **-ae** *e* options respectively (check **-z** for the respective defaults). For either e or f decreasing it means that T becomes larger. *Finally, T is maxed with the rigid threshold value*, which can be altered using either **-p** *f* or **-P** *n*. The latter is why you should increase the **-P** parameter n (so that the rigid threshold is decreased) once you switch to adaptive pruning. The adaptive threshold should be the main factor controlling pruning, with the rigid threshold acting as a safeguard that does not take over too often.  This may seem complicated, but the rules are actually quite simple, and you may just disregard the definition of T. The usefulness of these options will vary. If you want to speed up **mcl**, try it out and add **--adapt** to your settings. |
|  |  |  |
| **-v** **pruning** | | |
|  |  | Pruning verbosity mode causes **mcl** to emit several statistics related to the pruning process, each of which is described below. Use **-v** **explain** to get explanatory headers in the output as well (or simply use **-v** **all**). **Selection and recovery**  The number of selections and recoveries **mcl** had to perform during each iteration is shown. It also shows the number of vectors for which the mass after final pruning was below the fraction defined by the **-pct** option as a percentage (default probably 90 or 95).  **Initial and pruned vector footprint distributions**  The distribution of the vector footprints (i.e. the number of nonzero entries) before and after pruning is shown. This is assembled in a terse (horrid if you will) format, looking as follows (with some context stripped, noting that the data for three expansion steps is shown):   ``` ----------------------------------------------------  mass percentages  \| distr of vec footprints       \|          \|         \|____ expand ___.____ prune ____\|   prune  \| final   \|e4   e3   e2   \|e4  e3   e2    \| all ny nx\|all ny nx\|8532c8532c8532c\|8532c8532c8532c\| ---------.---------.---------------.---------.-----.  98 88 86  98 91 86 _________022456 ___________0234  98 89 86  98 94 91 _______00245678 ___________0234  98 90 89  99 95 94 _______00235568 ___________0234  ... ```   This particular output was generated (and truncated after three rounds of expansion and inflation) from clustering a protein graph on 9058 nodes with settings **-I** **1.4**, **-P** **2000**, **-S** **500**, **-R** **600**, and **-pct** **95**.  The header entries 8532c85.. indicate thresholds going from 80000, 50000, 20000, 12500, 8000, all the way down to 300, 200, and 125. The character 'c' signifies the base 1.25 (for no apparent reason). The second entry '2' (after '0') on the first line signifies that roughly 20 percent of all the vectors had footprint (#nonzero entries) between 800 and 1250. Likewise, 40 percent had footprint between 300 and 500. The '0' entries signify a fraction somewhere below 5 percent, and the '@' entries signify a fraction somewhere above 95 percent.  Two columns are listed, one for the expansion vector footprints (i.e. after squaring), and the other for the vector footprints *right after initial pruning took place* (i.e. before selection and recovery, after either adaptive or rigid pruning). This may give an idea of the soundness of the initial pruning process (overly severe, or overly mild), and the extent to which you want to apply selection and/or recovery.  **Initial and final mass windows**  The mass averages of the pruned vectors after the first selection stage are shown, and the mass averages of the vectors as *finally computed*, i.e. after selection and recovery. Note that the latter corresponds to a different stage than what is shown for the vector footprints, if either selection or recovery is turned on. For both cases, three averages are shown: the average over all vectors, the average over the worst x cases, and the average over the worst y cases. The mass averages are shown as percentages: '98' on the first line under the 'prune/all' column means that overall 98 percent of the stochastic mass of the matrix was kept after pruning.  This example demonstrates that many entries could be removed while retaining much of the stochastic mass. The effect of the recovery (**-R**) parameter is also clear: the final averages are higher than the initial averages, as a result of **mcl** undoing some overenthousiastic pruning.  An average over the worst k cases is called a window of width k; internally, **mcl** tracks many more such windows. The result of this can be seen when using the **--append-log**=**y** option (which appends a log to the cluster output) or the **--show-log**=**y** option (which sends the log to STDOUT). From a fixed set of windows those that are applicable are tracked, that is, all those windows for which the width does not exceed the graph cardinality. The windows in the fixed set have respective sizes 1, 2, 5, 10, 20, 50, and so on up until 5000000 (which makes 15 windows in all). |
|  |  |  |
| **-nx** i (*x window index*) | | |
| **-ny** j (*y window index*) | | |
|  |  | The options **-nx** and **-ny** both take an index in the range 1..15. The default values for **-nx** and **-ny** are respectively 4 and 7, denoting the fourth and seventh window of respective widths 10 and 100. They are used in the verbosity output as described above. |
|  |  |  |
| **-nj** i (*jury window index*) | | |
|  |  | The **-nj** denotes a window index in the same way as **-nx** and **-ny** do. This particular window is used for computing the *jury marks*, which are the three number reported by **mcl** when it is done. They are a reminder of the existence of pruning and its importance for both speed and accuracy, and they are *indicative rather than authorative*. These jury marks are simply the respective mass averages in the jury window for the first three iterations. The marks are even further simplified and mapped to the jury synopsis, which is a single grade expressed as an adjective. The grades are, in decreasing order of achievement, *perfect exceptional superior excellent good acceptable mediocre poor bad lousy miserable awful wretched atrocious*. Doing 'mcl --jury-charter' will tell you how the jury marks map onto the jury synopsis.  The jury marks should preferably be higher than 70. If they are in the vicinity of 80 or 90, **mcl** is doing fine as far as pruning is concerned. Choose a higher scheme if you think them too low. For very dense graphs that do have strong cluster structure, the jury marks can sink as low as to the 30's and 40's, but the clusterings generated by mcl may still be good. The marks and the synopsis grade the severity of pruning, not cluster quality. Note that the jury becomes friendlier, resp. harsher when the **-nj** option is increased/decreased. |
|  |  |  |
| **-nw** w (*nr of windows*) | | |
|  |  | Normally, **mcl** will use all windows that have width smaller than the cardinality of the input graph. This option limits the set of windows to those w windows of smallest width. This affects the output when setting **--append-log**=**y** output. |
|  |  |  |
| **-nl** l (*number of iterations*) | | |
|  |  | By default, **mcl** will log the window mass averages for the first ten iterations. This options sets that number to **l**. It affects the **--append-log**=**y** output. |

## PIPELINES

As of the 1.006 release, label data can be directly streamed into
MCL as described in GETTING STARTED and EXAMPLES.
For BLAST input this is achieved
by hooking up mcxdeblast **--abc-out**=*-* with
**mcl** - **--abc**. Refer to GETTING STARTED for examples.

The classic mode of operation is more heavyweight, and the remainder
of this section describes the underlying design.
Much of the code now used for streaming directly into mcl was
derived from this earlier framework.

In general, clustering requires several stages; creating the matrix,
running mcl, and displaying the result. The display stage is supported
by clmformat. The matrix creation stage often needs only be
done once for a given data collection, followed by repeated runs of
the other two stages for varying inflation values and scheme settings.

The matrix creation stage can often be split up in two more stages,
namely parsing a data file in some given format, and assembling a
matrix from the data bits and pieces, such as node indices and edge
weights or even edge weight contributions.
The assembly step can be done by mcxassemble,
which allows a very general input format and customizable behaviour
in how the bits and pieces should be transformed to the input graph.
This leaves the parse stage to be filled in.

The mclpipeline script implements a generic and
customizable pipeline encapsulating the four stages distinguished
here (parsing, assembly, clustering, display). It is possible to
let only part of the pipeline be active, and many other features are
supported. The IO mechanism is entirely file based, and files are
associated with parametrizations via file name extensions (by all
means a simple mechanism).

mclpipeline requires a single parse script to be specified.
It will be plugged into the pipeline and you should be set to run.
The parse script must satisfy the interface requirements described
in the mclpipeline manual page.

For BLAST input, the mclblastline script
provides a dedicated mclpipeline interface. It uses the
mcxdeblast script that comes prepackaged with mcl.

## EXAMPLES

The following is an example of label input

```
---8<------8<------8<------8<------8<---
cat hat  0.2
hat bat  0.16
bat cat  1.0
bat bit  0.125
bit fit  0.25
fit hit  0.5
hit bit  0.16
--->8------>8------>8------>8------>8---
```

It can be clustered like this:

```
mcl cathat --abc -o out.cathat
```

The file out.cathat should now like like this

```
---8<------8<------8<------8<------8<---
cat hat bat
bit fit hit
--->8------>8------>8------>8------>8---
```

A few things to note. First, MCL will symmetrize any
arrow it finds. If it sees bat cat 1.0 it will act as if it also
saw cat bat 1.0. You can explicitly specify cat bat 1.0,
mcl will in the first parse stage simply end up with duplicate
entries. Second, MCL deduplicates repeated edges by taking the
one with the maximum value. So,

```
---8<------8<------8<------8<------8<---
cat hat  0.2
hat cat  0.16
hat cat  0.8
--->8------>8------>8------>8------>8---
```

Will result in two arrows cat-hat and hat-cat both
with value 0.8.

## APPLICABILITY

**mcl** will work very well for graphs in which the diameter of the natural
clusters is not too large. The presence of many edges between different
clusters is not problematic; as long as there is cluster structure, mcl
will find it. It is less likely to work well for graphs with clusters
(inducing subgraphs) of large diameter, e.g. grid-like graphs derived from
Euclidean data. So mcl in its canonical form is certainly not fit for
boundary detection or image segmentation. I experimented with a modified
mcl and boundary detection in the thesis pointed to below (see
REFERENCES). This was fun and not entirely unsuccesful, but not
something to be pursued further.

**mcl** likes *undirected input graphs best*, and it really dislikes graphs
with node pairs (i,j) for which an arc going from i to j is present and the
counter-arc from j to i is absent. Try to make your input graph undirected.
Furthermore, mcl interprets edge weights in graphs as similarities. If you
are used to working with dissimilarities, you will have to convert those to
similarities using some conversion formula. The most important thing is
that you feel confident that the similarities are reasonable, i.e. if X is
similar to Y with weight 2, and X is similar to Z with weight 200, then this
should mean that the similarity of Y (to X) is neglectible compared with the
similarity of Z (to X).

**mcl** is probably not suited for clustering *tree graphs*. This is because
mcl works best if there are multiple paths between different nodes in the
natural clusters, but in tree graphs there is only one path between any pair
of nodes. Trees are too sparse a structure for mcl to work on.

**mcl** may well be suited for clustering *lattices*. It will depend
on the density characteristics of the lattice, and the conditions for
success are the same as those for clustering graphs in general: The
diameter of the natural clusters should not be too large.
**NOTE** when clustering a lattice, you *have* to cluster
the underlying undirected graph, and not the directed graph that represents
the lattice itself. The reason is that one has to allow mcl (or any other
cluster algorithm) to 'look back in time', so to speak. Clustering and
directionality bite each other (long discussion omitted).

**mcl** has a worst-case time complexity O(N\*k^2), where N is the number of
nodes in the graph, and k is the maximum number of neighbours tracked during
computations. k depends on the **-P** and **-S** options. If the
**-S** option is used (which is the default setting) then k equals the
value corresponding with this option. Typical values for k are in the range
500..1000. The average case is much better than the worst case though, as
cluster structure itself has the effect of helping mcl's pruning schemes,
certainly if the diameter of natural clusters is not large.

## FILES

There are currently no resource nor configuration files.
The mcl matrix format is described in the mcxio section.

## ENVIRONMENT

MCLXIODIGITS

When writing matrices in interchange format, mcl will use the environment
variable MCLXINTERCHANGEDIGITS (if present) as the precision (number of
digits) for printing the fractional part of values.

MCLXIOVERBOSITY

MCL and its sibling applications will usually report about matrix
input/output from/to disk. The verbosity level can be regulated
via MCLXIOVERBOSITY. These are the levels it can currently be set to.

|  |  |  |
| --- | --- | --- |
| 1 |  | Silent but applications may alter this. |
| 2 |  | Silent and applications can not alter this. |
| 4 |  | Verbose but applications may alter this. |
| 8 |  | Verbose and applications can not alter this (default). |

MCLXIOFORMAT

MCL and its sibling applications will by default output matrices
in interchange format rather than binary format (cf. mcxio).
The desired format can be controlled via the variable
MCLXIOFORMAT. These are the levels it can currently be set to.

|  |  |  |
| --- | --- | --- |
| 1 |  | Interchange format but applications may alter this. |
| 2 |  | Interchange format and applications can not alter this (default). |
| 4 |  | Binary format but applications may alter this. |
| 8 |  | Binary format and applications can not alter this. |

MCLXICFLAGS

If matrices are output in interchange format, by default empty vectors
will not be listed. Equivalently (during input time),
vectors for which no listing is present are understood to be empty -
note that the *presence* of a vector is established using
the domain information found in the header part.
It is possible to enforce listing of empty vectors by
setting bit '1' in the variable MCLXICFLAGS.

MCLXIOUNCHECKED

MCL and its sibling applications will always check a matrix for consistency
while it is being read. If this variable is set, the consistency check is
omitted. For large graphs the speed up can be considerable. However, if the
input graph is not conforming it will likely crash the application that
is using it.

## DIAGNOSTICS

If **mcl** issues a diagnostic error, it will most likely be
because the input matrix could not be parsed succesfully.
**mcl** tries to be helpful in describing the kind of parse error.
The mcl matrix format is described in the mcxio section.

## BUGS

No known bugs at this time. Please send bug reports to mcl-devel@micans.org.

## AUTHOR

Stijn van Dongen.

## HISTORY/CREDITS

The MCL algorithm was conceived in spring 1996 by the present author.
The first implementation of the MCL algorithm followed that spring
and summer. It was written in Perl and proved the viability of
the algorithm. The implementation described here began its life in
autumn 1997. The first versions of the vital matrix library
were designed jointly by Stijn van Dongen and Annius Groenink in
the period Oktober 1997 - May 1999. The efficient matrix-vector
multiplication routine was written by Annius. This routine is
without significant changes still one of the cornerstones of this
MCL implementation.

Since May 1999 all MCL libraries have seen much development and
redesign by the present author. Matrix-matrix multiplication has been
rewritten several times to take full advantage of the sparseness
properties of the stochastic matrices brought forth by the MCL
algorithm. This mostly concerns the issue of pruning - removal of
small elements in a stochastic column in order to keep matrices
sparse.

Very instructive was that around April 2001 Rob Koopman pointed out
that selecting the k largest elements out of a collection of n is
best done using a min-heap. This was the key to the second major
rewrite (now counting three) of the MCL pruning schemes, resulting in
much faster code, generally producing a more accurate computation of
the MCL process.

In May 2001 Anton Enright initiated the parallellization of the
**mcl** code and threaded inflation. From this example, Stijn threaded
expansion. This was great, as the MCL data structures and operands
(normal matrix multiplication and Hadamard multiplication) just beg
for parallellization.

Onwards.
The January 2003 03-010 release introduced support for sparsely
enumerated (i.e. indices need not be sequential) graphs and matrices, the
result of a major overhaul of the matrix library and most higher layers.
Conceptually, the library now sees matrices as infinite quadrants
of which only finite subsections happen to have nonzero entries.

The June 2003 03-154 release introduced unix-type pipelines for clustering,
including the BLAST parser mcxdeblast and the mclblastline script.
The April 2004 04-105 release revived binary format, which has been a first
class citizen every since.

With the March 2005 05-090 release mcxsubs finally acquired a sane
specification syntax. The November 2005 05-314 release brought the ability
to stream label input directly into mcl. The subsequent release introduced a
transformation language shared by various mcl siblings that allows arbitrary
progressions of transformations to be applied to either stream values or
matrix values.

Joost van Baal set up the mcl CVS tree and packaged mcl for Debian
GNU/Linux. He completely autotooled the sources, so much so that at first I
found it hard to find them back amidst bootstrap, aclocal.m4, depcomp, and
other beauties.

Jan van der Steen shared his elegant mempool code. Philip Lijnzaad gave
useful comments. Philip, Shawn Hoon, Abel Ureta-Vidal,
and Martin Mokrejs sent helpful bug reports.

Abel Ureta-Vidal and Dinakarpandian Deendayal commented on
and contributed to mcxdeblast and mcxassemble.

Tim Hughes contributed several good bug reports for mcxassemble,
mcxdeblast and zoem (a workhorse for clmformat).

## SEE ALSO

mclfaq - Frequently Asked Questions.

mcxio - a description of the mcl matrix format.

There are many more utilities. Consult
mclfamily for an overview of and links to all the documentation
and the utilities in the mcl family.

**mcl** development is discussed on mcl-devel@lists.micans.org,
(subscribtion) information is at
https://lists.micans.org:446/listinfo/mcl-devel , this list is
archived at https://lists.micans.org:446/pipermail/mcl-devel/.

mcl's home at http://micans.org/mcl/.

## REFERENCES

Stijn van Dongen, *Graph Clustering by Flow Simulation*.
PhD thesis, University of Utrecht, May 2000.  
http://www.library.uu.nl/digiarchief/dip/diss/1895620/inhoud.htm

Stijn van Dongen. *A cluster algorithm for graphs*.
Technical Report INS-R0010, National Research Institute for Mathematics and
Computer Science in the Netherlands, Amsterdam, May 2000.  
http://www.cwi.nl/ftp/CWIreports/INS/INS-R0010.ps.Z

Stijn van Dongen. *A stochastic uncoupling process for graphs*.
Technical Report INS-R0011, National Research Institute for Mathematics and
Computer Science in the Netherlands, Amsterdam, May 2000.  
http://www.cwi.nl/ftp/CWIreports/INS/INS-R0011.ps.Z

Stijn van Dongen. *Performance criteria for graph clustering and Markov
cluster experiments*. Technical Report INS-R0012, National Research
Institute for Mathematics and Computer Science in the Netherlands,
Amsterdam, May 2000.  
http://www.cwi.nl/ftp/CWIreports/INS/INS-R0012.ps.Z

Enright A.J., Van Dongen S., Ouzounis C.A.
*An efficient algorithm for large-scale detection of protein families*,
Nucleic Acids Research 30(7):1575-1584 (2002).

## NOTES

This page was generated from **ZOEM** manual macros,
http://micans.org/zoem. Both html and roff pages can be created
from the same source without having to bother with all the usual conversion
problems, while keeping some level of sophistication in the typesetting.
This is the PostScript derived from the zoem troff
output.
