# Supplementary material for: MSOAR 2.0: Incorporating tandem duplications into ortholog assignment based on genome rearrangement
Source: BMC Bioinformatics. 2010 Jan 6;11:10. doi: 10.1186/1471-2105-11-10 (PMC2821317; doi:10.1186/1471-2105-11-10)
Supplement: Additional file 2 — contains the software MSOAR 2.0. [file 1471-2105-11-10-S2.GZ › MSOAR2.0/Programs/mcl-06-058/doc/clmorder.html]

The clmorder manual


27 Feb 2006   
**clmorder**
1.006, 06-058

|  |  |  |
| --- | --- | --- |
| 1. |  | NAME |
| 2. |  | SYNOPSIS |
| 3. |  | DESCRIPTION |
| 4. |  | AUTHOR |
| 5. |  | SEE ALSO |

## NAME

clmorder - reorder indices to represent blocks from different clusterings.

## SYNOPSIS

**clmorder** <file name>+

## DESCRIPTION

Given a set of input clusterings, **clmorder** reorders indices so that
the ordering represents blocks from different clusterings (which are
preferably more or less hierarchically organized). It puts nodes that
share many clusters over the different clusters nearby, and puts nodes
in larger clusters earlier in the ordering. It is presumed that the
clusterings are successive subclusterings, but it need not be strictly
the case - **clmorder** will convert the input clusterings to a strictly
nested sequence.

**clmorder** interprets its arguments as names of files containing
clusterings in mcl format (see mcxio).

## AUTHOR

Stijn van Dongen.

## SEE ALSO

mclfamily for an overview of all the documentation
and the utilities in the mcl family.
