# Supplementary material for: MSOAR 2.0: Incorporating tandem duplications into ortholog assignment based on genome rearrangement
Source: BMC Bioinformatics. 2010 Jan 6;11:10. doi: 10.1186/1471-2105-11-10 (PMC2821317; doi:10.1186/1471-2105-11-10)
Supplement: Additional file 2 — contains the software MSOAR 2.0. [file 1471-2105-11-10-S2.GZ › MSOAR2.0/Programs/mcl-06-058/doc/mcxdump.html]

The mcxdump manual


27 Feb 2006   
**mcxdump**
1.006, 06-058

|  |  |  |
| --- | --- | --- |
| 1. |  | NAME |
| 2. |  | SYNOPSIS |
| 3. |  | DESCRIPTION |
| 4. |  | OPTIONS |
| 5. |  | AUTHOR |
| 6. |  | SEE ALSO |

## NAME

mcxdump - dump matrices, optionally map indices to labels

## SYNOPSIS

**mcxdump**
**-imx** <fname> (*matrix file*)
**[-o** <fname> (*dumpf file ('-' for stdout)*)**]**
**[-tab** <fname> (*row/column tab (label) file*)**]**
**[-tabc** <fname> (*column tab file*)**]**
**[-tabr** <fname> (*row tab file*)**]**
**[--lazy-tab** (*allow tab/domain mismatch*)**]**
**[--transpose** (*work with the transpose*)**]**
**[--no-values** (*omit values*)**]**
**[--no-loops** (*omit loops*)**]**
**[--force-loops** (*force loops*)**]**
**[--dump-pairs** (*emit pairs per line*)**]**
**[--dump-lines** (*emit rows per line*)**]**
**[--dump-rlines** (*omit leading column node*)**]**
**[--dump-tabc** (*dump tab file on column domain*)**]**
**[--dump-tabr** (*dump tab file on row domain*)**]**
**[-dump** <fname> (*alias for -o*)**]**
**[-sep-value** <str> (*node/value separator*)**]**
**[-sep-field** <str> (*field separator*)**]**
**[-sep-lead** <str> (*lead separator*)**]**
**[-h** (*list help*)**]**
**[--apropos** (*list synopsis of all options*)**]**


## DESCRIPTION

**mcxdump** reads a data file satisfying the mcl input format
(refer to mcxio). It outputs a line-based format. The
**--dump-pairs** option yields a single matrix entry per line,
identified by the respective column and row identifiers (either index or
label) separated by the field separator.
The **--dump-lines** and **--dump-rlines** result in the
joining of all row entries on a single line, separated by the field
separator. For both formats, the matrix value corresponding with
a particular entry is by default output as well.

## OPTIONS

|  |  |  |
| --- | --- | --- |
| **-imx** <fname> (*matrix file*) | | |
|  |  | Input matrix. |
|  |  |  |
| **-dump** <fname> (*dumpf file ('-' for stdout)*) | | |
|  |  | Output stream. |
|  |  |  |
| **-tab** <fname> (*row/column tab (label) file*) | | |
|  |  | Substitute column indices and row indices by labels from the tab file. Since the same tab file is used for both, this implies that the matrix domains are identical. |
|  |  |  |
| **-tabc** <fname> (*column tab file*) | | |
|  |  | Substitute column indices by labels from the tab file. |
|  |  |  |
| **-tabr** <fname> (*row tab file*) | | |
|  |  | Substitute row indices by labels from the tab file. |
|  |  |  |
| **--lazy-tab** (*allow tab/domain mismatch*) | | |
|  |  | If used, the tab file domain(s) do not necessarily need to match the corresponding domain in the input matrix. Entries missing in the tab files will be replaced by a question mark. |
|  |  |  |
| **--no-values** (*omit values*) | | |
|  |  | Do not emit values. |
|  |  |  |
| **--no-loops** (*omit loops*) | | |
|  |  | Do not output entries for which the row index equals the column index, if present. Applies only to matrices for which column and row domains are equal. |
|  |  |  |
| **--force-loops** (*force loops*) | | |
|  |  | For each column, force output of a row entry that matches the column index. Applies only to matrices for which column and row domains are equal. |
|  |  |  |
| **--dump-pairs** (*emit pairs per line*) | | |
|  |  | Output a single pair of column-identifier and row-identifier per line, optionally followed by the value of the corresponding matrix entry. All fields are separated by the field separator. |
|  |  |  |
| **--dump-lines** (*emit rows per line*) | | |
|  |  | For each matrix column, the corresponding row is output on a single line, with row identifiers separated by the field separator and values attached to the row identifier by the node/value separator. In this format, the column identifier is output as the leading field. |
|  |  |  |
| **--dump-rlines** (*omit leading column node*) | | |
|  |  | As the option above, except that the column identifier is not output. |
|  |  |  |
| **--dump-tabc** (*dump tab file on column domain*) | | |
|  |  | This will only infer the domains from the input matrix. It assumes the input tab file envelopes the matrix column domain, and it outputs a new tab file restricted to that domain. |
|  |  |  |
| **--dump-tabr** (*dump tab file on row domain*) | | |
|  |  | This will only infer the domains from the input matrix. It assumes the input tab file envelopes the matrix row domain, and it outputs a new tab file restricted to that domain. |
|  |  |  |
| **-sep-value** <str> (*node/value separator*) | | |
|  |  | Set the node/value separator for line based row ensemble output. |
|  |  |  |
| **-sep-field** <str> (*field separator*) | | |
|  |  | Set the field separator for different row indices in a given column. |
|  |  |  |
| **-sep-lead** <str> (*lead separator*) | | |
|  |  | Set the lead separator. In the **--dump-lines** format it separates the leading column index from the following ensembl of row indices. It can be useful to make this different from the field separator. One can for example grep for columns that have more than one entry in a matrix mapping nodes to clusters. This will find nodes in overlap. |
|  |  |  |
| **-h** (*list help*) | | |
|  |  | Print help, same as **--apropos**. |
|  |  |  |
| **--apropos** (*list synopsis of all options*) | | |
|  |  | List a one-line synopsis for all options. |

## AUTHOR

Stijn van Dongen.

## SEE ALSO

mcxload,
mcl,
mclfaq,
and mclfamily for an overview of all the documentation
and the utilities in the mcl family.
