# Supplementary material for: MSOAR 2.0: Incorporating tandem duplications into ortholog assignment based on genome rearrangement
Source: BMC Bioinformatics. 2010 Jan 6;11:10. doi: 10.1186/1471-2105-11-10 (PMC2821317; doi:10.1186/1471-2105-11-10)
Supplement: Additional file 2 — contains the software MSOAR 2.0. [file 1471-2105-11-10-S2.GZ › MSOAR2.0/Programs/mcl-06-058/doc/mcxmap.html]

The mcxmap manual


27 Feb 2006   
**mcxmap**
1.006, 06-058

|  |  |  |
| --- | --- | --- |
| 1. |  | NAME |
| 2. |  | SYNOPSIS |
| 3. |  | DESCRIPTION |
| 4. |  | OPTIONS |
| 5. |  | EXAMPLES |
| 6. |  | AUTHOR |
| 7. |  | SEE ALSO |

## NAME

mcxmap - permute or remap the indices of graphs and matrices.

## SYNOPSIS

**mcxmap**
**[-cmul** a (*coefficient*)**]**
**[-cshift** b (*translate*)**]**
**[-rmul** c (*coefficient*)**]**
**[-rshift** d (*translate*)**]**
**[-mul** e (*coefficient*)**]**
**[-shift** f (*translate*)**]**
**[-map** fname (*row/col map file*)**]**
**[-rmap** fname (*row map file*)**]**
**[-cmap** fname (*column map file*)**]**
**[--invert** (*invert map file(s)*)**]**
**[--invertc** (*invert column map file*)**]**
**[--invertr** (*invert row map file*)**]**
**[-digits** n (*digits width*)**]**
**[-o** fname (*output*)**]**
**-imx** fname (*input*)


## DESCRIPTION

This utility relabels graphs or matrices. Its main use is in applying a
*map file* to a given matrix or graph. A map file contains a so called
*map matrix* in mcl format that has some special properties (given
further below). The functionality of mcxmap can also be provided by **mcx**, as
a mapped matrix (i.e. the result of applying a map matrix to another matrix)
is simply the usual matrix product of a matrix and a map matrix. However,
**mcx** will construct a new matrix and leave the original matrix to be mapped
alone. When dealing with huge matrices, considerable gains in efficiency
memory-wise and time-wise can be achieved by doing the mapping in-place.
This is what mcxmap does. In the future, its functionality may be
embedded in **mcx** with new mcx operators.

The special properties of a map matrix are

|  |  |  |
| --- | --- | --- |
| \* |  | The column domain and row domain are of the same cardinality. |
|  |  |  |
| \* |  | Each column has exactly one entry. |
|  |  |  |
| \* |  | Each row domain index occurs in exactly one column. |

These properties imply that the matrix can be used
as a map from the column domain onto the row domain.
An example map matrix is found in the EXAMPLES Section.

## OPTIONS

|  |  |  |
| --- | --- | --- |
| **-o** fname (*output file*) | | |
|  |  | Output file. |
|  |  |  |
| **-imx** fname (*input file*) | | |
|  |  | Input file. |
|  |  |  |
| **-digits** n (*digits width*) | | |
|  |  | Number of digits output for values. |
|  |  |  |
| **-cmul** a (*coefficient*) | | |
| **-cshift** b (*translate*) | | |
| **-cmap** fname (*col map file*) | | |
|  |  | If the map file is specified, column indices will be mapped according to this file. Otherwise, if any of the first two options is used, column indices i are mapped to a\*i+b. |
|  |  |  |
| **--invert** (*invert map file(s)*) | | |
| **--invertc** (*invert column map file*) | | |
| **--invertr** (*invert row map file*) | | |
|  |  | Specifies to use the inverse of the map file(s) given. |
|  |  |  |
| **-rmul** c (*coefficient*) | | |
| **-rshift** d (*translate*) | | |
| **-rmap** fname (*row map file*) | | |
|  |  | If the map file is specified, row indices will be mapped according to this file. Otherwise, if any of the first two options is used, row indices i are mapped to c\*i+d. |
|  |  |  |
| **-mul** e (*coefficient*) | | |
| **-shift** f (*translate*) | | |
| **-map** fname (*row/col map file*) | | |
|  |  | If the map file is specified, both column and row indices will be mapped according to it. Otherwise, if any of the two first options is specified, both column and row indices i will be mapped to e\*i+f. |

## EXAMPLES

The matrix below has two canonical domains which are identical.
It denotes a map of the canonical domain onto itself, in which
node 0 is relabeled to 8, node 1 is relabeled to 5, et cetera.

```
(mclheader
mcltype matrix
dimensions 12x12
)
(mclmatrix
begin
0  8  $
1  5  $
2  3  $
3  2  $
4  4  $
5  6  $
6  7  $
7  9  $
8  1  $
9  10 $
10 11 $
11 0  $
)
```

## AUTHOR

Stijn van Dongen.

## SEE ALSO

mcxio,
mcx,
mcxsubs,
and mclfamily for an overview of all the documentation
and the utilities in the mcl family.
