# Supplementary material for: MSOAR 2.0: Incorporating tandem duplications into ortholog assignment based on genome rearrangement
Source: BMC Bioinformatics. 2010 Jan 6;11:10. doi: 10.1186/1471-2105-11-10 (PMC2821317; doi:10.1186/1471-2105-11-10)
Supplement: Additional file 2 — contains the software MSOAR 2.0. [file 1471-2105-11-10-S2.GZ › MSOAR2.0/Programs/mcl-06-058/doc/clmformat.html]

The clmformat manual


27 Feb 2006   
**clmformat**
1.006, 06-058

|  |  |  |
| --- | --- | --- |
| 2. |  | SYNOPSIS |
| 3. |  | DESCRIPTION |
| 4. |  | OPTIONS |
| 5. |  | OUTPUT EXPLAINED |
| 6. |  | AUTHOR |
| 7. |  | REFERENCES |
| 8. |  | SEE ALSO |

## NAME

clmformat - display cluster results in readable form

(optionally with labels and/or cohesion and stickiness measures
attached).

Unless used with the **-dump** *fname* or **--dump** option,
**clmformat** depends on the presence of the macro processor **zoem**, as
described further below.

The **-icl** *fname* input clustering option is always required. The
**-imx** *fname* input matrix option is required in fancy mode. The tab
file option **-tab** *fname* is needed if you want label information in
the output rather than mcl identifiers.

## SYNOPSIS

**clmformat** has two different modes of output: *dump* and *fancy*.
If neither is specified, *fancy* is used. In this mode, clmformat
generates a large arrary of performance measures related to nodes and
clusters in both interlinked html output and plain text files. The files
will be contained in an output directory that is newly created if not yet
existing. In fancy mode the **-imx** option is required and the macro
processor **zoem** must be available (http://micans.org/zoem).

If *dump* is specified (see below how to do this) clmformat just
generates a dump file where each line
contains a cluster in the form of tab-separated indices, or tab-separated
labels in case the **-tab** option is used. This dump is easy to parse
with a simple or even quick-and-dirty script.
You can include some very simple performance measures in this dump file
by supplying **--dump-measures**. Use
**-dump** *fname* to specify the name of the file to dump to, rather
than having **clmformat** construct a file name by itself.

**clmformat** can combine the both modes by using either **--dump** or
**-dump** *fname* *and* **--fancy**. In this case the dump file
will be created in the output directory that is used by *fancy* mode.

**clmformat**

**-icl** fname (*input cluster file*)  
**-imx** fname (*input matrix/graph file*)  
**-pi** num (*apply pre-inflation to matrix*)  
**-tab** fname (*read tab file*)  
**--lazy-tab** (*allow mismatched tab-file*)  
**--dump** (*write dump to dump.<icl-name>*)  
**-dump** fname (*write dump to file*)  
**--dump-pairs** (*write cluster/node pair per line*)  
**--dump-measures** (*write simple performance measures*)  
**-dump-node-sep** str (*separate entries with str*)  
**--fancy** (*spawn information blizzard*)  
**-dir** dirname (*write results to directory*)  
**-infix** str (*use after base name/directory*)  
**-nsm** fname (*output node stickiness file*)  
**-ccm** fname (*output cluster cohesion file*)  
**--adapt** (*allow domain mismatch*)  
**--subgraph** (*take subgraph with --adapt*)  
**-zmm** fname (*assume macro definitions are in fname*)  
**-fmt** fname (*write to encoding file fname*)  
**--version** (*print version*)  
**-h** (*synopsis*)

Consult the option descriptions and the introduction above for
interdependencies of options.

**clmformat** generates in fancy mode a logical description of the
to-be-formatted content in a very small vocabulary of clmformat-specific
zoem macros. The appearance of the output can be easily changed by adapting
a zoem macro definition file (also output by clmformat) that is used by the
zoem interpreter to interpret the logical elements.

The output format is apt to change over subsequent releases, as a result of
user feedback. Such changes will most likely be confined to the zoem macro
definition file.

The OUTPUT EXPLAINED section further below is likely to be of interest.

## DESCRIPTION

The primary function of **clmformat** is to display cluster results and
associated confidence measures in a readable form, by listing clusters in
terms of the labels associated with the indices that are used in the mcl
matrix. The labels must be stored in a so called *tab* file; see the
**-tab** option for more information.

**NOTE**  
**clmformat** output is in the form of *zoem* macros.
You need to have zoem installed in your system if you want clmformat
to be of use. Zoem will not be necessary if you are using
the **-dump** option.

The **-imx** *mx* option is required
unless the **-dump** option is used. The latter option
results in special behaviour described under the
**-dump** *fname* entry.

Output is by default written in a directory that
is newly created if it does not yet exist (normally several files
will be created, for which the directory acts as a natural container).
It is possible to simply output to the current directory, for that you need
to specify **-dir** **./**. If **-dir** is not specified, the output
directory fmt.<clname> will be used, where <clname> is the argument
to the **-cl** option. In the output directory, clmformat will
normally write two files. One contains zoem macros encoding formatted output
(the encoding file), and the second (the definition file) contains zoem
macro definitions which are used by the former.

The encoding file is by default called fmt.azm
(cf. the **-fmt** *fname* option).
It contains *zoem* macros. It imports the macro definition file
called clmformat.zmm
that is normally also written by clmformat. Another macro definition
file can be specified by using the **-zmm** *<defsname>*
option. In this case clmformat will refrain from writing the definition
file and replace mentions of clmformat.zmm in the encoding file
by <defsname>.

The encoding file needs to be processed by issuing one of the following
commands from within the directory where the file is located.

```
   zoem -i fmt -d html
   zoem -i fmt -d txt
```

The first will result in HTML formatted output, the second in
plain text format. Obviously, you need to have installed zoem
(e.g. from http://micans.org/zoem/src/) for this to work.

For each cluster a paragraph is output. First comes a listing of other
clusters (in order of relevance, possibly empty) for which a significant
amount of edges exists between the other and the current cluster. Second
comes a listing of the nodes in the current cluster. For each node a small
sublist is made (in order of relevance, possibly empty) of other clusters in
which the node has neighbours and for which the total sum of corresponding
edge weights is significant.
Several quantities are output for each node/cluster pair that is
deemed relevant. These are explained in the section OUTPUT EXPLAINED.

Clusters will by default be output to file until the total node count has
exceeded a threshold (refer to the **-lump-count**
option).

**clmformat** also shows how well each node fits in the cluster it is in and
how cohesive each cluster is, using simple but effective measures
(described in section OUTPUT EXPLAINED).
This enables you to compare the quality of the clusters in a clustering
relative to each other, and may help in identifying both interesting areas
and areas for which cluster structure is hard to find or perhaps absent.

## OPTIONS

|  |  |  |
| --- | --- | --- |
| **-icl** fname (*input cluster file*) | | |
|  |  | Name of the clustering file. |
|  |  |  |
| **-imx** fname (*input matrix/graph file*) | | |
|  |  | Name of the graph/matrix file. |
|  |  |  |
| **-tab** fname (*read tab file*) | | |
|  |  | The file fname should be in *tab format*. Refer to mcxio. |
|  |  |  |
| **--lazy-tab** (*allow mismatched tab-file*) | | |
|  |  | Allow missing and spurious entries in the tab file. |
|  |  |  |
| **-dump** fname (*write dump to file*) | | |
|  |  | Clusters are written to file. For each cluster a single line is written containing all indices of all nodes in that cluster. The indices are separated by tabs. If a tab file is specified, the indices are replaced by the corresponding tab file entry. |
|  |  |  |
| **--dump** (*write dump to file*) | | |
|  |  | As **-dump** *fname* except that **clmformat** writes to the file named dump.<icl-name> where <icl-name> is the argument to the **-icl** option. |
|  |  |  |
| **--fancy** (*force fancy mode*) | | |
|  |  | This enforces fany mode if either of **-dump** or **--dump** is given. The dump file will be created in the output directory. |
|  |  |  |
| **--dump-pairs** (*write cluster/node pair per line*) | | |
|  |  | Rather than writing a single cluster on each line, write a single cluster index/node (either tab entry or index) pair per line. Works in conjunction with the **-tab** and **-imx** options. |
|  |  |  |
| **--dump-measures** (*write simple performance measures*) | | |
|  |  | If an input matrix is specified with **-imx** *fname*, three measures of efficiency are prepended, respectively the simple projection score, efficiency or coverage, and the max-efficiency or max-coverage. |
|  |  |  |
| **-dump-node-sep** str (*separate entries with str*) | | |
|  |  | Separate entries in the dump file with **str**. |
|  |  |  |
| **-pi** num (*apply pre-inflation to matrix*) | | |
|  |  | Apply pre-inflation to the matrix specified with the **-imx** option. This will cause the efficiency scores to place a higher reward on high-weight edges being covered by a clustering (assuming that *num* is larger than one). This option is also useful when **mcl** itself was instructed to use pre-inflation when clustering a graph. |
|  |  |  |
| **-lump-count** n (*node threshold*) | | |
|  |  | The zoem file is created such that during zoem processing clusters are formatted and output within a single file until the node threshold has been exceeded. A new file is then opened and the procedure repeats itself. |
|  |  |  |
| **--adapt** (*allow domain mismatch*) | | |
|  |  | Allow the cluster domain to differ from the graph domain. Presumably the clustering is a clustering of a subgraph. The cohesion and stickiness measures will pertain to the relevant part of the graph only. |
|  |  |  |
| **--subgraph** (*use restriction*) | | |
|  |  | If the cluster domain is a subset of the graph domain, the cohesion and stickiness measures will by default still pertain to the entire graph. By setting this option, the measures will pertain to the subgraph induced by the cluster domain. |
|  |  |  |
| **-dir** dirname (*write results to directory*) | | |
|  |  | Use **dirname** as output directory. It will be created if it does not exist already. |
|  |  |  |
| **-fmt** fname (*write to encoding file fname*) | | |
|  |  | Write to encoding file **fname** rather than the default fmt.azm. It is best to supply fname with the standard zoem suffix .azm. Zoem will process file of any name, but those lacking the .azm suffix must be specified using the zoem **-I** *fname* option. |
|  |  |  |
| **-zmm** defsname (*assume macro definitions are in fname*) | | |
|  |  | If this option is used, clmformat will not output the definition file, and mentions of the definition file in the encoding file will use the file name defsname. This option assumes that a valid definition file by the name of defsname does exist. |
|  |  |  |
| **-nsm** fname (*output node stickiness file*) | | |
|  |  | This option specifies the name in which to store (optionally) the **node stickiness matrix**. It has the following structure. The columns range over all elements in the graph as specified by the **-imx** option. The rows range over the clusters as specified by the **-icl** option. The entries contain the projection value of that particular node onto that particular clusters, i.e. the sum of the weights of all arcs going out from the node to some node in that cluster, written as a fraction relative to the sum of weights of all outgoing arcs. |
|  |  |  |
| **-ccm** fname (*output cluster cohesion file*) | | |
|  |  | This option specifies the name of the file in which to store (optionally) the **cluster cohesion matrix**. It has the following structure. Both columns and rows range over all clusters in the clustering as specified by the **-icl** option. An entry specifies the projection of one cluster onto another cluster, which is simply the average of the projection value onto the second cluster of all nodes in the first cluster. |
|  |  |  |
| **--version** (*write version*) | | |
|  |  | Write version. Really. |

## OUTPUT EXPLAINED

What follows is an explanation of the output provided by the
standard zoem macros. The output comes in a pretty terse number-packed
format. The decision was made not to include headers and captions
in the output in order to keep it readable.
You might want to print out the following annotated examples.
At the same side of the equation, the following is probably tough
reading unless you have an actual example of clmformatted output at hand.

Below mention is made of the projection value for a node/cluster pair.
This is simply the total amount of edge weights for that node
in that cluster (corresponding to neighbours of the node in the
cluster) relative to the overall amount of edge weights for that node
(corresponding to all its neighbours).
The coverage measure (refered to as **cov**)
is also used. This is similar to the projection
value, except that a) the coverage measure rewards the inclusion
of large edge weights (and penalizes the inclusion of insignificant
edge weights) and b) rewards node/cluster pairs for which the neighbour set
of the node is very similar to the cluster.
The maximum coverage measure (refered to as **maxcov**) is similar
to the normal coverage measure except that it rewards inclusion
of large edge weights even more.
The cov and maxcov performance measures have several nice continuity and
monotonicity properties and are described in [1].

**Example cluster header**  

```
Cluster 0 sz 15 self 0.82 cov 0.43-0.26
   10: 0.11
   18: 0.05
   12: 0.02
```

**explanation**  

```
Cluster 0 sz 15 self 0.82 cov 0.43-0.26
        |    |       |           | |
        clid count   proj      cov covmax

   10: 0.11
    |  |
clidx1 projx1

   18: 0.05
    |  |
clidx2 projx2

clid    Numeric cluster identifier (arbitrarily) assigned by MCL.
count   The size of cluster clid.
proj    Projection value for cluster clid [d].
cov     Coverage measure for cluster clid [d].
maxcov  Max-coverage measure for cluster clid [d].
clidx1  Index of other cluster sharing relatively many edges.
projx1  Projection value for the clid/clidx1 pair of clusters [e].
clidx2  :
projx2  : as clidx1 and projx1
```

**Example inner node**  
An inner node is listed under a cluster, and it is simply a member of that
cluster. The name is as opposed to 'outer node', described below.

```
[foo bar zut]
    21     7-5      0.73 0.420-0.331  0.282-0.047  0.071-0.035 <3.54>
      10   6/3      0.16 0.071-0.047  0.268-0.442 
      12   4/2      0.11 0.071-0.035  0.296-0.515
```

**explanation**  

```
[label]
    21     7-5      0.73 0.420-0.331  0.282-0.047  0.071-0.035 <3.54>
     |     | |      |        | |          | |          | |     |
    idx  nbi nbo    proj   cov covmax max_i min_i  max_o-min_o SUM

      10   6/3      0.16 0.268-0.442  0.071-0.047
       |   | |      |        | |          | |
  clusid  sz nb     proj   cov covmax max_i min_i

label   Optional; with -tab <tabfile> option.
idx     Numeric (mcl) identifier.
nbi     Count of the neighbours of node idx within its cluster.
nbo     Count of the neighbours of node idx outside its cluster.
proj    Projection value [a] of nbi edges.
cov     Skewed projection [b], rewards inclusion of large edge weights.
covmax  As cov above, rewarding large edge weights even more.
max_i   Largest edge weight in the nbi set, normalized [c].
min_i   Smallest edge weight in the nbi set [c].
max_o   Largest edge weight outside the nbi set [c]
min_o   Smallest edge weight outside the nbi set [c].
SUM     The sum of all edges leaving node idx.

clusid  Index of other cluster that is relevant for node idx.
sz      Size of that cluster.
nb      Count of neighbours of node idx in cluster clusid.
proj    Projection value of edges from node idx to cluster clusid.
cov     Skewed projection of edges from node idx to cluster clusid.
covmax  Maximally skewed projection, as above.
max_o   Largest edge weight for node idx to cluster clusid [c].
min_o   Smallest edge weight for  node idx to cluster clusid [c].
```

**Example outer node**  
An outer node is listed under a cluster. The node is not part of that cluster,
but seems to have substantial connections to that cluster.

```
[zoo eek few]
    29   18#2        2-5      0.65 0.883-0.815  0.436-0.218  0.073-0.055
                      /4      0.27 0.070-0.109  0.073-0.055
```

**explanation**  

```
[label]
    29   18#2        2-5      0.65 0.883-0.815  0.436-0.218  0.073-0.055
    |    |  |        | |      |        | |          | |          | |
    idx  cl sz     nbi nbo    proj   cov maxcov max_i min_i  max_o min_o
         id
                      /4      0.27 0.070-0.109  0.073-0.055  <2.29>
                       |      |        | |          | |      |
                       nb     proj   cov maxcov max_i min_i  SUM

label   Optional; with -tab <tabfile> option.
idx     Numeric (mcl) identifier
clid    Index of the cluster that node idx belongs to
sz      Size of the cluster that node idx belongs to
proj    :
cov     :  All these entries are the same as described above
covmax  :  for inner nodes, pertaining to cluster clid,
max_i   :  i.e. the native cluster for node idx
min_i   :  (it is a member of that cluster).
max_o   :
min_o   :

nb      The count of neighbours of node idx in the current cluster
proj    Projection value for node idx relative to current cluster.
cov     Skewed projection (rewards large edge weights), as above.
covmax  Maximally skewed projection, as above.
max_o   Largest edge weight for node idx in current cluster [c].
min_o   smallest edge weight for node idx in current cluster [c].
SUM     The sum of *all* edges leaving node idx.
```

|  |  |  |
| --- | --- | --- |
| [a] |  | The projection value for a node relative to some subset of its neighbours is the sum of edge weights of all edges to that subset. The sum is witten as a fraction relative to the sum of edge weights of all neighbours. |
|  |  |  |
| [b] |  | cov and covmax stand for coverage and maximal coverage. The coverage measure of a node/cluster pair is a generalized and skewed projection value [a] that rewards the presence of large edge weights in the cluster, relative to the collection of weights of all edges departing from the node. The maxcov measure is a projection value skewed even further, correspondingly rewarding the inclusion of large edge weights. The cov and maxcov performance measures have several nice continuity properties and are described in [1]. |
|  |  |  |
| [c] |  | All edge weights are written as the fraction of the sum SUM of all edge weights of edges leaving node idx. |
|  |  |  |
| [d] |  | For clusters the projection value and the coverage measures are simply the averages of all projection values [a], respectively coverage measures [b], taken over all nodes in the cluster. The cluster projection value simply measures the sum of edge weights internal to the cluster, relative to the total sum of edge weights of all edges where at least one node in the edge is part of the cluster. |
|  |  |  |
| [e] |  | The projection value for start cluster x and end cluster y is the sum of edge weights of edges between x and y as a fraction of the sum of all edge weights of edges leaving x. |

## AUTHOR

Stijn van Dongen.

## REFERENCES

[1]
Stijn van Dongen. *Performance criteria for graph clustering and Markov
cluster experiments*. Technical Report INS-R0012, National Research
Institute for Mathematics and Computer Science in the Netherlands,
Amsterdam, May 2000.  
http://www.cwi.nl/ftp/CWIreports/INS/INS-R0012.ps.Z


## SEE ALSO

mclfamily for an overview of all the documentation
and the utilities in the mcl family.
