# Supplementary material for: MSOAR 2.0: Incorporating tandem duplications into ortholog assignment based on genome rearrangement
Source: BMC Bioinformatics. 2010 Jan 6;11:10. doi: 10.1186/1471-2105-11-10 (PMC2821317; doi:10.1186/1471-2105-11-10)
Supplement: Additional file 2 — contains the software MSOAR 2.0. [file 1471-2105-11-10-S2.GZ › MSOAR2.0/Programs/phylip-3.67/doc/draw.html]

main


version 3.66

# Drawtree and Drawgram

© Copyright 1986-2006 by The University of
Washington. Written by Joseph Felsenstein. Permission is granted to copy
this document provided that no fee is charged for it and that this copyright
notice is not removed.

Drawtree and Drawgram are interactive tree-plotting programs that take a
tree description in a file and read it, and then let you interactively make
various settings and then plot the tree on a laser printer, plotter,
or dot matrix printer. In many cases (with Macintosh or PC graphics,
with X windows, or with a Tektronix-compatible graphics terminal)
you can preview the resulting tree. This allows you to modify the tree
until you like the result, then plot the result. Drawtree plots
unrooted trees and Drawgram plots rooted cladograms and phenograms. On
good laser printers or as files for good drawing programs both can produce
fully publishable
results. On dot matrix printers the results look grainy but are good
enough for overhead transparencies or slides for presentations.

These programs are descended from PLOTGRAM and PLOTREE written by
Christopher Meacham. I have incorporated his code for fonts and his
plotter drivers, and in Drawtree have used some of his code for drawing
unrooted trees. In both programs I have also included some plotter driver
code by David Swofford, Julian Humphries and George D.F. "Buz" Wilson,
to all of whom I am very grateful. Mostly, however, they consist of my own
code and that of my programmers. The font files are printable-character
recodings of the public-domain Hershey fonts, recoded by Christopher Meacham.

This document will describe the features common to both programs. The
documents for Drawtree and Drawgram describe the particular choices you
can make in each of those programs. The Appendix to this documentation file
contains some pieces of C code that can be inserted to make the program
handle another plotting device -- the plotters by Calcomp.

## A Short Introduction

To use Drawtree and Drawgram, you must have

(1): The compiled version of the program. If you have not obtained a version of PHYLIP precompiled for your machine, you will have to take the source code given here and modify it for your C compiler and then compile it. This is not too hard: it is discussed below. (2): A tree file. Trees are described in the nested-parenthesis notation used throughout PHYLIP and standardized in an informal meeting of program authors in Durham, New Hampshire in June, 1986. Trees for both programs may be either bifurcating or multifurcating, and may either have or not have branch lengths. Tree files produced by the PHYLIP programs are in this form. There is further description of the tree file format later in this document. (3): A font file. There are six font files distributed with PHYLIP: these consist of three Roman, two Italic, and one Russian Cyrillic font, all from the public-domain Hershey Fonts, in ASCII readable form. The details of font representation need not concern you; all you need to do is to copy the font file corresponding to the font you want into the appropriate folder under the appropriate file name, and let the program use it. Or you can let the program ask you for the name of the font file, which it will do if it does not find one itself. The six fonts are, respectively, a one- and a two-stroke sans-serif Roman font, a three-stroke serifed Roman font, a two- and a three- stroke serifed Italic font, and a two-stroke Cyrillic font for the Russian language. If this is not clear just try them all. Note that for some printers several built-in fonts such as Times-Roman and Courier can be used too. The Hershey fonts were created by Dr. A. V. Hershey of the U. S. National Bureau of Standards in the late 1960s. They may be freely distributed except that they may not be distributed in the original format used the the U. S. National Technical Information Service. Our format is different from the NTIS one. See Appendix 2, below, if you need a detailed discussion of the format. (4): A plotting device, and if possible a screen on which you can preview the plot. The programs work with Postscript-compatible laser printers, laser printers compatible with the PCL printer language of the Hewlett-Packard Laserjet series, the PICT format for the MacDraw drawing program, the file formats for the freeware X-windows drawing programs xfig and idraw, IBM PC graphics screens, the PCX file format for the PC Paintbrush painting program, the X Bitmap format for X-windows, plotters including Hewlett-Packard models, dot matrix printers including models by Epson and Apple, graphics terminals from DEC and Tektronix, the input format for the freeware ray-tracing (3-dimensional rendering) programs POV and rayshade, and, strangest and most wonderful of all, the Virtual Reality Markup Language (VRML) which is a file format that is used by freely-available virtual reality programs. You can choose the plotting and previewing devices from a menu at run time, and these can be different. There are places in the source code for the program where you can insert code for a new plotter, should you want to do that.

Once you have all these, the programs should be fairly self explanatory,
particular if you can preview your plots so that you can discover the
meaning of the different options by trying them out.

Once you have a compiled version of the appropriate program, say
Drawgram, and a file called (say) intree with the tree in it, and
a font file (say font2 which you have copied as a file called
fontfile),
all you do is run the program Drawgram. It should automatically read the
font and tree files, and will allow you to change the graphics devices. Then
it will let you see the options it has chosen, and ask you if you
want to change these. Once you have modified those that you want to,
you can tell it to accept those. The program will then allow you to
preview the tree on your screen, if you have an appropriate graphics
capability on your computer screen, and if you haven't told it in the menu
not to preview the plot. After
previewing the tree, the program will want to know whether you are ready to
plot the tree. You answer this using the Plot choice in the File menu
will be found in the upper-left corner of he preview window in Windows systems
or X Windows systems such as Linux. In Mac OS X systems it is a choice in
THE Upper-left corner of the preview window. If you have chosen not to
preview the plot, plotting will occur after you close the menu by making the
Y (yes) choice when you are asked whether you want to accept the plot as is.
If you say no, it will once again allow you to change options and
will the allow you to preview the tree again, and so on as many times
as you want. If you say yes, then it will write a file called (say)
plotfile. If you then copy this file to your printer or plotter,
it should result in a beautifully plotted tree. If the final plotting
device is a Macintosh or PC graphics screen, it may not write a plot file
but will plot directly on the screen.

If you don't want to print the file immediately, but want to edit the
figure first, you should have chosen an output format that is readable
by a draw program. Postscript format is readable by drawing programs
such as Adobe Illustrator, Canvas, Freehand, and Coreldraw, and can be
displayed by the Unix utilities Ghostscript and Ghostview. Some
Macintosh drawing programs such as MacDraw can read PICT format.
On Windows systems bitmap drawing editors such as Paint can read
Windows Bitmap files. We have provided output formats here for
Xfig and Idraw drawing programs available on Linux or Unix systems.
Drawing programs can be used to add branch length numbers (something too
hard for us to do automatically in these programs) and to make scale bars.
Another use is as a way of printing out the trees, as most drawing
programs are set up to print out their figures.

Having read the above, you may be ready to run the program. Below you
Will find more information about representation of trees in the tree
file, on the different kinds of graphics devices supported by this
program, and on how to recompile these programs.

## Trees

The Newick Standard for representing trees in computer-readable
form makes use of the correspondence between trees and nested
parentheses, noticed in 1857 by the famous English mathematician Arthur
Cayley. If we have this rooted tree:

```
                         A                 D
                          \         E     /
                           \   C   /     /
                            \  !  /     /
                             \ ! /     /
                        B     \!/     /
                         \     o     /
                          \    !    /
                           \   !   /
                            \  !  /
                             \ ! /
                              \!/
                               o
                               !
                               !
```

then in the tree file it is represented by the following sequence of printable
characters, starting at the beginning of the file:

(B,(A,C,E),D);

The tree ends with a semicolon. Everything after the semicolon in the
input file is ignored, including any other trees. The bottommost node
in the tree is an interior node, not a tip. Interior nodes are
represented by a pair of matched parentheses. Between them are
representations of the nodes that are immediately descended from that
node, separated by commas. In the above tree, the immediate
descendants are B, another interior node, and D. The other interior
node is represented by a pair of parentheses, enclosing representations
of its immediate descendants, A, C, and E.

Tips are represented by their names. A name can be any string of
printable characters except blanks, colons, semcolons, parentheses, and
square brackets. In the programs a maximum of 20 characters are allowed
for names: this limit can easily be increased by recompiling the program
and changing the constant
declaration for "MAXNCH" in phylip.h.

Because you may want to include a blank in a name, it is assumed that
an underscore character ("\_") stands for a blank; any of these in a
name will be converted to a blank when it is read in. Any name may also
be empty: a tree like

(,(,,),);

is allowed. Trees can be multifurcating at any level (while in many
of the programs multifurcations of user-defined trees are not allowed
or restricted to a trifurcation at the bottommost level, these programs
do make any such restriction).

Branch lengths can be incorporated into a tree by putting a real
number, with or without decimal point, after a node and preceded by
a colon. This represents the length of the branch immediately
below that node. Thus the above tree might have lengths
represented as:

(B:6.0,(A:5.0,C:3.0,E:4.0):5.0,D:11.0);

These programs will be able to make use of this information only if
lengths exist for every branch, except the one at the bottom of
the tree.

The tree starts on the first line of the file, and can continue to
subsequent lines. It is best to proceed to a new line, if at all,
immediately after a comma. Blanks can be inserted at any point except
in the middle of a species name or a branch length.

The above description is of a subset of the Newick Standard. For
example, interior nodes can have names in that standard, but if
any are included the present programs will omit them.

To help you understand this tree representation, here are some trees
in the above form:

```
((raccoon:19.19959,bear:6.80041):0.84600,((sea_lion:11.99700,
seal:12.00300):7.52973,((monkey:100.85930,cat:47.14069):20.59201,
weasel:18.87953):2.09460):3.87382,dog:25.46154);

(Bovine:0.69395,(Gibbon:0.36079,(Orang:0.33636,(Gorilla:0.17147,(Chimp:0.19268,
Human:0.11927):0.08386):0.06124):0.15057):0.54939,Mouse:1.21460);

(Bovine:0.69395,(Hylobates:0.36079,(Pongo:0.33636,(G._Gorilla:0.17147,
(P._paniscus:0.19268,H._sapiens:0.11927):0.08386):0.06124):0.15057):0.54939,
Rodent:1.21460);

();

((A,B),(C,D));

(Alpha,Beta,Gamma,Delta,,Epsilon,,,);
```

The Newick Standard was adopted June 26, 1986 by an informal
committee meeting during the Society for the Study of Evolution
meetings in Durham, New Hampshire and consisting of James Archie,
William H.E. Day, Wayne Maddison, Christopher Meacham, F. James Rohlf,
David Swofford, and myself. A web page describing it will be found
at 
http://evolution.gs.washington.edu/phylip/newicktree.html.

## Plotter file formats

When the programs run they have a menu which allows you to set (on its
option P) the final plotting device, and another menu which allows you
to set the type of preview screen. The
choices for previewing are a subset of those available for plotting,
and they can be different (the most useful combination will usually be a
previewing graphics screen with a hard-copy plotter or a drawing program
graphics file format).

The plotting device menu looks like this:

```
Which plotter or printer will the tree be drawn on?
(many other brands or models are compatible with these)

   type:       to choose one compatible with:

        L         Postscript printer file format
        M         PICT format (for drawing programs)
        J         HP Laserjet PCL file format
        W         MS-Windows Bitmap
        F         FIG 2.0 drawing program format
        A         Idraw drawing program format
        Z         VRML Virtual Reality Markup Language file
        P         PCX file format (for drawing programs)
        K         TeKtronix 4010 graphics terminal
        X         X Bitmap format
        V         POVRAY 3D rendering program file
        R         Rayshade 3D rendering program file
        H         Hewlett-Packard pen plotter (HPGL file format)
        D         DEC ReGIS graphics (VT240 terminal)
        E         Epson MX-80 dot-matrix printer
        C         Prowriter/Imagewriter dot-matrix printer
        T         Toshiba 24-pin dot-matrix printer
        O         Okidata dot-matrix printer
        B         Houston Instruments plotter
        U         other: one you have inserted code for
 Choose one:
```

Here are the choices, with some comments on each:

**Postscript printer file format.** This means that the program will
generate a file containing Postscript commands as its plot file. This
can be printed on any Postscript-compatible laser printer. The page
size is assumed to be 8.5 by 11 inches, but as plotting is within this
limit A4 metric paper should work well too. This is the best
quality output option. For this printer the menu options in Drawgram
and Drawtree that allow you to select one of the built-in fonts will
work. The programs default to Times-Roman when this plotting option
is in effect. I have been able to use fonts Courier, Times-Roman, and
Helvetica. The others have eluded me for some reason known only to those
who really understand Postscript.

If your laser printer, supposedly
Postcript-compatible, refuses to print the plot file, you might
consider whether the first line of the plot file, which starts with %!
needs to be altered somehow or eliminated. If your Laserwriter is hooked to
a Macintosh it will be necessary
to persuade it to print the plot file.
In recent versions of the Macintosh operating systems this can supposedly
be done by dragging the file icon onto the printer icon on the desktop.
In earlier versions of the MacOS operating system you might have to use
a utility called the Laserwriter Font Utility, which was distributed with the
operating system.

**PICT format (for drawing programs).** This file format is read by
many drawing programs (an early example was MacDraw). It has support for
some fonts, though if fonts are used the species names can only be drawn
horizontally or vertically, not at other angles in between. The control over
line widths is a bit rough also, so that some lines at different angles
may turn out to be different widths when you do not want them to be.
If you are working on a Macintosh system and have not been able to persuade
it to print a Postscript file, this option may be the best solution, as you
could then read the file into a drawing program and then order it to print
the resulting screen. The PICT file format has font support, and the
default font for this plotting option is set to Times. You can also
choose font attributes for the labels such as Bold, Italic, Outline, and
Shadowed.

**HP Laserjet PCL file format.** Hewlett-Packard's extremely popular line
of laser printers has been emulated by many other brands of laser printer,
so that this format is compatible with more printers than any other.
One limitation of the PCL4
command language for these printers is that it does not have primitive
operations for drawing arbitrary diagonal lines. This means that they must
be treated by these programs as if they were dot matrix printers with a
great many dots. This makes output files large, and output can be slow.
The user will be asked to choose
the dot resolution (75, 150, or 300 dots per inch). The 300 dot per inch
setting should not be used if the laser printer's memory is less than
512k bytes. The quality of output is also not as good as it might
be so that the Postscript file format will usually produce better results even
at the same resolution. I am
grateful to Kevin Nixon for inadvertently pointing out that on Laserjets one
does not have to dump the complete bitmap of a page to plot a tree.

**MS-Windows Bitmap.** This file format is used by most Windows
drawing and paint programs, including Windows Paint which comes with the
Windows operating system. It asks you to choose the height and width
of the graphic image in pixels. For the moment, the image is set to be
a monochrome image which can only be black or white. We hope to change
that soon, but note that by pasting the image into a copy of Paint that
is set to have a color image of the appropriate size, one can get a version
whose color can be changed. Note also that Windows Bitmap files can be
used as "wallpaper" images for the background of a desktop.

**IBM PC graphics screens.** This form of graphics was supported on
DOS computers in the pre-Windows era.
The graphics modes supported are CGA, EGA, VGA,
Hercules, and AT&T (Olivetti).
This option is also available for previewing plots, and
in either previewing or final plotting it draws directly on the screen
and does not make a plot file.

**FIG 2.0 drawing program format.** This is the file format of the
free drawing program Xfig, available for X-windows systems on Unix or
Linux systems. Xfig
can be obtained from
http://duke.usask.ca/~macphed/soft/fig/

You should also get transfig, which contains the fig2dev program which converts
xfig output to the various printer languages. Transfig is on the same machine
in

```
    /contrib/R5fixes/transfig-patches/transfig.2.1.6.tar.Z.
```

The present format
does not write the species labels in fonts recognized by
Xfig but draws them with lines. This often makes the names look rather
bumpy. We hope to change this soon.

**Idraw drawing program format.** Idraw is a free drawing program for
X windows systems (such as Unix and Linux systems). Its interface is
loosely based on MacDraw, and I find it much more useable than Xfig.
Though it was unsupported for a number of years, it has more recently
been actively supported by Scott Johnston, of
Vectaport, Inc. (http://www.vectaport.com). He has produced, in his ivtools package, a number
of specialized versions of Idraw, and he also distributes the original
Idraw as part of it. Linux executables for all these are available from
http://www.ivtools.org/ivtools/.

The Idraw file format that our programs produce can be read into Idraw, or
can be imported into the other Ivtools programs. The file format saved
from Idraw (or which can be exported from the other Ivtools programs)
is Postscript, and if one does not print directly from Idraw one can
simply send the file to the printer. But the format we produce is missing
some of the header information and will not work directly as a Postscript
file. However if you read it into Idraw and then save it (or import it into
one of the other Ivtools programs and then export it) you will get a Postscript
version that is fully useable.

Drawgram and Drawtree have font support in their Idraw file format options.
The default font is Times-Bold but you can also enter the name of any
other font that is supported by your Postscript printer. Idraw labels
can be rotated to any angle.

**VRML Virtual Reality Markup Language file.** This is by far the most
interesting plotting file format. VRML files describe objects in 3-dimensional
space with lighting on them. A number of freely available "virtual reality
browsers" such as Cosmo Player or Cortona can read VRML files.
A list of available virtual reality browsers and browser plugins
can be found at http://cic.nist.gov/vrml/vbdetect.html, a site
that also automatically detects which VRML plugins are appropriate for your web
browser.
VRML plugins for your web browser or standalone browsers allow you to
wander around looking at the tree from various angles, including from
behind!  I found
VRMLView particularly easy to download -- it is distributed as an executable.
It is not particulary fast and somewhat mysterious to use (try your mouse
buttons).
At the moment our VRML output is unsophisticated. The branches are
made of tubes, with spheres at their joints. The tree is made of
three-dimensional
tubes but is basically flat. Names are made of connected tubes (to get this
make sure you use a simple default font such as the Hershey font in file
font1). VRML itself has
been superseded by a standard called X3D (see 
http://www.web3d.org/), and we will be moving toward X3D support.
Fortunately X3D is backwards compatible with VRML. What's
next? Trees whose branches stick out in three dimensions? Animated trees
whose forks rotate slowly?
A video game involving combat among schools of systematists?

**PCX file format (for drawing programs).** A bitmap format that was
formerly much used on the PC platform, this has been largely superseded by
the Windows Bitmap (BMP) format, but it is still useful. This file format
is simple and is read by many other programs as well. The user must
choose one of three resolutions for the file, 640x480, 800x600, or 1024x768.
The file is a monochrome paint file. Our PCX format is correct but is not
read correctly by versions of Microsoft Paint (PBrush) that are running on
systems that have loaded Word97.

**Tektronix 4010 graphics terminal.** The plot file will contain commands
for driving the Tektronix series of graphics terminals. Other
graphics terminals were compatible with the Tektronix 4010 and its
immediate descendants. The MSDOS version of the public domain communications
program Kermit, versions 2.30 and later, can emulate a Tektronix
graphics terminal if the command "set terminal tek" is given. Of course
that assumes that you are communicating with another computer. There are
also similar terminal emulation programs for Macintoshes that emulate
Tektronix graphics. On workstations with X windows you can use one option of
the "xterm" utility to create a Tektronix-compatible window. On Sun
workstations there used to be a
Tektronix emulator you can run called "tektool" which can be used to
view the trees. The Tektronix option is also available in our programs
for previewing the plots, in which case the plotting commands will be not be
written into a file but will be sent directly to your terminal.

**X Bitmap format.** This produces an X-bitmap for the X Windows system
on Unix or Linux systems,
which can be displayed on X screens. You will be asked for the
size of the bitmap (e.g., 16x16, or 256x256, etc.). This format
cannot be printed out without further format conversion but is
usable for backgrounds of windows ("wallpaper"). This can be a very
bulky format if you choose a large bitmap. The bitmap is a structure
that can actually be compiled into a C program (and thus built in to it),
if you should have some reason for doing that.

**POVRAY 3D rendering program file.** This produces a file for the
free ray-tracing program POVRay (Persistence of Vision Raytracer),
which is available at
http://www.povray.org/.
It shows a tree floating above a flat landscape. The tree is flat but
made out of tubes (as are the letters of the species names). It
casts a realistic shadow across the landscape. lit from over the left
shoulder of the viewer. You will be asked to confirm the colors of the
tree branches, the species names, the background, and the bottom plane.
These default to Blue, Yellow, White, and White respectively.

**Rayshade 3D rendering program file.** The input format for the
free ray-tracing program "rayshade" which is
available
at http://www-graphics.stanford.edu/~cek/rayshade/rayshade.html
for many kinds of systems. Rayshade
takes files of this format and turns them into color scenes in "raw"
raster format (also called "MTV" format after a raytracing
program of that name). If you get the pbmplus package
(available from
http://sourceforge.net/projects/netpbm/).
and compile it on your system
you can use the "mtvtoppm" and "ppmtogif" programs to convert this
into the widely-used GIF raster format. (the pbmplus package will also
allow you to convert into tiff, pcx and many other formats) The
resultant image will show a tree floating above a landscape, rendered
in a real-looking 3-dimensional scene with shadows and illumination.
It is possible to use Rayshade to
make two scenes that together are a stereo pair. When producing
output for Rayshade you will be asked by the Drawgram or Drawtree
whether you want to reset the values for the colors you want for the
tree, the species names, the background, and the desired resolution.

**Hewlett-Packard pen plotter (HPGL file format).**
This means that the program will generate a
file as its plot file which uses the HPGL graphics language. Hewlett-Packard
7470, 7475, and many other plotters are compatible with this. The
paper size is again assumed to be 8.5 by 11 inches (again, A4 should
work well too). It is assumed that there are two pens, a finer one for
drawing names, and the HPGL commands will call for switching between
these. The Hewlett-Packard Laserjet III printer can emulate an HP plotter,
and this feature is included in its PCL5 command language (but not in the
PCL4 command languages of earlier Hewlett-Packard models). As plotters
are now rare the main use of HPGL will be when they are emulated by
laser printers, but other file formats such as PCL and Postscript will be
better choices in those cases.

**DEC ReGIS graphics (VT240 terminal).** The DEC ReGIS standard is
used by the VT240 and VT340 series terminals by DEC (Digital Equipment
Corporation). There used to be many graphics terminals that emulate the
VT240 or VT340 as well. The DECTerm windows in many versions
of Digital's (now Compaq's) DECWindows windowing system do so.
This option is available in our programs for
previewing trees as well. In preview mode it does not write a plot file
but sends the commands directly to the screen; in final mode it writes a
plot file. In DEC's version of Unix, Ultrix version 4.1 and later,
the windowing system allows DEC ReGIS graphics as a default.

**Epson MX-80 dot-matrix printer.** This file format is for the dot-matrix
printers by Epson (starting with the MX80 and continuing on to many other
models), as well as the IBM Graphics printers. The code here plots in
double-density graphics mode. Many of the later models are capable of
higher-density graphics but not with every dot printed. This
density was chosen for reasonably wide compatibility. Many other dot-matrix
printers on the market have graphics modes compatible with the
Epson printers. I cannot guarantee that the plot files generated by
these programs will be compatible with all of these, but they do work on
Epsons. They have also worked, in our hands, on IBM Graphics Printers. There
used to be many printers that claimed compatibility with these too, but I
do not know whether it will work on all of them. If you have trouble
with any of these you might consider trying in the epson option of
procedure initplotter to put in a fprintf statement
that writes to plotfile an escape sequence that changes line spacing.
As dot matrix printers are rare these days, I suspect this option will
not get much testing.

**Prowriter/Imagewriter dot-matrix printer.**
The trading firm C. Itoh distributed this line of
dot-matrix printers, which was made by Tokyo Electric (TEC) and also was
sold by NEC under the product number PC8023. These were 9-pin dot matrix
printers. In a slightly modified form they were also the Imagewriter
printer sold by Apple for their Macintosh line. The same escape codes
seem to work on both machines, the Apple version being a serial
interface version. They are not related to the IBM Proprinter, despite
the name.

**Toshiba 24-pin dot-matrix printer.**
The 24-pin printers from Toshiba were covered
by this option. These included the P1340, P1350, P1351, P351, 321, and
later models. For a 24-pin printer the plot file can get fairly large
as it contains a bit map of the image and there are more bits with a
24-pin image. Printing was usually slow.

**Okidata dot-matrix printer.**
The ML81, 82, 83 and ML181, 182, 183 line of dot-matrix
printers from Okidata had their own graphics codes and those are dealt
with by this option. The later Okidata ML190 series emulated IBM Graphics
Printers so that you would not want to use this option for them but the option
for that printer.

**Houston Instruments plotter.** The Houston Instruments line of plotters
were also known as Bausch and Lomb plotters. The code in the
programs for these has not been tested recently; I would appreciate
anyone who tries it out telling me whether it works. I do not have
access to such a plotter myself, and doubt most users will come across one.

Conversion from these formats to others is also possible.
There is a free program by Jef Poskanzer called "PBMPLUS" that interconverts
many bitmap formats (see above under Rayshade).

## Drivers for Preview of Plots

Plots may be previewed in a number of formats which are chosen using the
menu option. Previewing defaults to different drivers depending on which
kind of system you are running the programs on. For Unix or Linux systems
it defaults to X Windows, for Windows systems to Windows graphics, and
for Macintosh systems to macintosh graphics screens.

We have already mentioned (above) some of the options that are also used
for previewing. These include:

**MSDOS Graphics Screens.** These were mentioned
above as possible output images.

**Macintosh graphics screens.** Using the windowing features of
Codewarrior C from Metrowerks, our Macintosh executables
open a graphics window and draw preview trees in it. We have
not provided this option for final plotting of the tree.
The window is about 2/3 the height of the desktop screen and has the tree drawn
in black on a white background.
After the preview appears, you can dismiss the window by closing it
using the usual little box in its corner, or by typing Command-Q.

**X Windows display.** Our Unix and Linux code tries to do previews
in X Windows. We hope that the Unix/Linux Makefle will find the correct
libraries to link from. An X window appears with the preview of the tree
in it. To dismiss this window one needs to put the mouse over the
text window that had the menus in it (or click on them) and then type Y
or N to plot the tree or return to the menu.

**MS Windows display.** The executables produced using the Cygwin
Gnu C++ compiler should produce this graphics preview window. The preview
window can be dismissed using its File menu. In its menu the Change
Parameters otion will lead you back to the text menu to make more
changes, and the Plot option will cause the final plot file to be written.
The Quit option will interrupt the program, causing no plot file to b produced.
Normally you will not want to use that option.

**Tektronix 4010 graphics terminal.** This previewing option was
described above as a final plot option.

**DEC ReGIS graphics (VT240 terminal).** This previewing option was
described above as a final plot option.

## Problems Copying Files to Printers

A problem may arose in how to get the plot files to the plotting device
or printer. One has to copy them directly, but one should be careful to
not let your serial or parallel port strip off the high-order bits in the
bytes if you are using one of the options that generate nonprintable
characters. This will be true for most of the dot matrix printers and
for bitmaps dumped to an HP Laserjet-compatible printer. This can be
a problem under Unix or MSDOS. If, for example, you have a dot-matrix
printer connected to a parallel port under PCDOS, to copy the file
PLOTFILE to the printer without losing the high-order bits, you must use
the /B switch on the COPY command:

```
  COPY/B PLOTFILE PRN:
```

## The VAX VMS Line Length Problem

A problem that may occur under some operating systems, particularly the
VMS operating system for Digital VAXes, is having a plot file with lines
that exceed some operating system limit such as 255 characters. This can
happen if you are using the Tektronix option.
You should set your terminal type with the
command

```
   $ SET TERM/NOWRAP/ESCAPE
```

  
which will allow Tektronix and DEC ReGIS
plots to successfully appear on your terminal. That way, if you have a
terminal capable of plotting one of these kinds of plots, the operating
system will not interfere with the process. It will not be possible to
use files of Tektronix commands as final plot files, however, as the TYPE
command usually used to get them to appear on the screen does not allow
lines longer than 2048 bytes, and Tektronix plots are single lines longer
than that.

## Other problems and opportunities

Another problem is adding labels (such as vertical scales and branch
lengths) to the plots produced by this program. This may require you to
use the BMP, PICT, Idrawm, Xfig, PCX or Postscript file format and use a draw
or paint program to add them.

I would like to add more fonts. The present fonts are recoded versions of
the Hershey fonts. They are legally publicly distributable. Most other font
families on the market are not public domain and I cannot
afford to license them for distribution. Some people have noticed that the
Hershey fonts, which are drawn by a series of straight lines, have noticeable
angles in what are supposed to be curves, when they are printed on modern
laser printers and looked at closely. This is less a problem than one might
think since, fortunately, when scientific journals print a tree it is usually
shrunk so small that these imperfections (and often the tree itself)
are hard to see!

One more font that could be added from the Hershey font collection would be a
Greek font. If Greek users would
find that useful I could add it, but my impression is that they publish
mostly in English anyway.

## Writing Code for a new Plotter, Printer or File Format

The C version of these programs consists of two C programs, "drawgram.c"
and "drawtree.c". Each of these has common sections of code compiled
into it called
"draw.c", "draw2.c" and a common header file, "draw.h". In addition
the Macintosh version requires two more files, "interface.c" and
"interface.h". All of the graphics commands that are common to both
programs will be found in "draw.c" and "draw2.c". The following instructions
for writing your own code to drive a different kind of printer,
plotter, or graphics file format, require you only to make changes in
"draw.c" and "draw2.c". The two programs can then be recompiled.

If you want to write code for other printers, plotters, or vector
file formats, this is not
too hard. The plotter option "U" is provided as a place for you to insert
your own code. Chris Meacham's system was to draw everything, including the
characters in the names and all curves, by drawing a series of straight
lines. Thus you need only master your plotter's commands for drawing
straight lines. In function "plotrparms"
you must set up the values of
variables "xunitspercm" and "yunitspercm", which are the number of units in
the x and y directions per centimeter, as well as variables "xsize" and
"ysize" which are the size of the plotting area in centimeters in the x
direction and the y direction. A variable "penchange" of a user-defined type
is set to "yes" or "no" depending on whether the commands to change the pen
must be issued when switching between plotting lines and drawing
characters. Even though dot-matrix printers do not have pens, penchange
should be set to "yes" for them. In function "plot" you must issue commands
to draw a line from the current
position (which is at (xnow, ynow) in the plotter's units) to the position
(xabs, yabs), under the
convention that the lower-left corner of the plotting area is (0.0, 0.0). In
functions "initplotter" and "finishplotter"
you must issue commands to
initialize the plotter and to finish plotting, respectively. If the pen is
to be changed an appropriate piece of code must be inserted in
function "penchange".
The code to print the text needs to be added to the "plottext" function.

For dot matrix printers and raster graphics matters are a bit more complex. The
functions "plotrparms", "initplotter", "finishplotter" and "plot"
still respectively set up the parameters for the plotter, initialize it,
finish a plot, and plot one line. But now the plotting consists of drawing
dots into a two-dimensional array called "stripe". Once the plot is
finished this array is printed out. In most cases the array is not as tall
as a full plot: instead it is a rectangular strip across it. When the
program has finished drawing in ther strip, it prints it out and then
moves down the plot to the next strip. For example, for Hewlett-Packard
Laserjets we have defined the strip as 2550 dots wide and 20 dots deep. When
the program goes to draw a line, it draws it into the strip and ignores
any part of it that falls outside the strip. Thus the program does a complete
plotting into the strip, then prints it, then moves down the diagram by (in
this case) 20 dots, then does a complete plot into that strip, and so on.

To work with a new raster or dot matrix format, you will have to define the
desired width of a strip ("strpwide"), the desired depth ("strpdeep"), and
how many lines of bytes must be printed out to print a strip. For example
Toshiba P351 printers in graphics mode print strips of dots 1350 bits wide
by 24 bits deep, each column of 24 bits printing out as consecutive four bytes
with 6 bits each. In that case, one prints out a
strip by printing up to 1350 groups of 4 bytes. "strpdiv" is 4, and
"strpwide" is 1350, and "strpdeep" is 24. Procedure "striprint"
is the one that prints out a strip, and has special-case code for the
different printers and file formats. For file formats, all of which
print out a single row of dots at a time, the variable "strpdiv" is not
used. The variable "dotmatrix" is set to
"true" or "false" in function "plotrparms"
according to whether or not "strpdiv" is to be used. Procedure "plotdot"
sets a single dot in the array "strip" to 1 at position (xabs, yabs). The
coordinates run from 1 at the top of the plot to larger numbers as we
proceed down the page. Again, there is special-case code for different
printers and file formats in that
function.
You will probably want to read the code for some of the dot matrix or file
format options if you want to write code for one of them. Many of them
have provision for printing only part of a line, ignoring parts of it that
have no dots to print.

I would be happy to obtain the resulting code from you to consider
adding it to this listing so we can cover more kinds of plotters, printers,
and file formats.

---

## APPENDIX 1. Code to drive some other graphics devices.

These pieces of code are to be
inserted in the places reserved for the "Y" plotter option. The variables
necessary to run this
have already been incorporated into the
programs.

### Calcomp plotters:

Calcomp's industrial-strength plotters are not as much a fixture of
University computer centers as they once were, but just in case you need to
use one, this code should work:

A global declaration needed near the front of drawtree.c:

```
Char cchex[16];
```

Code to be inserted into function plotrparms:

```
  case 'Y':
    plotter = other;
    xunitspercm = 39.37;
    yunitspercm = 39.37;
    xsize = 25.0;
    ysize = 25.0;
    xposition = 12.5;
    yposition = 0.0;
    xoption = center;
    yoption = above;
    rotation = 0.0;
    break;
```

Code to be inserted into function plot:

Declare these variables at the beginning of the function:

```
long n, inc, xinc, yinc, xlast, ylast, xrel,
   yrel, xhigh, yhigh, xlow, ylow;
Char quadrant;
```

and insert this into the switch statement:

```
  case other:
    if (penstatus == pendown)
      putc('H', plotfile);
    else
      putc('D', plotfile);
    xrel = (long)floor(xabs + 0.5) - xnow;
    yrel = (long)floor(yabs + 0.5) - ynow;
    xnow = (long)floor(xabs + 0.5);
    ynow = (long)floor(yabs + 0.5);
    if (xrel > 0) {
      if (yrel > 0)
        quadrant = 'P';
      else
        quadrant = 'T';
    } else if (yrel > 0)
      quadrant = 'X';
    else
      quadrant = '1';
    xrel = labs(xrel);
    yrel = labs(yrel);
    if (xrel > yrel)
      n = xrel / 255 + 1;
    else
      n = yrel / 255 + 1;
    xinc = xrel / n;
    yinc = yrel / n;
    xlast = xrel % n;
    ylast = yrel % n;
    xhigh = xinc / 16;
    yhigh = yinc / 16;
    xlow = xinc & 15;
    ylow = yinc & 15;
    for (i = 1; i <= n; i++)
      fprintf(plotfile, "%c%c%c%c%c",
              quadrant, cchex[xhigh - 1], cchex[xlow - 1], cchex[yhigh - 1],
              cchex[ylow - 1]);
    if (xlast != 0 || ylast != 0)
      fprintf(plotfile, "%c%c%c%c%c",
              quadrant, cchex[-1], cchex[xlast - 1], cchex[-1],
              cchex[ylast - 1]);
    break;
```

Code to be inserted into function initplotter:

```
  case other:
    cchex[-1] = 'C';
    cchex[0] = 'D';
    cchex[1] = 'H';
    cchex[2] = 'L';
    cchex[3] = 'P';
    cchex[4] = 'T';
    cchex[5] = 'X';
    cchex[6] = '1';
    cchex[7] = '5';
    cchex[8] = '9';
    cchex[9] = '/';
    cchex[10] = '=';
    cchex[11] = '#';
    cchex[12] = '"';
    cchex[13] = '\'';
    cchex[14] = '^';
    xnow = 0.0;
    ynow = 0.0;
    fprintf(plotfile, "CCCCCCCCCC");
    break;
```

Code to be inserted into function finishplotter:

```
  case other:
    plot(penup, 0.0, yrange + 50.0);
    break;
```

---

## Appendix 2. Our Hershey font encoding.

The Hershey fonts were digitized fonts created by Dr. A. V. Hershey in the
late 1960s when he was working at the U. S. Naval Weapons Laboratory.
They were published in U. S. National Bureau of Standards Special
Publication No. 424, distributed by the U. S. National Technical Information
Service.
Legally, it is possible to freely distribute these fonts in any encoding
system *except* the original one used by the U. S. National
Technical Information Service, provided that you acknowledge that the original
fonts were produced by Dr. Hershey and published by NBS. Fortunately, Chris
Meacham developed the
software we use to read the Hershey fonts, and it uses a simple coding
system that he developed. The original Hershey fonts were transformed by
him into this encoding system. Six of them are distributed with PHYLIP: three
Roman fonts, one unserifed and two serifed, two Italic fonts, one unserifed and
one serifed, and a Russian Cyrillic font.

Each font file consists of groups of lines, one for each character. Here are
the lines for character "h" in the font #1 in this encoding:

```
Ch 608 21 19 28
 -1456 1435 -1445 1748 1949 2249 2448 2545 2535 -12935
```

The group of lines starts with the letter C (for Character). Then follows
the character that this font will draw (in this case "h"). It is the byte
which, when read by the computer, signals that character. Then there is
the number of this character in the original Hershey fonts (608). This is
not used by our software.

The Hershey fonts are drawn on a grid of points as a series of lines.
The next three numbers (21, 19, and 28) are the height (21), and two
widths (19, and 28, which we don't use). Then comes a new line which
shows the individual pen moves. When these are negative, they indicate
that the pen is to be up when moving; when they are positive, the pen is
to be down. They are integers. The last of them is greater than 10,000,
and that is the signal to end after that move.

Each number has a final four digits that give the coordinate to which the
pen is to move. These are given as (x,y) coordinates. Thus the first
number (-1456) indicates the pen is to be up and the plotting is to move to
coordinate (14, 56), which is  x = 14, y = 56. Then the pen is put
down and moved to (14, 35). This draws a line from (14, 56) to (14, 35),
in fact the vertical line that forms the back of the "h". Then the
pen is picked up and moved to (14, 45). Then there follow a series of moves
with pen down to (14, 35), (14, 45), (17, 48), (19, 49), (22, 49),
(24, 48), (25, 45), and finally (25, 35). This draws a series of connected
line segments that make the arch and right-hand
vertical, ending up at the bottom-right of the character. -12935 then
signals a pen-up move to (29, 35). This moves to a point where the next
character can start, putting in a little "white space".

As you can see, the coding system is quite simple. Does anyone want to
draw us some new fonts to add to our repertoire? I have spared you the
Gothic, Old English, and Greek Hershey fonts, but perhaps there are some
other nice ones people might want to use.

---
