# Supplementary material for: MSOAR 2.0: Incorporating tandem duplications into ortholog assignment based on genome rearrangement
Source: BMC Bioinformatics. 2010 Jan 6;11:10. doi: 10.1186/1471-2105-11-10 (PMC2821317; doi:10.1186/1471-2105-11-10)
Supplement: Additional file 2 — contains the software MSOAR 2.0. [file 1471-2105-11-10-S2.GZ › MSOAR2.0/Programs/phylip-3.67/doc/distance.html]

distance


version 3.66

# Distance matrix programs

© Copyright 1986-2006 by the University of Washington. Written by Joseph
Felsenstein. Permission is granted to copy
this document provided that no fee is charged for it and that this copyright
notice is not removed.

The programs Fitch, Kitsch, and Neighbor are for dealing with data which
comes in the form of a matrix of pairwise distances between all pairs of taxa,
such as distances based on molecular sequence data,
gene frequency genetic distances, amounts of DNA hybridization, or
immunological distances. In analyzing these
data, distance matrix programs implicitly assume that:

1. Each distance is measured independently from the others: no item of
   data contributes to more than one distance.- The distance between each pair of taxa is drawn from a distribution
     with an expectation which is the sum of values (in effect amounts of evolution)
     along the tree from one tip to the other. The variance of the distribution is
     proportional to a power *p* of the expectation.

These assumptions can be traced in the least squares methods of programs
Fitch and Kitsch but it is not quite so easy to see them in operation in the
Neighbor-Joining method of Neighbor, where the independence assumptions is less
obvious.

THESE TWO ASSUMPTIONS ARE DUBIOUS IN MOST CASES: independence will not be
expected to be true in most kinds of data, such as genetic distances from gene
frequency data. For genetic distance data in which pure genetic drift without
mutation can be assumed to be the mechanism of change Contml may be more
appropriate. However, Fitch, Kitsch, and Neighbor will not give positively
misleading results (they will not make a statistically inconsistent estimate)
provided that additivity holds, which it will if the distance is computed from
the original data by a method which corrects for reversals and parallelisms in
evolution. If additivity is not expected to hold, problems are more severe. A
short discussion of these matters will be found in a review article of mine
(1984a). For detailed, if sometimes irrelevant, controversy see the papers by
Farris (1981, 1985, 1986) and myself (1986, 1988b).

For genetic distances from gene frequencies, Fitch, Kitsch, and Neighbor
may be appropriate if a neutral mutation model can be assumed and Nei's genetic
distance is used, or if pure drift can be assumed and either Cavalli-Sforza's
chord measure or Reynolds, Weir, and Cockerham's (1983) genetic distance is
used. However, in the latter case (pure drift) Contml should be better.

Restriction site and restriction fragment data can be treated by distance
matrix methods if a distance such as that of Nei and Li (1979) is used.
Distances of this sort can be computed in PHYLIP by the program Restdist.

For nucleic acid sequences, the distances computed in Dnadist allow
correction for multiple hits (in different ways) and should allow one to
analyse the data under the presumption of additivity. *In all of these cases
independence will not be expected to hold*. DNA hybridization and
immunological distances may be additive and independent if transformed properly
and if (and only if) the standards against which each value is measured are
independent. (This is rarely exactly true).

Fitch and the Neighbor-Joining option of Neighbor fit a tree which has the
branch lengths unconstrained. Kitsch and the UPGMA option of Neighbor, by
contrast, assume that an "evolutionary clock" is valid, according to which the
true branch lengths from the root of the tree to each tip are the same: the
expected amount of evolution in any lineage is proportional to elapsed time.

The input format for distance data is straightforward. The first line of
the input file contains the number of species. There follows species data,
starting, as with all other programs, with a species name. The species name is
ten characters long, and must be padded out with blanks if shorter. For each
species there then follows a set of distances to all the other species (options
selected in the programs' menus
allow the distance matrix to be upper or lower triangular or square).
The distances can continue to a new line after any of them. If the matrix
is lower-triangular, the diagonal entries (the distances from a species to
itself) will not be read by the programs. If they are included anyway, they
will be ignored by the programs, except for the case where one of them
starts a new line, in which case the program will mistake it for a species
name and get very confused.

For example, here is a sample input matrix, with a square matrix:

```
     5
Alpha      0.000 1.000 2.000 3.000 3.000
Beta       1.000 0.000 2.000 3.000 3.000
Gamma      2.000 2.000 0.000 3.000 3.000
Delta      3.000 3.000 0.000 0.000 1.000
Epsilon    3.000 3.000 3.000 1.000 0.000
```

and here is a sample lower-triangular input matrix with distances continuing
to new lines as needed:

```
   14
Mouse     
Bovine      1.7043
Lemur       2.0235  1.1901
Tarsier     2.1378  1.3287  1.2905
Squir Monk  1.5232  1.2423  1.3199  1.7878
Jpn Macaq   1.8261  1.2508  1.3887  1.3137  1.0642
Rhesus Mac  1.9182  1.2536  1.4658  1.3788  1.1124  0.1022
Crab-E.Mac  2.0039  1.3066  1.4826  1.3826  0.9832  0.2061  0.2681
BarbMacaq   1.9431  1.2827  1.4502  1.4543  1.0629  0.3895  0.3930  0.3665
Gibbon      1.9663  1.3296  1.8708  1.6683  0.9228  0.8035  0.7109  0.8132
  0.7858
Orang       2.0593  1.2005  1.5356  1.6606  1.0681  0.7239  0.7290  0.7894
  0.7140  0.7095
Gorilla     1.6664  1.3460  1.4577  1.5935  0.9127  0.7278  0.7412  0.8763
  0.7966  0.5959  0.4604
Chimp       1.7320  1.3757  1.7803  1.7119  1.0635  0.7899  0.8742  0.8868
  0.8288  0.6213  0.5065  0.3502
Human       1.7101  1.3956  1.6661  1.7599  1.0557  0.6933  0.7118  0.7589
  0.8542  0.5612  0.4700  0.3097  0.2712
```

Note that the name "Mouse" in this matrix must be padded out by blanks to the
full length of 10 characters.

In general the distances are assumed to all be present: at the moment
there is only one way we can have missing entries in the distance matrix. If
the S option (which allows the user to specify the degree of replication of
each distance) is invoked, with some of the entries having degree of
replication zero, if the U (User Tree) option is in effect, and if the tree
being examined is such that every branch length can be estimated from the data,
it will be possible to solve for the branch lengths and sum of squares when
there is some missing data. You may not get away with this if the U option is
not in effect, as a tree may be tried on which the program will calculate a
branch length by dividing zero by zero, and get upset.

The present version of Neighbor does allow the Subreplication option to be
used and the number of replicates to be in the input file, but it actally does
nothing with this information except read it in. It makes use of the average
distances in the cells of the input data matrix. This means that you cannot use
the S option to treat zero cells. We hope to modify Neighbor in the future to
allow Subreplication. Of course the U (User tree) option is not available in
Neighbor in any case.

The present versions of Fitch and Kitsch will do much better on missing
values than did previous versions, but you will still have to be careful about
them. Nevertheless you might (just) be able to explore relevant alternative
tree topologies one at a time using the U option when there is missing data.

Alternatively, if the missing values in one cell always correspond to a
cell with non-missing values on the opposite side of the main diagonal (i.e.,
if D(i,j) missing implies that D(j,i) is not missing), then use of the S option
will always be sufficient to cope with missing values. When it is used, the
missing distances should be entered as if present (any number can be
used) and the degree of replication for them should be given as 0.

Note that the algorithm for searching among topologies in Fitch and Kitsch
is the same one used in other programs, so that it is necessary to try
different orders of species in the input data. The J (Jumble) menu option may
be sufficient for most purposes.

The programs Fitch and Kitsch carry out the method of Fitch and Margoliash
(1967) for fitting trees to distance matrices. They also are able to carry out
the least squares method of Cavalli-Sforza and Edwards (1967), plus a variety
of other methods of the same family (see the discussion of the P option below).
They can also be set to use the Minimum Evolution method (Nei and Rzhetsky,
1993; Kidd and Sgaramella-Zonta, 1971).

The objective of these methods is to find that tree which minimizes

```
                      __  __
                      \   \    nij ( Dij  - dij)2  
  Sum of squares  =   /_  /_  ------------------
                       i   j       Dijp
```

(the symbol made up of \, / and \_ characters is of course a summation sign)
where *D* is the observed distance between species *i* and
*j* and *d* is the expected
distance, computed as the sum of the lengths (amounts of evolution) of the
segments of the tree from species *i* to species *j*. The
quantity *n* is the number
of times each distance has been replicated. In simple cases this is taken to
be one, but the user can, as an option, specify the degree of replication for
each distance. The distance is then assumed to be a mean of those replicates.
The power *P* is what distinguished the various methods. For the Fitch-
Margoliash method, which is the default method with this program, *P*
is 2.0. For the Cavalli-Sforza and Edwards least squares method it should be set to 0
(so that the denominator is always 1). An intermediate method is also
available in which P is 1.0, and any other value of P, such as 4.0 or -2.3, can
also be used. This generates a whole family of methods.

The P (Power) option is not available in the Neighbor-Joining program
Neighbor. Implicitly, in this program P is 0.0 (though it is hard to prove
this). The UPGMA option of Neighbor will assign the same branch lengths to the
particular tree topology that it finds as will Kitsch when given the same tree
and Power = 0.0.

All these methods make the assumptions of additivity and independent
errors. The difference between the methods is how they weight departures of
observed from expected. In effect, these methods differ in how they assume
that the variance of measurement of a distance will rise as a function of the
expected value of the distance.

These methods assume that the variance of the measurement error is
proportional to the *P*-th power of the expectation (hence the standard deviation
will be proportional to the *P/2*-th power of the expectation). If
you have
reason to think that the measurement error of a distance is the same for small
distances as it is for large, then you should set *P=0* and use the
least squares
method, but if you have reason to think that the relative (percentage) error is
more nearly constant than the absolute error, you should use *P=2*,
the Fitch-Margoliash method. In between, P=1 would be appropriate if the
sizes of the errors were proportional to the square roots of the expected
distance.

One question which arises frequently is what the units of branch length are
in the resulting trees. In general, they are not time but units of
distance. Thus if two species have a distance 0.3 between them, they will
tend to be separated by branches whose total length is about 0.3. In the
case of DNA distances, for example, the unit of branch length will be
substitutions per base. (In the case of protein distances, it will be
amino acid substitutions per amino acid position.)

## OPTIONS

Here are the options available in all three programs. They are selected using
the menu of options.

U: the User tree option. The trees in Fitch are regarded as unrooted, and are specified with a trifurcation (three-way split) at their base: e. g.: ((A,B),C,(D,E)); while in Kitsch they are to be regarded as rooted and have a bifurcation at the base: ((A,B),(C,(D,E))); Be careful not to move User trees from Fitch to Kitsch without changing their form appropriately (you can use Retree to do this). User trees are not available in Neighbor. In Fitch if you specify the branch lengths on one or more branches, you can select the L (use branch Lengths) option to avoid having those branches iterated, so that the tree is evaluated with their lengths fixed. P: indicates that you are going to set the Power (P in the above formula). The default value is 2 (the Fitch-Margoliash method). The power, a real number such as 1.0, is prompted for by the programs. This option is not available in Neighbor. -: indicates that negative segment lengths are to be allowed in the tree (default is to require that all branch lengths be nonnegative). This option is not available in Neighbor. O: is the usual Outgroup option, available in Fitch and Neighbor but not in Kitsch, nor when the UPGMA option of Neighbor is used. L: indicates that the distance matrix is input in Lower-triangular form (the lower-left half of the distance matrix only, without the zero diagonal elements). R: indicates that the distance matrix is input in uppeR-triangular form (the upper-right half of the distance matrix only, without the zero diagonal elements). S: is the Subreplication option. It informs the program that after each distance will be provided an integer indicating that the distance is a mean of that many replicates. There is no auxiliary information, but the presence of the S option indicates that the data will be in a different form. Each distance must be followed by an integer indicating the number of replicates, so that a line of data looks like this: ``` Delta 3.00 5 3.21 3 1.84 9 ``` the 5, 3, and 9 being the number of times the measurement was replicated. When the number of replicates is zero, a distance value must still be provided, although its vale will not afect the result. This option is not available in Neighbor. G: is the usual Global branch-swapping option. It is available in Fitch and Kitsch but is not relevant to Neighbor. J: indicates the usual J (Jumble) option for entering species in a random order. In Fitch and Kitsch if you do multiple jumbles in one run the program will print out the best tree found overall. M: is the usal Multiple data sets option, available in all of these programs. It allows us (when the output tree file is analyzed in Consense) to do a bootstrap (or delete-half-jackknife) analysis with the distance matrix{ programs.

The numerical options are the usual ones and should be clear from the
menu.

Note that when the options L or R are used one of the species, the first
or last one, will have its name on an otherwise empty line. Even so, the name
should be padded out to full length with blanks. Here is a sample lower-
triangular data set.

|  |  |
| --- | --- |
| ```      5 Alpha       Beta       1.00 Gamma      3.00 3.00 Delta      3.00 3.00 2.00 Epsilon    3.00 3.00 2.00 1.00 ``` | <--- note: five blanks should follow the name "Alpha" |

Be careful if you are using lower- or upper-triangular trees to make the
corresponding selection from the menu (L or R), as the
program may get horribly confused otherwise, *but it still gives a result even
though the result is then meaningless*. With the menu option selected all
should be well.
