# Supplementary material for: MSOAR 2.0: Incorporating tandem duplications into ortholog assignment based on genome rearrangement
Source: BMC Bioinformatics. 2010 Jan 6;11:10. doi: 10.1186/1471-2105-11-10 (PMC2821317; doi:10.1186/1471-2105-11-10)
Supplement: Additional file 2 — contains the software MSOAR 2.0. [file 1471-2105-11-10-S2.GZ › MSOAR2.0/Programs/phylip-3.67/doc/gendist.html]

gendist


version 3.66

# Gendist - Compute genetic distances from gene frequencies

© Copyright 1986-2006 by the University of
Washington. Written by Joseph Felsenstein. Permission is granted to copy
this document provided that no fee is charged for it and that this copyright
notice is not removed.

This program computes any one of three measures of genetic distance from a set
of gene frequencies in different populations (or species). The three are
Nei's genetic distance (Nei, 1972), Cavalli-Sforza's chord measure (Cavalli-
Sforza and Edwards, 1967) and Reynolds, Weir, and Cockerham's (1983) genetic
distance. These are written to an output file in a format that can be read by
the distance matrix phylogeny programs Fitch and Kitsch.

The three measures have somewhat different assumptions. All assume that all
differences between populations arise from genetic drift. Nei's distance is
formulated for an infinite isoalleles model of mutation, in which there is a
rate of neutral mutation and each mutant is to a completely new alleles. It
is assumed that all loci have the same rate of neutral mutation, and that the
genetic variability initially in the population is at equilibrium between
mutation and genetic drift, with the effective population size of each
population remaining constant.

Nei's distance is:

```
                                      \   \
                                      /_  /_  p1mi   p2mi
                                       m   i
           D  =  - ln  ( ------------------------------------- ).
                                                                   
                           \   \                \   \
                         [ /_  /_  p1mi2]1/2   [ /_  /_  p2mi2]1/2     
                            m   i                m   i
```

where *m* is summed over loci, *i* over alleles at the *m*-th locus, and where

     p_1mi_

is the frequency of the *i*-th allele at the *m*-th locus in population 1.
Subject to the above assumptions, Nei's genetic distance is expected, for a
sample of sufficiently many equivalent loci, to rise linearly with time.

The other two genetic distances assume that there is no mutation, and that all
gene frequency changes are by genetic drift alone. However they do not
assume that population sizes have remained constant and equal in all
populations. They cope with changing population size by having expectations
that rise linearly not with time, but with the sum over time of 1/N, where N
is the effective population size. Thus if population size doubles, genetic
drift will be taking place more slowly, and the genetic distance will be
expected to be rising only half as fast with respect to time. Both genetic
distances are different estimators of the same quantity under the same model.

Cavalli-Sforza's chord distance is given by

```
                   \               \                        \
     D2    =    4  /_  [  1   -    /_   p1mi1/2 p 2mi1/2]  /  /_  (am  - 1)
                    m               i                        m
```

where m indexes the loci, where i is summed over the alleles at the m-th
locus, and where a is the number of alleles at the m-th locus. It can be
shown that this distance always satisfies the triangle
inequality. Note that as given here it is divided by the number of degrees
of freedom, the sum of the numbers of alleles minus one. The quantity which
is expected to rise linearly with amount of
genetic drift (sum of *1/N* over time) is *D* squared, the quantity computed
above, and that is what is written out into the distance matrix.

Reynolds, Weir, and Cockerham's (1983) genetic distance is

```
                       \    \
                       /_   /_  [ p1mi     -  p2mi]2
                        m    i                  
       D2     =      --------------------------------------
                                           
                         \               \
                      2  /_   [  1   -   /_  p1mi    p2mi ]
                          m               i
```

where the notation is as before and D^2^ is the quantity that is
expected to rise linearly with cumulated genetic drift.

Having computed one of these genetic distances, one which you feel is
appropriate to the biology of the situation, you can use it as the input to
the programs Fitch, Kitsch or Neighbor. Keep in mind that the statistical
model in
those programs implicitly assumes that the distances in the input table have
independent errors. For any measure of genetic distance this will not be true,
as bursts of random genetic drift, or sampling events in drawing the sample of
individuals from each population, cause fluctuations of gene frequency that
affect many distances simultaneously. While this is not expected to bias the
estimate of the phylogeny, it does mean that the weighing of evidence from all
the different distances in the table will not be done with maximal
efficiency. One issue is which value of the P
(Power) parameter should be used. This depends on how the variance of a
distance rises with its expectation. For Cavalli-Sforza's chord distance, and
for the Reynolds et. al. distance it can be shown that the variance of the
distance will be proportional to the square of its expectation; this suggests
a value of 2 for *P*, which the default value for Fitch and Kitsch
(there is no P option in Neighbor).

If you think that the pure genetic drift model is appropriate, and are thus
tempted to use the Cavalli-Sforza or Reynolds et. al. distances, you might
consider using the maximum likelihood program Contml instead. It will
correctly weigh the evidence in that case. Like those genetic distances, it
uses approximations that break down as loci start to drift all the way to
fixation. Although Nei's distance will not break down in that case, it
makes other assumptions about equality of substitution rates at all loci and
constancy of population sizes.

The most important thing to remember is that genetic distance is not an
abstract, idealized measure of "differentness". It is an estimate of a
parameter (time or cumulated inverse effective population size) of the
model which is thought to have generated the differences we see. As an
estimate, it has statistical properties that can be assessed, and we should
never have to choose between genetic distances based on their aesthetic
properties, or on the personal prestige of their originators. Considering them
as estimates
focuses us on the questions which genetic distances are intended to answer,
for if there are none there is no reason to compute them. For further
perspective on genetic distances, I recommend my own paper evaluating
different genetic distances (Felsenstein, 1985c),
Reynolds, Weir, and Cockerham (1983), and the material in Nei's book (Nei,
1987).

## INPUT FORMAT

The input to this program is standard and is as described in the Gene
Frequencies and Continuous
Characters Programs documentation file above. It consists of the number of
populations (or species), the number of loci,
and after that a line containing the numbers of alleles at each of the
loci. Then the gene frequencies follow in standard format.

The options are selected using a menu:

```
Genetic Distance Matrix program, version 3.6

Settings for this run:
  A   Input file contains all alleles at each locus?  One omitted at each locus
  N                        Use Nei genetic distance?  Yes
  C                Use Cavalli-Sforza chord measure?  No
  R                   Use Reynolds genetic distance?  No
  L                         Form of distance matrix?  Square
  M                      Analyze multiple data sets?  No
  0              Terminal type (IBM PC, ANSI, none)?  ANSI
  1            Print indications of progress of run?  Yes

  Y to accept these or type the letter for one to change
```

The A (All alleles) option is described in the Gene Frequencies and
Continuous Characters Programs documentation file. As with Contml, it is
the signal that all alleles are represented in the gene frequency input,
without one being left out per locus. C, N, and R are the signals to
use the Cavalli-Sforza, Nei, or Reynolds et. al. genetic distances
respectively. The Nei distance is the default, and it will be computed
if none of these options is explicitly invoked. The L option is the signal
that the distance matrix is to be written out in Lower triangular form.
The M option is the usual Multiple Data Sets option, useful for
doing bootstrap analyses with the distance matrix programs. It allows
multiple data sets, but does not allow multiple sets of weights (since
there is no provision for weighting in this program).

## OUTPUT FORMAT

The output file simply contains on its first line the number of species (or
populations). Each
species (or population) starts a new line, with its name printed out
first, and then and there are up to nine
genetic distances printed on each line, in the standard format used as input
by the distance matrix programs. The output, in its default form, is
ready to be used in the distance matrix programs.

## CONSTANTS

A constant "epsilong" is
available to be changed by the user if the program is recompiled
which defines a small quantity that is used when checking
whether allele frequencies at a locus sum to more than one: if all
alleles are input (option A) and the sum differs from 1 by more than epsilong,
or if not all alleles are input and the sum is greater than 1 by more
then epsilon, the program will see this as an error and stop. You may
find this causes difficulties if you gene frequencies have been rounded.
I have tried to keep epsilong from being too small to prevent such problems.

## RUN TIMES

The program is quite fast and the user should effectively never be limited by
the amount of time it takes. All that the program has to do is read in the
gene frequency data and then, for each pair of species, compute a genetic
distance formula for each pair of species. This should require an amount of
effort proportional to the total number of alleles over loci, and to the
square of the number of populations.

## FUTURE OF THIS PROGRAM

The main change that will be made to this program in the future is to add
provisions for taking into account the sample size for each population. The
genetic distance formulas have been modified by their inventors to correct for
the inaccuracy of the estimate of the genetic distances, which on the whole
should artificially increase the distance between populations by a small
amount dependent on the sample sizes. The main difficulty with doing this is
that I have not yet settled on a format for putting the sample size in the
input data along with the gene frequency data for a species or population.

I may also include other distance measures, but only if I think their use is
justified. There are many very arbitrary genetic distances, and I am
reluctant to include most of them.

---

### TEST DATA SET

```
    5    10
2 2 2 2 2 2 2 2 2 2
European   0.2868 0.5684 0.4422 0.4286 0.3828 0.7285 0.6386 0.0205
0.8055 0.5043
African    0.1356 0.4840 0.0602 0.0397 0.5977 0.9675 0.9511 0.0600
0.7582 0.6207
Chinese    0.1628 0.5958 0.7298 1.0000 0.3811 0.7986 0.7782 0.0726
0.7482 0.7334
American   0.0144 0.6990 0.3280 0.7421 0.6606 0.8603 0.7924 0.0000
0.8086 0.8636
Australian 0.1211 0.2274 0.5821 1.0000 0.2018 0.9000 0.9837 0.0396
0.9097 0.2976
```

---

### TEST SET OUTPUT

```
    5
European    0.000000  0.078002  0.080749  0.066805  0.234881
African     0.078002  0.000000  0.234698  0.104975  0.356903
Chinese     0.080749  0.234698  0.000000  0.053879  0.190002
American    0.066805  0.104975  0.053879  0.000000  0.298109
Australian  0.234881  0.356903  0.190002  0.298109  0.000000
```
